# Supplementary material for: Emerging paradigms for target discovery of traditional medicines: A genome-wide pan-GPCR perspective
Source: Innovation (Camb). 2025 Jan 17;6(3):100774. doi: 10.1016/j.xinn.2024.100774 (PMC11910885; doi:10.1016/j.xinn.2024.100774)
Supplement: Document S1. Tables S1–S3 [file mmc1.pdf]

The Innovation, Volume *6*

Supplemental Information

Emerging paradigms for target discovery of traditional medicines: A genome-wide pan-GPCR perspective

Zenghao Bi, Huan Li, Yuting Liang, Dan Sun, Songxin Liu, Wei Chen, Liang Leng, Chi Song, Sanyin Zhang, Zhaotong Cong, and Shilin Chen

Emerging Paradigms for Target Discovery of Traditional Medicines: A Genome-Wide Pan-GPCR Perspective

Zenghao Bi,[1,2,3,6] Huan Li,[2,3,4,6] Yuting Liang,[1,2,3,6] Dan Sun,[2,3] Songxin Liu,[2,3,5] Wei Chen,[2,3] Liang Leng,[2,3] Chi Song,[2,3] Sanyin Zhang,[2,3] Zhaotong Cong,[2,3*] Shilin Chen,[2,3]

[1]School of Pharmacy, Chengdu University of Traditional Chinese Medicine, Chengdu 611137, China

[2]Institute of Herbgenomics, Chengdu University of Traditional Chinese Medicine, Chengdu 611137, China

[3]Innovative Institute of Chinese Medicine and Pharmacy, Chengdu University of Traditional Chinese Medicine, Chengdu 611137, China

[4]College of Pharmacy, Nanjing University of Chinese Medicine, Nanjing 210023, China

[5]School of Pharmacy, Shanghai University of Traditional Chinese Medicine, Shanghai 201203, China

[6]These authors contributed equally

*Correspondence: congzt@cdutcm.edu.cn (Z.C.)

Supplemental Information

Table S1. Direct effects of traditional medicines on the GPCR function reported over the last two decades

Targets	Ligands	Natural origin	Signaling pathway	Detection methods	Type of the ligand	Pharmacological efficacy	References
**5-HT$_{1A}$R**	N-methyllaurotetanine	*Eschscholzia californica*	n.d.	CLBA	Agonist	EC_{50} = 155 nM, K_i = 85 nM	1
**5-HT$_{1A}$R**	Geissoschizine methyl ether	*Uncaria hook*	n.d.	CLBA, GTPγS binding assay, *in vivo* activity assays	Agonist	IC_{50} = 0.904 µM (CLBA), K_i = 0.517 µM (CLBA)	2
**5-HT$_{2A}$R**	Psilocybin (Converted to psilocin *in vivo*)	*Psilocybe* genus	n.d.	Positron emission tomography scan	Agonist	EC_{50} = 1.95 µg/L	3,4
**5-HT$_{2C}$R**	(*R*)-asimilobine	*Stephania tetrandra*	G_q, β-arrestin	CLBA, A-MS, Ca^{2+} mobilization, β-arrestin recruitment, molecular docking	Agonist	EC_{50} = 308 nM (Ca^{2+}), *In vivo*: 30 mg/kg	5
**5-HT$_{5A}$R**	Valerenic acid	*Valeriana officinalis*	n.d.	CLBA, GTP shift assay	Partial agonist	K_i = 10.7 µM (CLBA)	6
**A$_1$R**	Isovaltrate	*Valeriana officinalis*	G_i	CLBA, GTPγS binding assay	Inverse agonist	K_i = 2 µM (CLBA), EC_{50} = 4.6 µM (GTPγS)	7
**A$_1$R**	Olivil derivative	*Valeriana officinalis*	G_i	CLBA, GTPγS binding assay, cAMP accumulation	Partial agonist	K_i = 5.28 µM (CLBA) EC_{50} = 3.98 µM (GTPγS), IC_{50} = 15 µM (cAMP)	8

**$A_{2A}R$**	Curcumin	*Curcuma longa*	G_s	Western blot, platelet aggregation	Agonist	Single conc. (5, 10, 50 μM) induced vasodilator-stimulated phosphoprotein phosphorylation	9
**APLNR**	(-)-Epicatechin	*Camellia sinensis*	β-arrestin	β-arrestin recruitment, AKT phosphorylation, molecular docking	Biased agonist	$K_d = 1.755 \times 10^{-12}$ M (β-arrestin)	10,11
**BRS3**	Oridonin	*Isodon rubescens*	G_q	IP1 accumulation, Ca^{2+} mobilization, β-arrestin recruitment, DMR, molecular docking, *in vivo* activity assays	Agonist	EC_{50} = 0.2 μM (Ca^{2+}), EC_{50} = 0.7 μM (IP1), EC_{50} = 1.4 μM (DMR)	12
**CB1**	Genistein	*Genista tinctoria*, *Sophora davidii*	G_i	CLBA, molecular docking, GTP turnover assay, *in vivo* activity assays	Antagonist	IC_{50} = 375 nM (CLBA)	13-15
**CB1**	α-humulene (1), geraniol (2), linalool (3), β-pinene (4)	*Cannabis sativa*	G_i	CLBA, ERK phosphorylation, cAMP accumulation, β-arrestin recruitment, *in vivo* activity assays	Agonist	IC_{50} = 44.2 μM (CLBA, 2)	16
**CB1, CB2**	4-O-methylhonokiol (1), magnolol (2), honokiol (3), tetrahydromagnolol (4)	*Magnolia grandiflora*	n.d.	CLBA, molecular docking	Agonist	CB1: K_i = 3.85 μM (1), 17.82 μM (2), 14.55 μM (3), 19.08 μM (4), CB2: K_i = 0.29 μM (1), 1.40 μM (2), 1.94 μM (3), 0.99 μM (4)	17
**CB2**	Celastrol	*Tripterygium wilfordii*	G_i, β-arrestin	SLCA, β-arrestin recruitment, Ca^{2+} mobilization, cAMP accumulation, ERK phosphorylation, molecular docking	Agonist	EC_{50} = 1.77 μM (SLCA), EC_{50} = 1.81 μM (Ca^{2+}), EC_{50} = 0.99 μM (cAMP)	18,19

**CCK_2R**	Caripe 11 (GVIPCGESCVFIPCISTVIGCSCKKKVCYRN)	*Carapichea ipecacuanha*	G_q	IP1 accumulation	Partial agonist	EC_{50} = 8.5 μM	20
**CRF_1R**	Caripe 8 (GVIPCGESCVFIPCITAAIGCSCKKKVCYRN)	*Carapichea ipecacuanha*	G_s	cAMP accumulation	Antagonist	Single conc. (260 nM) reduced CRF potency by ~4.5-fold	21
**CXCR4**	Senkyunolide I	*Ligusticum chuanxiong*	n.d.	SPR, molecular docking	Antagonist	K_d = 2.94 μM	22
**D2R**	Wilfortrine	*Tripterygium wilfordii*	G_i, β-arrestin	PiggyBac-TANGO, cAMP response	Agonist	EC_{50} = 153.5 nM (cAMP)	23
**D2R**	Atractylon	*Atractylodes macrocephala*	G_i, β-arrestin	PiggyBac-TANGO, cAMP response, *in vivo* activity assays	Agonist	IC_{50} = 2.5 μM (cAMP)	24
**D3R**	Icariside II	*Epimedium koreanum*	$G_{i/o}$	CLBA, cAMP accumulation, Ca^{2+} mobilization, molecular docking, *in vivo* activity assays	Agonist	EC_{50} = 13.29 μM (cAMP)	25
**M_2R**	Icaritin	*Epimedium koreanum*	$G_{i/o}$	CLBA, cAMP accumulation, Ca^{2+} mobilization, molecular docking, *in vivo* activity assays	Antagonist	Single conc. (100 μM) inhibited acetylcholine potency by 54.55%	25
**KOR**	O-methylcoclaurine (1), N-methylcoclaurine (2), coclaurine (3)	*Nelumbo nucifera*	n.d.	CLBA, *in vivo* activity assays	Agonist	K_i = 3.5 μM (1), 0.9 μM (2), 2.2 μM (3), 19.08 μM (4)	26

**DOR, MOR**	Neferine	*Nelumbo nucifera*	n.d.	CLBA, GTPγS binding assay	Agonist	DOR: K_i = 0.7 μM, EC_{50} = 7.9 μM, MOR: K_i = 1.8 μM, EC_{50} > 21 μM	26
**5-HT$_{1D}$R, 5-HT$_{2C}$R**	Hypericin (1), amentoflavone (2)	*Hypericum perforatum*	n.d.	CLBA	Antagonist	5-HT$_{1D}$R: IC_{50} = 4.094 μM (2), 5-HT$_{2C}$R: IC_{50} = 2.555 μM (2)	27
**D1R, D3R, D4R**	Hyperforin (1), amentoflavone (2), hypericin (3), pseudohypericin (4), quercetin (5)	*Hypericum perforatum*	n.d.	CLBA	Antagonist	D1R: IC_{50} = 0.59 μM (1), D3R: IC_{50} = 1.24 μM (2), 0.034 μM (3), 0.62 μM (4) D4R: IC_{50} = 1.47 μM (3), 1.27 μM (4), 7.84 μM (5)	27
**M5R**	Rutin	*Hypericum perforatum*	n.d.	CLBA	Antagonist	IC_{50} = 0.035 μM	27
**CRF1R**	Hypericin	*Hypericum perforatum*	n.d.	CLBA, GTPγS binding assay	Antagonist	IC_{50} = 300 nM	28
**DOR, KOR, MOR**	Hypericin (1), hyperforin (2)	*Hypericum perforatum*	n.d.	CLBA	Antagonist	DOR: IC_{50} = 4 μM (1), 0.5 μM (2), KOR: IC_{50} = 3 μM (1), 1 μM (2), MOR: IC_{50} = 1 μM (1), 0.4 μM (2)	28
**DOR, KOR, MOR, NOR**	Seven natural compounds	*Ganoderma cochlear*, *Carthamus tinctorius*	n.d.	DMR	Antagonist	IC_{50} = 10 - 300 μM	29

**FFAR1 (GPR40)**	Cyanidin-3-O-glucoside (1), delphinidin-3-O-glucoside (2)	*Zea mays*	$G_{q/11}$	IP1 accumulation, insulin secretion	Agonist	EC_{50} = 249 µM (1), 169.7 µM (2)	30
**FFAR1 (GPR40)**	Berberine	*Rhizoma Coptidis*	$G_{q/11}$	Ca^{2+} mobilization *in vivo* activity assays	Agonist	EC_{50} = 0.76 µM (Ca^{2+})	31
**FFAR1 (GPR40), GPR55**	Gintonin (glycolipoprotein complex)	*Panax ginseng*	$G_{q/11}$	Insulin secretion, Ca^{2+} mobilization	Agonist	Dose- and time-dependent stimulation of insulin secretion and Ca^{2+} transients	32
**FFAR1 (GPR40)**	Aloe emodin	*Rheum palmatum*	G_q	Molecular docking, *in vivo* activity assays	Antagonist	Single conc. (10, 20, 40 mg/kg) *in vivo* activity assays	33
**FFAR4 (GPR120)**	α-linolenic acid	*Perilla frutesccns*	G_q	Ca^{2+} imaging, ERK phosphorylation	Agonist	EC_{50} ≈ 0.6 µM (Ca^{2+})	34,35
**FPR2**	Columbamine	*Coptis chinensis*, *Jateorhiza palmata*	G_i, β-arrestin	cAMP response, Ca^{2+} mobilization, β-arrestin recruitment, molecular docking, *in vivo* activity assays	Agonist	EC_{50} ≈ 30 µM (cAMP)	36
**GPBAR1 (TGR5)**	Oleanolic acid	*Olea europaea*	n.d.	Luciferase reporter assay, *in vivo* activity assays	Agonist	EC_{50} = 1.42 µM	37
**GPBAR1 (TGR5)**	Ursolic acid	*Olea europaea*	n.d.	cAMP accumulation, GLP-1 secretion, *in vivo* activity assays	Agonist	EC_{50} = 4.18 µM	38,39
**GPBAR1 (TGR5)**	(+)-vitisinol E (1), paeoninol (2)	*Paeonia lactiflora*	MEK/ERK, IP3, Ca^{2+}/Ca MKII	cAMP accumulation, GLP-1 secretion, molecular docking	Agonists	Single conc. (50, 100 µM) increased cAMP contents with stimulative ratios more than 40%	40

**GPBAR1 (TGR5)**	Tauroursodeoxych-olic acid	Fel Ursi	n.d.	cAMP response, *in vivo* activity assays	Agonist	Single conc. (200 μM) increased cAMP production in time-dependent manner	41,42
**GPBAR1 (TGR5)**	Compound K (metabolite of ginsenosides)	*Panax ginseng*	G_s	Ca^{2+} mobilization, cAMP accumulation, GLP-1 secretion	Agonist	EC_{50} = 244 μM (Ca^{2+}) EC_{50} = 10.6 μM (cAMP)	43
**GPBAR1 (TGR5)**	Aqueous ethanolic extract	*Spergularia marina*	G_s	Ca^{2+} mobilization, cAMP accumulation, GLP-1 secretion	Agonist	EC_{50} = 63.4 μg/mL (cAMP)	44
**GPR30**	Genistein	*Genista tinctoria*, *Sophora subprostrala*	G_s	cAMP accumulation, *in vivo* activity assays	Agonist	Single conc. (2.5, 5 μM) stimulated cAMP production	45
**GPR55**	Curcumin	*Curcuma longa*	$G_{12/13}$	Luciferase reporter assays, Ca^{2+} mobilization, molecular docking	Agonist	Single conc. (10 μM) increased Ca^{2+} levels in a GPR55-dependent manner	46
**GPR97**	Curcumin	*Curcuma longa*	$G_{i/o}$	Luciferase reporter assays	Agonist	Single conc. (10, 20 μM) induced SRF-RE-mediated transcription in a GPR97-dependent manner	47
**GPR108**	Gambogic acid	*Garcinia hanburyi*	n.d.	Luciferase reporter assay, *in vivo* activity assays	Antagonist	n.d.	48,49
**GPRC6A**	Gallic acid (1), epigallocatechin 3-gallate (2)	*Camellia sinensis*	n.d.	ERK phosphorylation, molecular docking	Agonist (1), Antagonist (2)	Single conc. (50, 100 μM) inhibited or stimulated GPRC6A-mediated ERK phosphorylation	50

**KOR**	Salvinorin A	*Salvia divinorum*	$G_{i/o}$	CLBA, GTP turnover, cAMP inhibition	Agonist	K_i = 16 nM (CLBA), EC_{50} = 1 nM (cAMP), EC_{50} = 235 nM (GTPase)	51
**KOR**	Helianorphins-19	*Helianthus annuus*	G_i, β-arrestin	CLBA, cAMP inhibition, β-arrestin recruitment, *in vivo* activity assays	Agonist	K_i = 21 nM (CLBA), EC_{50} = 45 nM (cAMP) EC_{50} = 1.4 μM (β-arrestin)	52
**LPAR**	Gintonin (glycolipoprotein complex)	*Panax ginseng*	$G_{i/o}$, $G_{12/13}$, $G_{q/11}$	Ca^{2+} mobilization, ERK phosphorylation, cAMP response	Agonist	Binding affinity in order of LPA2 > LPA5 > LPA1 > LPA3 > LPA4 receptors	53
**M3R**	Hyoscyamine (1), scopolamine (2)	*Daturae flos*	n.d.	Affinity chromatography, HPLC-MS/MS	n.d.	n.d.	54
**mGluR5**	Monellin	*Dioscoreophy-llum cumminsii*	$G_{q/11}$	CLBA, Ca^{2+} mobilization, IP1 accumulation	Agonist	EC_{50} = 10 μM (Ca^{2+})	55
**MOR**	7-hydroxymitragynine (converted to mitragynine pseudoindoxyl *in vivo*)	*Mitragyna speciosa*	n.d.	CLBA, *in vivo* activity assays	Agonist	K_i = 77.9 nM (CLBA)	56
**MOR**	Ignavine	*Aconiti*	G_i	CLBA, cAMP inhibition, molecular docking, *in vivo* activity assays	Allosteric modulator	IC_{50} = 2 μM (CLBA), Single conc. (1 μM) induced 3.8-fold decrease of EC50 in response to DAMGO	57
**MRGPRX2**	Genistein	*Genista tinctoria*, *Sophora subprostrala*	β-arrestin	Ca^{2+} mobilization, PRESTO-Tango, molecular docking, *in vivo* activity assays	Antagonist	IC_{50} = 3.83 × 10^{-5} M (PRESTO-Tango), IC_{50} = 30 μM (Ca^{2+})	45,58

**MRGPRX2**	Osthole	*Cnidium monnieri*	n.d.	Ca^{2+} mobilization, molecular docking	Antagonist	Inhibited agonist-induced Ca^{2+} mobilization and degranulation in a dose-dependent fashion	59
**MT_1**	Oxyprenylated ferulic acid derivatives, umbelliferone derivatives	*Cassia cinnamon*	n.d.	CLBA	Agonist	K_i = 59.5 µM (4'-Gernayloxyferulic acid), 785.8 nM (Boropinal), 5.2 µM (7-iso-pentenyloxycoumarin), 1.9 µM (Auraotene), 3 nM (Umbellinprenin)	60
**MT_2**	Gastropolybenzyles	*Gastrodia elata*	n.d.	Ca^{2+} mobilization	Agonist	EC_{50} = 76 µM (Ca^{2+})	61
**MT_1, MT_2**	Polybenzyls	*Gastrodia elata*	n.d.	Ca^{2+} mobilization, molecular docking	Agonist	MT_1: EC_{50} = 237 µM, MT_2: EC_{50} = 244 µM	62
**MT_1, MT_2**	Catechin (1), epicatechin (2)	*Uncaria rhynchophylla*	n.d.	Ca^{2+} mobilization, *in vivo* activity assays	Agonist	MT_1: EC_{50} = 26 µM (1), 156 µM (2), MT_2: EC_{50} = 47 µM (1), 209 µM (2)	63
**NTSR**	Cyclopsychotride A	*Psychotria longipes*	n.d.	CBLA, Ca^{2+} mobilization	Agonist	IC_{50} = 3 µM (CBLA)	64
**OX1R, OX2R**	Neferine	*Plumula nelumbinis*	n.d.	SPR, molecular docking	Antagonist	OX1R: K_d = 2 nM (SPR), OX2R: K_d = 10 nM (SPR)	65

**TAS2R1, TAS2R8, TAS2R14**	Ligstroside aglycon (1), oleuropein aglycon (2)	*Olea europaea*	G_{gust}	Ca^{2+} mobilization	Agonist	TAS2R1: EC_{50} = 168.8 μM (1), 141.6 μM (2); TAS2R8: EC_{50} = 57.3 μM (1), 57.3 μM (2); TAS2R14: EC_{50} = 108.3 μM (1)	66,67
**TAS2R10, TAS2R14, TAS2R49**	Bergapten (1), xanthotoxin (2), isopimpinellin (3), skimmianine (4)	*Ruta graveolens*	n.d.	Ca^{2+} imaging	Agonist	TAS2R10: EC_{50} = 2.8 μM (1), 20.6 μM (2), 12 μM (3); TAS2R14: EC_{50} = 10.8 μM (2), 11.1 μM (3), 15.8 μM (4); TAS2R49: EC_{50} = 4 μM (4)	68
**TAS2R10**	Kudinoside A	*Ilex kudingcha*	G_{gust}	Ca^{2+} mobilization	Agonist	Single conc. (30 μM) induced Ca^{2+} release	69
**TAS2R14**	Picrotoxinin	*Ginkgo biloba*	G_{gust}	Ca^{2+} mobilization	Agonist	EC_{50} = 18 μM	70,71
**TAS2R14**	Apigenin (1), chrysin (2)	*Bee propolis* (1), *Scutellaria baicalensis* (2)	G_{gust}	Ca^{2+} mobilization	Agonist	Single conc. (30 μM (1), 120 μM (2)) induced Ca^{2+} release	72
**TAS2R14**	Aristolochic acid	*Aristolochia debilis*	G_i, G_{gust}	Ca^{2+} mobilization, G protein activation, Cryo-EM structure	Agonist	EC_{50} = 10.4 μM (Ca^{2+})	73
**TAS2R38**	Berberine	*Rhizoma coptidis*	n.d.	GLP-1 secretion assay, Ca^{2+} imaging	Agonist	Single conc. (100 μM) caused a rapid increase in Ca^{2+} concentration	74,75

**TAS2R46**	Quinine	*Cinchona calisaya*	G_{gust}	Ca^{2+} imaging	Agonist	EC_{50} = 10 μM	76,77
**TAS2R46**	Strychnine	*Strychnos nu-xvomica*	G_{gust}	Ca^{2+} mobilization, Cryo-EM structure	Agonist	EC_{50} = 0.43 μM	78,79
**TAS2R50**	Andrographolide (1), amarogentin (2)	*Andrographis paniculata*, *Gentiana scabra*	G_{gust}	Ca^{2+} mobilization	Agonist	EC_{50} = 22.9 μM (1), >300 μM (2)	80,81
**V_{1A}R, OTR**	Kalata B7	*Oldenlandia affinis*	$G_{q/11}$	CLBA, IP1 accumulation	Agonist	OTR: K_i = 50 μM, EC_{50} = 12 μM, V_{1A}R: K_i = 12 μM, EC_{50} = 4 μM	82
**α_{1A}-AR, α_{1B}-AR, α_{1D}-AR**	ρ-TIA	*Conus tulipa*	n.d.	CLBA	Antagonist	α_{1A}-AR: IC_{50} = 18 nM, α_{1B}-AR: IC_{50} = 2 nM, α_{1D}-AR: IC_{50} = 25 nM	83
**α_{2C}-AR**	Yohimbine	*Pausinystalia yohimbe*	n.d.	Molecular docking	Antagonist	pK_i = 9.01 μM	84
**β_1AR**	Pulegone	*Ziziphora clinopodioides*	n.d.	A-MS, molecular docking	Antagonist	n.d.	85
**β_2AR**	Ephedrine	*Pinellia ternate*, Qingfei Xiaoyan Wan	n.d.	UPLC/Q-TOF-MS, luciferase reporter assays	Agonist	Single conc. (100 μM) activated the receptor	86,87
**β_2AR**	Ephedrine	Chuanbeipipa dropping pills	n.d.	UPLC/Q-TOF-MS, luciferase reporter assay	Agonist	Single conc. (10 μM) activated the receptor	88

Note: The abbreviation of the targets refers to the GPCR database (https://gpcrdb.org/drugs/drugbrowser); **CLBA**, competitive ligand-binding assay;

SLCA, split luciferase complementation assay; **SPR**, surface plasmon resonance; **A-MS**, affinity mass spectrometry; **DMR**, Dynamic mass redistribution; **UPLC/Q-TOF-MS**, ultra-high performance liquid chromatography with quadrupole time-of-flight mass spectrometry; **HPLC-MS/MS**, liquid chromatography (HPLC)–mass spectrometry (MS); **IP1,** inositol monophosphate; **n.d.**, not detected; **Single conc.**, single concentration; **GTP**, guanosine triphosphate; **GLP-1**, glucagon-like peptide-1; **SRF-RE**, serum response factor-response element.

Table S2. Indirect effects of traditional medicines on the GPCR function over the last two decades

Effects	Molecules	Natural origin	Biological models	Detection methods	Potential pharmacological activity	References
Promoting GLP-1 secretion	*Gentiana scabra* root extract	*Gentiana scabra*	NCI-H716 cell, Lepr $^{-/-}$ (db/db) mice	GLP-1 releasing, Ca^{2+} imaging	Lowering blood glucose level	89
Enhancing the efficacy of melatonin	Ex18	*Pistacia vera*	HEK293 cell	CLBA, ERK activation, β-arrestin recruitment	Melatonin-potentiating activity	90
Activating Ca^{2+}/MAPK signaling pathway	α-Hederin	*Akebia trifoliata*	CRC cell, Nude male mice	Ca^{2+}mobilization, molecular docking, RT-qPCR	Inducing non-apoptotic cell death and paraptosis	91
Enhancing GLP-1 potency	N55	*Trigonella foenumgraecum*	U2OS, RINm5F, H460 cell	CLBA, cAMP accumulation, receptor endocytosis	Reducing glucose and glycated hemoglobin levels	92
Suppressing the activation of β-arrestin 1	Polysaccharides	*Dendrobium Officinaleon*	NCM460 cell, BalB/c mice	β-arrestin 1 expression, anti-inflammatory activity	Potential therapeutic effect on ulcerative colitis	93
Mediating β-arrestin 2 signaling	Total flavone	*Pollen Typhae*	C2C12 myoblast	Glucose uptake, β-arrestin 2 expression	Enhancing insulin-stimulated glucose uptake	94

Promoting GLP-1 secretion	Curcumin	*Curcuma longa*	GLUTag cell	Glucose tolerance, GLP-1 secretion	Improving glucose tolerance	95
Activate the cAMP pathway by binding to GNAS	Schizandrin A, formononetin, schisantherin A, notoginsenoside Ft1, gallic acid, ginsenoside F2, catalpol	Shenqi Jiangtang Granules	Glomerular mesangial cells	Bio-layer interferometry, UHPLC-Q/TOF-MS/MS, cAMP assay, Ca^{2+}-Mg^{2+}-ATPase level assay	Nephroprotective effect	96

Note: The abbreviation of the receptors refers to the GPCR database (https://gpcrdb.org/drugs/drugbrowser). **CLBA**, competitive ligand-binding assay; **GLP-1**, glucagon-like peptide-1; **MAPK,** Mitogen-activated protein kinase; **RT-qPCR**, quantitative real-time PCR; **UHPLC-Q/TOF-MS/MS**, ultra-high performance liquid chromatography with quadrupole time-of-flight mass spectrometry.

Table S3. FDA-approved nature-derived drugs targeting GPCRs

Drug	Target	Action	Natural origin	FDA approval year
**Cannabidiol**	Cannabinoid receptor 1	Antagonism	*Cannabis sativa*	2018
**Vorapaxar**	Protease-activated receptor 1	Antagonism	*Galbulimima baccata* (Derivative of himbacine)	2014
**Codeine**	Opioid receptors	Agonism	*Papaver somniferum*	2009
**Exenatide**	Glucagon-like peptide-1 receptor	Agonism	*Heloderma suspectum*	2005
**Caffeine**	Adenosine receptors	Antagonism	*Coffea arabica*	1999
**Dronabinol**	Cannabinoid receptors	Agonism	*Cannabis sativa*	1985
**Scopolamine**	Muscarinic acetylcholine receptors	Antagonism	*Datura stramonium*	1979
**Theophylline**	Adenosine receptors	Antagonism	*Camellia sinensis*	1976
**Pseudoephedrine**	Adrenergic receptors	Agonism	*Ephedra sinica*	1975
**Pilocarpine**	Muscarinic acetylcholine receptors	Agonism	*Pilocarpus microphyllus*	1974
**Atropine**	Muscarinic acetylcholine receptors	Antagonism	*Atropa belladonna*	1973
**Ephedrine**	Adrenergic receptors	Agonism	*Ephedra sinica*	1963
**Ergotamine**	Serotonin receptors; Alpha-1 adrenergic receptors	Agonism	*Claviceps purpurea*	1960

**Ergometrine**	Alpha-1A adrenergic receptor; Dopamine receptors; Serotonin receptors	Agonism	*Claviceps purpurea*	1946
**Hydrocodone**	Mu and delta opioid receptors	Agonism	*Papaver somniferum* (Derivative of Codeine)	1943
**Morphine**	Mu, delta and kappa opioid receptors	Agonism	*Papaver somniferum*	1941

Note: The listed drugs are from the Drugs@FDA database. (https://www.accessdata.fda.gov/scripts/cder/daf/index.cfm), and cross-referenced in the Drugbank (https://go.drugbank.com/), pharmacodia (https://www.pharmacodia.com/homeH5.html), and pharnexcloud database (https://www.pharnexcloud.com/). The abbreviation of the targets refers to the GPCR database (https://gpcrdb.org/drugs/drugbrowser).

References

1. Gafner S., Dietz B. M., McPhail K. L., et al. (2006). Alkaloids from *Eschscholzia californica* and Their Capacity to Inhibit Binding of [^{3}H]8-Hydroxy-2-(di-*N*-propylamino)tetralin to 5-HT_{1A} Receptors in Vitro. *J. Nat. Prod.* **69**:432-435. DOI:10.1021/np058114h
2. Nishi A., Yamaguchi T., Sekiguchi K., et al. (2012). Geissoschizine methyl ether, an alkaloid in Uncaria hook, is a potent serotonin1A receptor agonist and candidate for amelioration of aggressiveness and sociality by yokukansan. *Neuroscience* **207**:124-136. DOI:10.1016/j.neuroscience.2012.01.037
3. Madsen M. K., Fisher P. M., Burmester D., et al. (2019). Psychedelic effects of psilocybin correlate with serotonin 2A receptor occupancy and plasma psilocin levels. *Neuropsychopharmacol.* **44**:1328-1334. DOI:10.1038/s41386-019-0324-9
4. Dodd S., Norman T. R., Eyre H. A., et al. (2023). Psilocybin in neuropsychiatry: a review of its pharmacology, safety, and efficacy. *CNS Spectr.* **28**:416-426. DOI:10.1017/S1092852922000888
5. Zhang B., Zhao S., Yang D., et al. (2020). A Novel G Protein-Biased and Subtype-Selective Agonist for a G Protein-Coupled Receptor Discovered from Screening Herbal Extracts. *ACS Cent. Sci.* **6**:213-225. DOI:10.1021/acscentsci.9b01125
6. Dietz B. M., Mahady G. B., Pauli G. F., et al. (2005). Valerian extract and valerenic acid are partial agonists of the 5-HT_{5a} receptor in vitro. *Molecular Brain Research* **138**:191-197. DOI:10.1016/j.molbrainres.2005.04.009
7. Lacher S. K., Mayer R., Sichardt K., et al. (2007). Interaction of valerian extracts of different polarity with adenosine receptors: Identification of isovaltrate as an inverse agonist at A1 receptors. *Biochemical Pharmacology* **73**:248-258. DOI:10.1016/j.bcp.2006.09.029
8. Schumacher B., Scholle S., Hölzl J., et al. (2002). Lignans Isolated from Valerian: Identification and Characterization of a New Olivil Derivative with Partial Agonistic Activity at A1 Adenosine Receptors. *J. Nat. Prod.* **65**:1479-1485. DOI:10.1021/np010464q
9. Rukoyatkina N., Shpakova V., Bogoutdinova A., et al. (2022). Curcumin by activation of adenosine A_{2A} receptor stimulates protein kinase a and potentiates inhibitory effect of cangrelor on platelets. *Biochemical and Biophysical Research Communications* **586**:20-26. DOI:10.1016/j.bbrc.2021.11.006
10. Portilla-Martínez A., Ortiz-Flores M. Á., Meaney E., et al. (2022). (-)-Epicatechin Is a Biased Ligand of Apelin Receptor. *International Journal of Molecular Sciences* **23**:8962. DOI:10.3390/ijms23168962
11. Punyasiri P. A. N., Abeysinghe I. S. B., Kumar V., et al. (2004). Flavonoid biosynthesis in the tea plant *Camellia sinensis*: properties of enzymes of the prominent epicatechin and catechin pathways. *Archives of Biochemistry and Biophysics* **431**:22-30. DOI:10.1016/j.abb.2004.08.003
12. Zhu Y., Wu L., Zhao Y., et al. (2022). Discovery of oridonin as a novel agonist for BRS-3. *Phytomedicine* **100**:154085.

DOI:10.1016/j.phymed.2022.154085

13. Wei T.-T., Chandy M., Nishiga M., et al. (2022). Cannabinoid receptor 1 antagonist genistein attenuates marijuana-induced vascular inflammation. *Cell* **185**:1676-1693.e1623. DOI:10.1016/j.cell.2022.04.005

14. Skalicky M., Kubes J., Hejnak V., et al. (2018). Isoflavones Production and Possible Mechanism of Their Exudation in *Genista tinctoria* L. Suspension Culture after Treatment with Vanadium Compounds. *Molecules* **23**:1619. DOI:10.3390/molecules23071619

15. Ye B., Chen P., Lin C., et al. (2023). Study on the material basis and action mechanisms of *sophora davidii* (Franch.) skeels flower extract in the treatment of non-small cell lung cancer. *Journal of Ethnopharmacology* **317**:116815. DOI:10.1016/j.jep.2023.116815

16. LaVigne J. E., Hecksel R., Keresztes A., et al. (2021). Cannabis sativa terpenes are cannabimimetic and selectively enhance cannabinoid activity. *Scientific Reports* **11**:8232. DOI:10.1038/s41598-021-87740-8

17. Pandey P., Kumarihamy M., Chaturvedi K., et al. (2023). In Vitro and In Silico Studies of Neolignans from *Magnolia grandiflora* L. Seeds against Human Cannabinoids and Opioid Receptors. *Molecules* **28**:1253. DOI:10.3390/molecules28031253

18. Jiang X., Chen S., Zhang Q., et al. (2020). Celastrol is a novel selective agonist of cannabinoid receptor 2 with anti-inflammatory and anti-fibrotic activity in a mouse model of systemic sclerosis. *Phytomedicine* **67**:153160. DOI:10.1016/j.phymed.2019.153160

19. Chen S.-R., Dai Y., Zhao J., et al. (2018). A Mechanistic Overview of Triptolide and Celastrol, Natural Products from Tripterygium wilfordii Hook F. *Frontiers in Pharmacology* **9**. DOI:10.3389/fphar.2018.00104

20. Taghizadeh M. S., Retzl B., Muratspahić E., et al. (2022). Discovery of the cyclotide caripe 11 as a ligand of the cholecystokinin-2 receptor. *Scientific Reports* **12**:9215. DOI:10.1038/s41598-022-13142-z

21. Fahradpour M., Keov P., Tognola C., et al. (2017). Cyclotides Isolated from an Ipecac Root Extract Antagonize the Corticotropin Releasing Factor Type 1 Receptor. *Frontiers in Pharmacology* **8**:616. DOI:10.3389/fphar.2017.00616

22. Chen L., Lv D., Wang S., et al. (2020). Surface Plasmon Resonance-Based Membrane Protein-Targeted Active Ingredients Recognition Strategy: Construction and Implementation in Ligand Screening from Herbal Medicines. *Anal. Chem.* **92**:3972-3980. DOI:10.1021/acs.analchem.9b05479

23. Li F., Jiang X., Luo L.-L., et al. (2019). A piggyBac-based TANGO GFP assay for high throughput screening of GPCR ligands in live cells. *Cell Commun Signal* **17**:49. DOI:10.1186/s12964-019-0359-x

24. Li H., Wang F., Zhou Z., et al. (2022). Atractylon, a novel dopamine 2 receptor agonist, ameliorates Parkinsonian like motor dysfunctions in MPTP-induced mice. *NeuroToxicology* **89**:121-126. DOI:10.1016/j.neuro.2022.01.010

25. Seong S. H., Kim S. H., Ryu J. H., et al. (2023). Effects of Icariin and Its Metabolites on GPCR Regulation and MK-801-Induced Schizophrenia-Like Behaviors in Mice. *Molecules* **28**:7300. DOI:10.3390/molecules28217300

26. Kumarihamy M., León F., Pettaway S., et al. (2015). In vitro opioid receptor affinity and in vivo behavioral studies of *Nelumbo nucifera* flower. *Journal of Ethnopharmacology* **174**:57-65. DOI:10.1016/j.jep.2015.08.006

27. Butterweck V., Nahrstedt A., Evans J., et al. (2002). In vitro receptor screening of pure constituents of St. John's wort reveals novel interactions with a number of GPCRs. *Psychopharmacology* **162**:193-202. DOI:10.1007/s00213-002-1073-7

28. Simmen U., Higelin J., Berger-Büter K., et al. (2001). Neurochemical Studies with St. John's WortInVitro. *Pharmacopsychiatry* **34**:137-142. DOI:10.1055/s-2001-15475

29. Hou T., Xu F., Peng X., et al. (2021). Label-free cell phenotypic study of opioid receptors and discovery of novel mu opioid ligands from natural products. *Journal of Ethnopharmacology* **270**:113872. DOI:10.1016/j.jep.2021.113872

30. Luna-Vital D. A. and Gonzalez de Mejia E. (2018). Anthocyanins from purple corn activate free fatty acid-receptor 1 and glucokinase enhancing in vitro insulin secretion and hepatic glucose uptake. *PLOS One* **13**:e0200449. DOI:10.1371/journal.pone.0200449

31. Rayasam G. V., Tulasi V. K., Sundaram S., et al. (2010). Identification of berberine as a novel agonist of fatty acid receptor GPR40. *Phytotherapy Research* **24**:1260-1263. DOI:10.1002/ptr.3165

32. Cho Y.-J., Choi S.-H., Lee R., et al. (2020). Ginseng Gintonin Contains Ligands for GPR40 and GPR55. *Molecules* **25**:1102. DOI:10.3390/molecules25051102

33. Bao W., Lyu J., Feng G., et al. (2024). Aloe emodin promotes mucosal healing by modifying the differentiation fate of enteroendocrine cells via regulating cellular free fatty acid sensitivity. *Acta Pharmaceutica Sinica B*. DOI:10.1016/j.apsb.2024.05.027

34. Hirasawa A., Tsumaya K., Awaji T., et al. (2005). Free fatty acids regulate gut incretin glucagon-like peptide-1 secretion through GPR120. *Nat Med* **11**:90-94. DOI:10.1038/nm1168

35. Kim J. T., Lee S. B., Son M. J., et al. (2023). Perilla oil and α-linolenic acid ameliorated thrombosis in rats induced by collagen and epinephrine. *Food Sci Biotechnol* **32**:997-1003. DOI:10.1007/s10068-022-01241-6

36. Wu M. Y., Ge Y. J., Wang E. J., et al. (2023). Enhancement of efferocytosis through biased *FPR2* signaling attenuates intestinal inflammation. *EMBO Mol Med* **15**:e17815. DOI:10.15252/emmm.202317815

37. Sato H., Genet C., Strehle A., et al. (2007). Anti-hyperglycemic activity of a TGR5 agonist isolated from Olea europaea. *Biochemical and Biophysical*

Research Communications **362**:793-798. DOI:10.1016/j.bbrc.2007.06.130

38. Lo S.-H., Li Y., Cheng K. C., et al. (2017). Ursolic acid activates the TGR5 receptor to enhance GLP-1 secretion in type 1-like diabetic rats. *Naunyn-Schmiedeberg's Arch Pharmacol* **390**:1097-1104. DOI:10.1007/s00210-017-1409-9
39. Somova L. I., Shode F. O. and Mipando M. (2004). Cardiotonic and antidysrhythmic effects of oleanolic and ursolic acids, methyl maslinate and uvaol. *Phytomedicine* **11**:121-129. DOI:10.1078/0944-7113-00329
40. Wu S.-L., Zhang C.-C., Chen J.-J., et al. (2022). Oligostilbenes from the seeds of Paeonia lactiflora as potent GLP-1 secretagogues targeting TGR5 receptor. *Fitoterapia* **163**:105336. DOI:10.1016/j.fitote.2022.105336
41. Yanguas-Casás N., Barreda-Manso M. A., Nieto-Sampedro M., et al. (2017). TUDCA: An Agonist of the Bile Acid Receptor GPBAR1/TGR5 With Anti-Inflammatory Effects in Microglial Cells. *Journal Cellular Physiology* **232**:2231-2245. DOI:10.1002/jcp.25742
42. Wang X., Wu H., Li M., et al. (2024). A Comprehensive Analysis of Fel Ursi and Its Common Adulterants Based on UHPLC-QTOF-MSE and Chemometrics. *Molecules* **29**:3144. DOI:10.3390/molecules29133144
43. Kim K., Park M., Lee Y. M., et al. (2014). Ginsenoside metabolite compound K stimulates glucagon-like peptide-1 secretion in NCI-H716 cells via bile acid receptor activation. *Arch. Pharm. Res.* **37**:1193-1200. DOI:10.1007/s12272-014-0362-0
44. Kim K., Lee Y. M., Rhyu M.-R., et al. (2014). *Spergularia marina* Induces Glucagon-Like Peptide-1 Secretion in NCI-H716 Cells Through Bile Acid Receptor Activation. *Journal of Medicinal Food* **17**:1197-1203. DOI:10.1089/jmf.2013.3091
45. Luo J., Wang A., Zhen W., et al. (2018). Phytonutrient genistein is a survival factor for pancreatic β-cells via GPR30-mediated mechanism. *The Journal of Nutritional Biochemistry* **58**:59-70. DOI:10.1016/j.jnutbio.2018.04.018
46. Harada N., Okuyama M., Teraoka Y., et al. (2022). Identification of G protein-coupled receptor 55 (GPR55) as a target of curcumin. *npj Sci Food* **6**:4. DOI:10.1038/s41538-021-00119-x
47. Harada N., Arahori Y., Okuyama M., et al. (2022). Curcumin activates G protein-coupled receptor 97 (GPR97) in a manner different from glucocorticoid. *Biochemical and Biophysical Research Communications* **595**:41-46. DOI:10.1016/j.bbrc.2022.01.075
48. Lyu S., Zhang X., Tu Z., et al. (2022). GPR108 is required for gambogic acid inhibiting NF-κB signaling in cancer. *Pharmacological Research* **182**:106279. DOI:10.1016/j.phrs.2022.106279
49. Liu Y., Chen Y., Lin L., et al. (2020). Gambogic Acid as a Candidate for Cancer Therapy: A Review. *IJN* **Volume 15**:10385-10399. DOI:10.2147/IJN.S277645

50. Pi M., Kapoor K., Ye R., et al. (2018). GPCR6A Is a Molecular Target for the Natural Products Gallate and EGCG in Green Tea. *Molecular Nutrition Food Res* **62**:1700770. DOI:10.1002/mnfr.201700770

51. Roth B. L., Baner K., Westkaemper R., et al. (2002). Salvinorin A: A potent naturally occurring nonnitrogenous κ opioid selective agonist. *Proceedings of the National Academy of Sciences* **99**:11934-11939. DOI:10.1073/pnas.182234399

52. Muratspahić E., Tomašević N., Koehbach J., et al. (2021). Design of a Stable Cyclic Peptide Analgesic Derived from Sunflower Seeds that Targets the κ-Opioid Receptor for the Treatment of Chronic Abdominal Pain. *Journal of Medicinal Chemistry* **64**:9042-9055. DOI:10.1021/acs.jmedchem.1c00158

53. Hwang S. H., Shin T.-J., Choi S.-H., et al. (2012). Gintonin, Newly Identified Compounds from Ginseng, Is Novel Lysophosphatidic Acids-Protein Complexes and Activates G Protein-Coupled Lysophosphatidic Acid Receptors with High Affinity. *Molecules and Cells* **33**:151-162. DOI:10.1007/S10059-012-2216-z

54. Fan H., Huang X., Zhang Z., et al. (2023). Immobilization of M3 Muscarinic Receptor to Rapidly Analyze Drug—Protein Interactions and Bioactive Components in a Natural Plant. *International Journal of Molecular Sciences* **24**:7171. DOI:10.3390/ijms24087171

55. Chen A. N. Y., Hellyer S. D., Trinh P. N. H., et al. (2020). Identification of monellin as the first naturally derived proteinaceous allosteric agonist of metabotropic glutamate receptor 5. *Basic Clin Pharma Tox* **126**:104-115. DOI:10.1111/bcpt.13239

56. Obeng S., Leon F., Patel A., et al. (2022). Interactive Effects of μ-Opioid and Adrenergic-α_2 Receptor Agonists in Rats: Pharmacological Investigation of the Primary Kratom Alkaloid Mitragynine and Its Metabolite 7-Hydroxymitragynine. *J Pharmacol Exp Ther* **383**:182-198. DOI:10.1124/jpet.122.001192

57. Ohbuchi K., Miyagi C., Suzuki Y., et al. (2016). Ignavine: a novel allosteric modulator of the μ opioid receptor. *Scientific Reports* **6**:31748. DOI:10.1038/srep31748

58. Kumar M., Singh K., Duraisamy K., et al. (2020). Protective Effect of Genistein against Compound 48/80 Induced Anaphylactoid Shock via Inhibiting MAS Related G Protein-Coupled Receptor X2 (MRGPRX2). *Molecules* **25**:1028. DOI:10.3390/molecules25051028

59. Callahan B. N., Kammala A. K., Syed M., et al. (2020). Osthole, a Natural Plant Derivative Inhibits MRGPRX2 Induced Mast Cell Responses. *Frontiers in Immunology* **11**:703. DOI:10.3389/fimmu.2020.00703

60. Hasan M., Genovese S., Fiorito S., et al. (2017). Oxyprenylated Phenylpropanoids Bind to MT1 Melatonin Receptors and Inhibit Breast Cancer Cell Proliferation and Migration. *J. Nat. Prod.* **80**:3324-3329. DOI:10.1021/acs.jnatprod.7b00853

61. Chen S.-Y., Geng C.-A., Ma Y.-B., et al. (2019). Melatonin Receptors Agonistic Activities of Phenols from Gastrodia elata. *Nat. Prod. Bioprospect.* **9**:297-302. DOI:10.1007/s13659-019-0213-2

62. Chen S.-Y., Geng C.-A., Ma Y.-B., et al. (2019). Polybenzyls from Gastrodia elata, their agonistic effects on melatonin receptors and structure-activity relationships. *Bioorganic & Medicinal Chemistry* **27**:3299-3306. DOI:10.1016/j.bmc.2019.06.008

63. Geng C.-A., Yang T.-H., Huang X.-Y., et al. (2019). Antidepressant potential of Uncaria rhynchophylla and its active flavanol, catechin, targeting melatonin receptors. *Journal of Ethnopharmacology* **232**:39-46. DOI:10.1016/j.jep.2018.12.013

64. Witherup K. M., Bogusky M. J., Anderson P. S., et al. (1994). Cyclopsychotride A, a Biologically Active, 31-Residue Cyclic Peptide Isolated from Psychotria longipes. *J. Nat. Prod.* **57**:1619-1625. DOI:10.1021/np50114a002

65. He J., Fang J., Wang Y., et al. (2023). Discovery of Small-Molecule Antagonists of Orexin 1/2 Receptors from Traditional Chinese Medicinal Plants with a Hypnotic Effect. *Pharmaceuticals* **16**:542. DOI:10.3390/ph16040542

66. Cui M., Chen B., Xu K., et al. (2021). Activation of specific bitter taste receptors by olive oil phenolics and secoiridoids. *Scientific Reports* **11**:22340. DOI:10.1038/s41598-021-01752-y

67. Baccouri B., Rajhi I., Theresa S., et al. (2022). The potential of wild olive leaves (*Olea europaea* L. subsp. oleaster) addition as a functional additive in olive oil production: the effects on bioactive and nutraceutical compounds using LC–ESI–QTOF/MS. *Eur Food Res Technol* **248**:2809-2823. DOI:10.1007/s00217-022-04091-y

68. Mancuso G., Borgonovo G., Scaglioni L., et al. (2015). Phytochemicals from Ruta graveolens Activate TAS2R Bitter Taste Receptors and TRP Channels Involved in Gustation and Nociception. *Molecules* **20**:18907-18922. DOI:10.3390/molecules201018907

69. Zhou Y.-W., Sun J., Wang Y., et al. (2022). Tas2R activation relaxes airway smooth muscle by release of $G\alpha_t$ targeting on AChR signaling. *Proceedings of the National Academy of Sciences* **119**:e2121513119. DOI:10.1073/pnas.2121513119

70. Behrens M., Brockhoff A., Kuhn C., et al. (2004). The human taste receptor hTAS2R14 responds to a variety of different bitter compounds. *Biochemical and Biophysical Research Communications* **319**:479-485. DOI:10.1016/j.bbrc.2004.05.019

71. Ng C. C., Duke R. K., Hinton T., et al. (2017). Effects of bilobalide, ginkgolide B and picrotoxinin on $GABA_A$ receptor modulation by structurally diverse positive modulators. *European Journal of Pharmacology* **806**:83-90. DOI:10.1016/j.ejphar.2017.04.019

72. Hariri B. M., McMahon D. B., Chen B., et al. (2017). Flavones modulate respiratory epithelial innate immunity: Anti-inflammatory effects and activation of the T2R14 receptor. *Journal of Biological Chemistry* **292**:8484-8497. DOI:10.1074/jbc.M116.771949

73. Hu X., Ao W., Gao M., et al. (2024). Bitter taste TAS2R14 activation by intracellular tastants and cholesterol. *Nature* **631**:459-466. DOI:10.1038/s41586-024-07569-9

74. Yu Y., Hao G., Zhang Q., et al. (2015). Berberine induces GLP-1 secretion through activation of bitter taste receptor pathways. *Biochemical Pharmacology* **97**:173-177. DOI:10.1016/j.bcp.2015.07.012

75. Yang W.-L., Zhang C.-Y., Ji W.-Y., et al. (2024). Berberine Metabolites Stimulate GLP-1 Secretion by Alleviating Oxidative Stress and Mitochondrial Dysfunction. *Am. J. Chin. Med.* **52**:253-274. DOI:10.1142/S0192415X24500113

76. Grassin-Delyle S., Abrial C., Fayad-Kobeissi S., et al. (2013). The expression and relaxant effect of bitter taste receptors in human bronchi. *Respiratory Research* **14**:134. DOI:10.1186/1465-9921-14-134

77. do Amaral V. L. L., Frajbat M., Petreanu M., et al. (2014). Reproductive toxicology and clastogenic evaluation in mice of a phytotherapeutic formulation obtained from *Cinchona calisaya* Weddel (Rubiaceae) used in Brazilian folk medicine as female fertility stimulant. *Journal of Ethnopharmacology* **155**:1508-1512. DOI:10.1016/j.jep.2014.07.038

78. Xu W., Wu L., Liu S., et al. (2022). Structural basis for strychnine activation of human bitter taste receptor TAS2R46. *Science* **377**:1298-1304. DOI:10.1126/science.abo1633

79. Brockhoff A., Behrens M., Massarotti A., et al. (2007). Broad Tuning of the Human Bitter Taste Receptor hTAS2R46 to Various Sesquiterpene Lactones, Clerodane and Labdane Diterpenoids, Strychnine, and Denatonium. *J. Agric. Food Chem.* **55**:6236-6243. DOI:10.1021/jf070503p

80. Behrens M., Brockhoff A., Batram C., et al. (2009). The Human Bitter Taste Receptor hTAS2R50 Is Activated by the Two Natural Bitter Terpenoids Andrographolide and Amarogentin. *J. Agric. Food Chem.* **57**:9860-9866. DOI:10.1021/jf9014334

81. Sheeja K., Guruvayoorappan C. and Kuttan G. (2007). Antiangiogenic activity of Andrographis paniculata extract and andrographolide. *International Immunopharmacology* **7**:211-221. DOI:10.1016/j.intimp.2006.10.002

82. Koehbach J., O'Brien M., Muttenthaler M., et al. (2013). Oxytocic plant cyclotides as templates for peptide G protein-coupled receptor ligand design. *Proceedings of the National Academy of Sciences* **110**:21183-21188. DOI:10.1073/pnas.1311183110

83. Chen Z., Rogge G., Hague C., et al. (2004). Subtype-selective Noncompetitive or Competitive Inhibition of Human α1-Adrenergic Receptors by ρ-TIA. *Journal of Biological Chemistry* **279**:35326-35333. DOI:10.1074/jbc.M403703200

84. Hai-Bo L., Yong P., Lu-qi H., et al. (2013). Mechanism of Selective Inhibition of Yohimbine and Its Derivatives in Adrenoceptor *α*2 Subtypes. *Journal of Chemistry* **2013**:1-9. DOI:10.1155/2013/783058

85. Shayiranbieke A., Liang Q., Wang T., et al. (2022). Development of immobilized beta1-adrenoceptor chromatography for rapid discovery of ligands specifically binding to the receptor from herbal extract. *Journal of Chromatography A* **1677**:463298. DOI:10.1016/j.chroma.2022.463298

86. Dong L., Cheng B., Luo Y., et al. (2014). Identification of Nuclear Factor-κB Inhibitors and β_2 Adrenergic Receptor Agonists in Chinese Medicinal Preparation Fuzilizhong Pills Using UPLC with Quadrupole Time-of-flight MS. *Phytochemical Analysis* **25**:113-121. DOI:10.1002/pca.2474

87. Cheng B., Hou Y., Wang L., et al. (2012). Dual-bioactivity-based liquid chromatography-coupled quadrupole time-of-flight mass spectrometry for NF-κB inhibitors and β_2AR agonists identification in Chinese Medicinal Preparation Qingfei Xiaoyan Wan. *Analytical and Bioanalytical Chemistry* **404**:2445-2452. DOI:10.1007/s00216-012-6332-9

88. Dong L., Luo Y., Cheng B., et al. (2013). Bioactivity-integrated ultra-performance liquid chromatography/quadrupole time-of-flight mass spectrometry for the identification of nuclear factor-κB inhibitors and β_2 adrenergic receptor agonists in Chinese medicinal preparation Chuanbeipipa dropping pills. *Biomedical Chromatography* **27**:960-967. DOI:10.1002/bmc.2886

89. Suh H.-W., Lee K.-B., Kim K.-S., et al. (2015). A bitter herbal medicine Gentiana scabra root extract stimulates glucagon-like peptide-1 secretion and regulates blood glucose in db/db mouse. *Journal of Ethnopharmacology* **172**:219-226. DOI:10.1016/j.jep.2015.06.042

90. Labani N., Gbahou F., Noblet M., et al. (2023). Pistacia vera Extract Potentiates the Effect of Melatonin on Human Melatonin MT1 and MT2 Receptors with Functional Selectivity. *Pharmaceutics* **15**:1845. DOI:10.3390/pharmaceutics15071845

91. Rao X., Li Z., Zhang Q., et al. (2024). α-Hederin induces paraptosis by targeting GPCRs to activate Ca^{2+}/MAPK signaling pathway in colorectal cancer. *Cancer Medicine* **13**:e7202. DOI:10.1002/cam4.7202

92. King K., Lin N.-P., Cheng Y.-H., et al. (2015). Isolation of Positive Modulator of Glucagon-like Peptide-1 Signaling from *Trigonella foenum-graecum* (Fenugreek) Seed. *Journal of Biological Chemistry* **290**:26235-26248. DOI:10.1074/jbc.M115.672097

93. Liang J., Chen S., Chen J., et al. (2018). Therapeutic roles of polysaccharides from *Dendrobium Officinaleon* colitis and its underlying mechanisms. *Carbohydrate Polymers* **185**:159-168. DOI:10.1016/j.carbpol.2018.01.013

94. Feng X.-T., Wang T.-Z., Chen Y. I., et al. (2012). Pollen Typhae total flavone improves insulin-induced glucose uptake through the β-arrestin-2-mediated signaling in C2C12 myotubes. *International Journal of Molecular Medicine* **30**:914-922. DOI:10.3892/ijmm.2012.1061

95. Kato M., Nishikawa S., Ikehata A., et al. (2017). Curcumin improves glucose tolerance via stimulation of glucagon-like peptide-1 secretion. *Molecular Nutrition Food Res* **61**:1600471. DOI:10.1002/mnfr.201600471

96. Zhang H., Yao J., Xiao G., et al. (2024). Discovery of drug targets based on traditional Chinese medicine microspheres (TCM-MPs) fishing strategy combined with bio-layer interferometry (BLI) technology. *Analytica Chimica Acta* **1305**:342542. DOI:10.1016/j.aca.2024.342542
