# Supplementary material for: Emerging paradigms for target discovery of traditional medicines: A genome-wide pan-GPCR perspective
Source: Innovation (Camb). 2025 Jan 17;6(3):100774. doi: 10.1016/j.xinn.2024.100774 (PMC11910885; doi:10.1016/j.xinn.2024.100774)
Supplement: Document S2. Article plus supplemental information [file mmc2.pdf]

REVIEW

OPEN ACCESS

Emerging paradigms for target discovery of traditional medicines: A genome-wide pan-GPCR perspective

Zenghao Bi,[1,2,3,6] Huan Li,[2,3,4,6] Yuting Liang,[1,2,3,6] Dan Sun,[2,3] Songxin Liu,[2,3,5] Wei Chen,[2,3] Liang Leng,[2,3] Chi Song,[2,3] Sanyin Zhang,[2,3] Zhaotong Cong,[2,3,*] and Shilin Chen[2,3]

*Correspondence: congzt@cdutcm.edu.cn

Received: August 13, 2024; Accepted: December 22, 2024; Published Online: January 2, 2025; https://doi.org/10.1016/j.xinn.2024.100774

© 2024 The Author(s). Published by Elsevier Inc. on behalf of Youth Innovation Co., Ltd. This is an open access article under the CC BY license (http://creativecommons.org/licenses/by/4.0/).

GRAPHICAL ABSTRACT

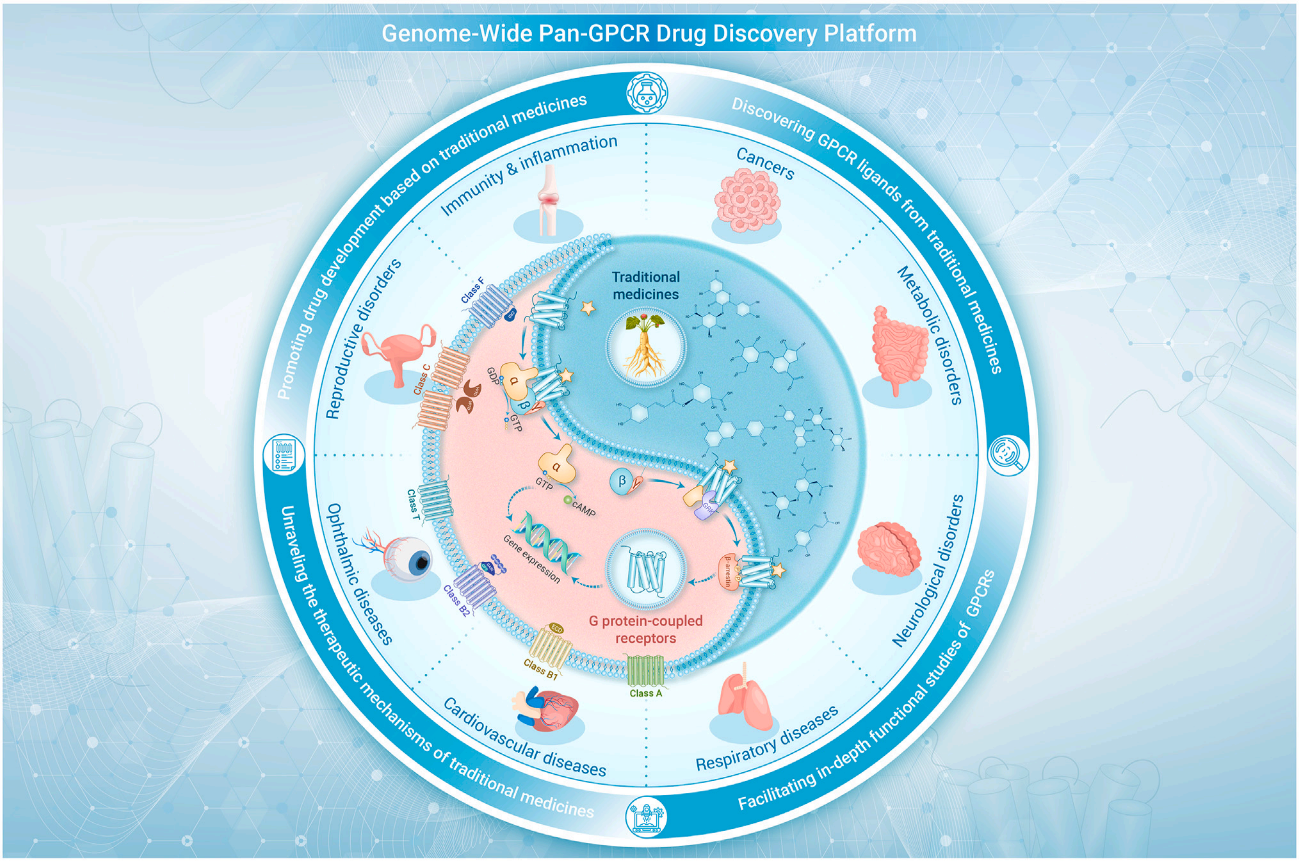

PUBLIC SUMMARY

- Traditional medicines may provide new therapeutic options for difficult-to-treat conditions.
- Therapeutic effects of traditional medicines relate to the modulation of GPCRs.
- Genome-wide pan-GPCR platform aims to explore all-sided relations between traditional medicines and GPCRome.
- Screening against the GPCRome may unravel the multi-component/multi-target properties of traditional medicines.

Emerging paradigms for target discovery of traditional medicines: A genome-wide pan-GPCR perspective

Zenghao Bi,[1,2,3,6] Huan Li,[2,3,4,6] Yuting Liang,[1,2,3,6] Dan Sun,[2,3] Songxin Liu,[2,3,5] Wei Chen,[2,3] Liang Leng,[2,3] Chi Song,[2,3] Sanyin Zhang,[2,3] Zhaotong Cong,[2,3,*] and Shilin Chen[2,3]

[1]School of Pharmacy, Chengdu University of Traditional Chinese Medicine, Chengdu 611137, China
[2]Institute of Herbgenomics, Chengdu University of Traditional Chinese Medicine, Chengdu 611137, China
[3]Innovative Institute of Chinese Medicine and Pharmacy, Chengdu University of Traditional Chinese Medicine, Chengdu 611137, China
[4]College of Pharmacy, Nanjing University of Chinese Medicine, Nanjing 210023, China
[5]School of Pharmacy, Shanghai University of Traditional Chinese Medicine, Shanghai 201203, China
[6]These authors contributed equally
*Correspondence: congzt@cdutcm.edu.cn

Received: August 13, 2024; Accepted: December 22, 2024; Published Online: January 2, 2025; https://doi.org/10.1016/j.xinn.2024.100774

© 2024 The Author(s). Published by Elsevier Inc. on behalf of Youth Innovation Co., Ltd. This is an open access article under the CC BY license (http://creativecommons.org/licenses/by/4.0/).

Citation: Bi Z., Li H., Liang Y., et al., (2025). Emerging paradigms for target discovery of traditional medicines: A genome-wide pan-GPCR perspective. The Innovation **6(3)**, 100774.

Traditional medicines serve not only as an integral part of medical treatments prescribed by healthcare providers but also as a fundamental reservoir for novel molecular scaffolds. However, gaps remain in our understanding of the mechanisms underlying their activity. A superfamily of membrane proteins, G protein-coupled receptors (GPCRs), have been demonstrated to be potential targets for several compounds isolated from traditional medicines. Given that GPCRs serve as targets for approximately one-third of all marketed drugs, they may be compelling targets for repurposing traditional medicines. Despite this potential, research investigating their activity or potential ligands across GPCRome, the library of human GPCRs, is scarce. Drawing on the functional and structural knowledge presently available, this review contemplates prospective trends in GPCR drug discovery, proposes innovative strategies for investigating traditional medicines, and highlights ligand screening approaches for identifying novel drug-like molecules. To discover bioactive molecules from traditional medicines that either directly bind to GPCRs or indirectly modify their function, a genome-wide pan-GPCR drug discovery platform was designed for the identification of bioactive components and targets, and the evaluation of their pharmacological profiles. This platform aims to aid the exploration of all-sided relations between traditional medicines and GPCRome using advanced high-throughput screening techniques. We present various approaches used by many, including ourselves, to illuminate the previously unexplored aspects of traditional medicines and GPCRs.

INTRODUCTION

Traditional medicines, sourced from herbs, medicinal animals, and fungi, have been used worldwide for centuries.[1] The 76th World Health Assembly in May 2023 formulated a new World Health Organization Global Traditional Medicine Strategy for the period spanning 2025 to 2034, aiming to promote evidence-based practice and the implementation of traditional medicine.[2] A comprehensive understanding of the therapeutic mechanisms underlying traditional remedies is essential for the discovery of new drug targets and devising cutting-edge therapeutic strategies. The inherent complexity of traditional medicines necessitates innovative and sophisticated methodologies to identify their active components and targets. High-throughput screening (HTS), a key process in modern drug discovery, enables the rapid evaluation of thousands to millions of compounds to identify potential lead candidates.[3,4] HTS techniques are instrumental in the efficient assessment of pharmacological activity and have garnered considerable attention in the field of traditional medicine.

For several decades, computational and experimental HTS of G protein-coupled receptors (GPCRs) has been vital for new drug discovery.[5,6] At present, approximately one-third of the US Food and Drug Administration (FDA)-approved drugs target GPCRs.[7] As the largest family of membrane proteins, GPCRs play a pivotal role in almost all essential physiological processes by translating extracellular stimuli into intracellular actions.[8] Moreover, GPCRs are emerging as crucial drug targets for a wide spectrum of pathological processes, which encompass, but are not limited to, metabolic syndromes, gastrointestinal pathologies, neuropsychiatric conditions, cardiovascular diseases, and neurodegenerative disorders.[9–11] Several studies have shown that traditional medicine components could interact with GPCRs. For instance, ephedrine and pseudoephedrine, isolated from *ephedra* plant species, have been shown to target the adrenergic receptors, thereby augmenting the release of norepinephrine from sympathetic neurons.[12] Dong et al.[13] also identified that ephedrine, extracted from ChuanbeiPipa dropping, a traditional Chinese medicine (TCM) remedy used for relieving cough and reducing sputum, functions as an agonist of the β_2-adrenergic receptor. Zhu et al.[14] showed that oridonin, an active component isolated from *Rabdosia rubescens* that is widely used in TCMs, offers a promising lead compound for metabolic disorder treatment by activating the bombesin receptor subtype 3. These findings suggest that traditional medicines could be an invaluable repository of compounds with therapeutic potential because of their GPCR modulation properties.

Frequently, only the key active components of traditional medicines have been identified, many of which have an extensive history of use that aligns with their contemporary medical applications and traditional roles. Notable examples include opium, quinine, artemisinin, and paclitaxel, all renowned as once being best-selling drugs.[15,16] However, the simultaneous presence of diverse components, which collectively augment the medicinal impact, supports the idea that traditional medicines target numerous biological pathways.[17] Thus, overcoming the barriers associated with the complexity of traditional medicines has the potential of unlocking a wealth of therapeutic opportunities. Given that GPCRs are closely related to the progression of several diseases often involving multiple receptor subtypes across various cellular and tissue contexts, the active ingredients of traditional medicines may exert multi-target and multi-pathway therapeutic effects, at least partially, by modulating GPCRs.[18–21]

Despite promising insights, there remains a significant gap in systematic information regarding the therapeutic potential of traditional medicines targeting GPCRs. To address this gap, we summarize recent advancements in the discovery of GPCR ligands from traditional medicines, highlighting the diversity of natural products, extensive array of GPCR targets involved, key screening techniques used, and the impact of traditional medicines on signaling pathways. These aspects reveal emerging trends and obstacles, focusing on the quest for mining new compounds that can engage in both orthosteric and allosteric interactions with GPCRs. Additionally, based on the current bottleneck, we propose a thorough strategy for conducting GPCR ligand screening of traditional medicines, i.e., the genome-wide pan-GPCR drug discovery platform, and analyze its potential applications. This platform seeks to investigate all GPCRs simultaneously by using a uniform approach to establish GPCR-expressing cell lines and systematically examining the connections between traditional medicines and the GPCRome.

IDENTIFICATION OF GPCR LIGANDS FROM TRADITIONAL MEDICINES

Traditional medicines have consistently served as a major source of new therapeutics worldwide. A variety of herbs, including *Artemisia annua*,[22] *Salix alba* (white willow),[23,24] *Catharanthus roseus*,[25] *Crataegus anamesa*,[26] and *Illicium verum*[27] have been cited as contributors to the development of new therapeutic agents. Over the past two decades, approximately 30% of the new drugs have been reported to originate from natural sources,[28] underscoring the importance of traditional medicines in contemporary medical advancement. Given that GPCRs are the most exploited drug targets, we provide an overview of GPCR ligands derived from traditional medicines such as medicinal herbs, animals, and fungi (Tables S1 and S2). Hitherto, at least 16 GPCR-targeting drugs approved by the FDA originate from natural products or their derivatives, with only 5 of them

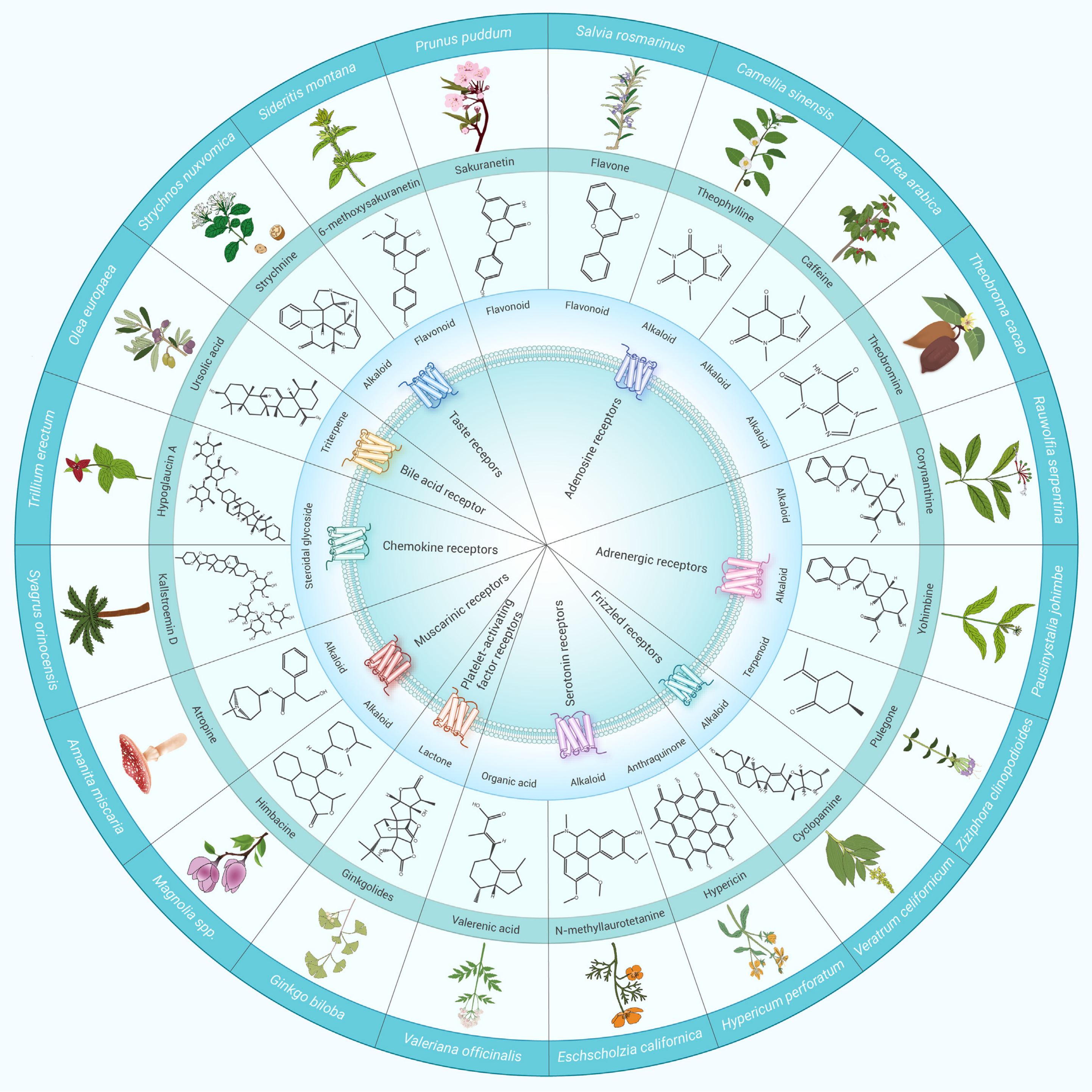

Figure 1. Diversity of GPCR ligands derived from traditional medicines The representative ligands listed in the middle circle exhibit diverse chemical structures, encompassing alkaloids, flavonoids, organic acids, and terpenoids. These compounds sourced from a variety of herbs that are shown in the outermost circle, such as *Ginkgo biloba*, *Sideritis montana*, and *Pausinystalia johimbe*.

having entered the market in the last 20 years (Table S3), indicating that the development of traditional medicines may have reached a bottleneck.

Chemical diversity of the components derived from traditional medicines

A recent systematic survey demonstrated that more than 600 unmodified natural products were isolated from traditional medicines until 2018; these have been demonstrated to modulate GPCRs, of which 66% were derived from medicinal plants.[29] This number would be significantly higher if modified molecules were included. GPCR ligands include various small molecules and peptides, with small molecules predominantly sourced from herbs and peptides from medicinal animals or fungi. The chemical diversity of the non-peptides is quite conspicuous, and includes alkaloids, flavonoids, furanochromones, glycosides, steroidal glycosides, and terpenoids (Figure 1). Among these, alkaloids accounted for the greatest proportion, with at least 11 FDA-approved GPCR-targeting drugs being alkaloids (Table S3). Morphine, derived from *Papaver somniferum*, is a prime example, effectively targeting and activating opioid receptors (ORs) to provide potent analgesic effects.[30] Flavonoids and terpenoids are also usually attainable GPCR ligands. For instance, gambogic acid, a natural prenylated xanthone, acts as an antagonist of GPR108, inhibiting tumor development via the nuclear factor-κB signaling pathway.[31] Celastrol, a representative terpenoid exhibiting anti-inflammatory and anti-fibrotic properties, functions as a selective agonist of the cannabinoid receptor 2 (CB2).[32]

Peptides, including plant-derived cyclotides, animal-derived oligopeptides, and polypeptides, have also been identified as GPCR ligands. For instance, the cyclotide Kalata B7, initially identified in the oxytocic plant *Oldenlandia affinis*, has been demonstrated to elicit robust contractility in uterine smooth muscle cells through the oxytocin and vasopressin 1A receptors.[33] The cyclic undecapeptide cyclosporin A, derived from the fungus *Tolypocladium inflatum*, exhibits potent immunosuppressive activity by effectively inhibiting the binding of formyl peptides to the formyl peptide receptor.[34] The peptide drug exendin-4 (brand name Byetta), derived from the *Heloderma suspectum* (Gila monster), was introduced in 2005 for the treatment of type 2 diabetes by activating the glucagon-like peptide-1 receptor (GLP-1R).[35] In addition to peptides, other ingredients derived from medicinal animals are also widely used, such as tauroursodeoxycholic acid—used in TCM for more than 3,000 years—shows significant anti-inflammatory effects, and enhances vasodilation in the heart by targeting G protein-coupled bile acid receptor 1 (also known as TGR5).[36,37] These examples further illustrate the profound impact of traditional medicines on contemporary pharmacology. Their diverse chemical structures, abundant functional properties, and complex stereoisomers endow traditional medicines with unique biological activities and immense potential as drug leads. The understanding of the diverse array of GPCR members targeted by traditional medicines offers valuable insights into their molecular mechanisms and guides current efforts in drug discovery to leverage their therapeutic potential.

Ligand binding-based GPCR ligand screening from traditional medicines

Traditional medicines exert their effects by interacting directly or indirectly with various GPCR subtypes. The majority of previously developed drug screening assays relied on the process of GPCR activation, involving ligand binding, signal transducer coupling, guanosine triphosphate (GTP)/guanosine diphosphate (GDP) exchange, second messenger releasing, and transcription regulation (Figure 2A).[38] Ligand screening remains a central focus of GPCR drug discovery. Among conventional techniques, the competitive ligand-binding assay (CLBA) stands out because of its high specificity and sensitivity and is widely used in characterizing the interaction between GPCRs and their ligands. As depicted in Figure 2B, CLBA is a commonly used method to quantify the interaction between GPCRs and a radiolabeled ligand by titration with the molecule of interest.[39,40] An alternative technique for determining ligand binding is the scintillation proximity assay, which relies on radioactive scintillation for signal detection.[41] However, its application is limited by its reliance on radioisotopes. To solve this problem, nonradioactive assays have emerged as alternatives, such as label-free fluorescent ligands, surface plasmon resonance (SPR), and fluorescence polarization techniques.[42–44]

Signal transducer coupling-based GPCR ligand screening from traditional medicines

The detection of the activation of a specific signaling pathway relies on the interactions between GPCRs and intracellular transducers.[5] Several assays based on fluorescence resonance energy transfer (FRET), NanoLuc Binary Technology (NanoBiT), and bioluminescence resonance energy transfer (BRET) have been developed and widely used for the detection of these interactions. FRET involves energy transfer between two fluorescent molecules, a donor and an acceptor, each attached to a protein of interest. When the donor is excited by a specific wavelength, it can transfer energy to the acceptor if they are in close proximity, leading to the emission of fluorescence from the acceptor; this indicates their interaction.[45] BRET operates similarly; however, it uses a bioluminescent donor, usually a luciferase enzyme, which emits light upon the addition of a substrate.[46] NanoBiT, a split-luciferase complementation assay, usually uses NanoLuc, which consists of two complementary subunits, Large BiT and Small BiT. Protein interaction brings these two subunits together, reconstituting an active luciferase enzyme and producing a luminescent signal.[47] In addition, the activation of GPCRs results in a conformational change within the heterotrimeric G proteins, thus providing another way to reflect receptor activation through the measurement of the proximity between Gα and G$\beta\gamma$ subunits. Upon receptor activation, the Gα subunits exchange their GDP for GTP, facilitating the separation of Gα and G$\beta\gamma$ subunits; subsequently, generation of second messengers takes place, with a cascade of cellular events.[48] TRUPATH is a BRET-based platform that enables the monitoring of Renilla luciferase-fused Gα with GFP2-fused Gγ subunits, which quantifies the activity of receptors through examination of the dissociation of heterotrimeric G proteins (Figure 2C).[49] The exchange of GTP/GDP represents a pivotal step in the activation of G proteins. Based on this process, more widely applicable assays have been developed to assess receptor activation. For example, in the quest for agonists targeting the serotonin 1A receptor, Nishi et al.[50] performed a GTPγS binding assay with seven primary alkaloids found in *Uncaria rhynchophylla*.

The cytosolic β-arrestins serve to impede G protein engagement by steering the receptor through desensitization, internalization, and trafficking processes, making them a more accessible target for detecting GPCR translocation or activation.[51] The process begins with the phosphorylation of the C terminus by GPCR kinases, which signals to β-arrestins to induce receptor desensitization (Figure 2A).[52] β-arrestin recruitment assays are particularly valuable for revealing the nuances of functional selectivity in GPCR signaling, i.e., biased agonism, offering insights that could mitigate unwelcome side effects.[53] Apart from the above-mentioned BRET, FRET, and NanoBiT assays, various non-luminescence-based β-arrestin recruitment assays (such as the Tango and PathHunter assays) expand the toolkit available for this type of investigation (Figure 2C).[54,55] Among these methods, the Tango assay stands out for its use of a protease-activated reporter gene system, which offers the advantages of enhanced signal specificity and improved efficiency.[56] For instance, by using the piggyBac-Tango assay, it was demonstrated that atractylon could efficiently activate the dopamine 2 receptor,[57] showcasing the usefulness of these innovative approaches in GPCR ligand screening.

Downstream signaling-based GPCR ligand screening from traditional medicines

While G protein-dependent functional assays play a crucial role in GPCR research, they are limited by their inability to precisely identify the G protein subtypes involved. As shown in Figure 2A, the Gα units segregate into four subfamilies including G_s, $G_{i/o}$, $G_{q/11}$, and $G_{12/13}$, each coordinating distinct signaling pathways.[58] The intracellular cyclic adenosine monophosphate (cAMP) level can be determined as a readout for G_s- and $G_{i/o}$-coupled receptors and calcium ion (Ca^{2+}) or inositol trisphosphate level for $G_{q/11}$-coupled receptors. Recent technological advancements have opened new avenues for exploring the specific interactions between G proteins and GPCRs in live cells by measuring related downstream events and the downstream activation of gene promoters.[38] Traditional medicines interact with G protein downstream signaling pathways in multiple ways. For instance, FRET-based cAMP assays have demonstrated that icaritin, an active chemical in *Epimedium koreanum*, selectively antagonized the muscarinic acetylcholine M2 receptor, and the measurements of intracellular Ca^{2+} levels confirmed this specificity.[59] Compared with conventional HTS methods based on downstream signaling assays, the second messenger-responsive elements, such as the cAMP response element and serum response element, are all critical in mediating gene transcription after GPCR activation and provide several highly sensitive and efficient HTS assays. For example, luciferase reporters have been developed as a common HTS platform for GPCR drug discovery.[60] This assay has facilitated the identification of gambogic acid, a natural prenylated xanthone that selectively targets GPR108 and promotes its degradation.[31]

FUNCTIONAL MODULATION OF GPCRs BY TRADITIONAL MEDICINES

Direct modulation of GPCR signaling by traditional medicines

Recent research has revealed that numerous compounds from traditional medicines exert therapeutic effects by targeting GPCRs. Table S1 provides a non-exhaustive selection of more than 80 representative articles reporting *in vitro* or *in vivo* evidence of direct modulation of GPCR signaling by traditional medicines. For instance, curcumin, the principal polyphenolic extract derived from *Curcuma longa*, has long been used in traditional medicines. It plays a multifaceted role in modulating adenosine receptors and enhancing the efficacy of purinergic $P2Y_{12}$ receptor ($P2Y_{12}R$) inhibitors within platelets.[61] Competitive binding assays conducted in HEK293 cells overexpressing A_{2A} adenosine receptor ($A_{2A}AR$) and A_{2B} adenosine receptor demonstrated curcumin's binding affinity; however, no consistent agonistic or antagonistic activity was observed with $A_{2A}AR$ and $P2Y_{12}R$.[62,63] Therefore, the physiological effects of curcumin's interaction with these receptors need further validation. In addition to adenosine receptors, curcumin and its structurally related compounds also have been reported to activate GPCR 55 (GPR55) and GPCR 97 (GPR97). The activation of

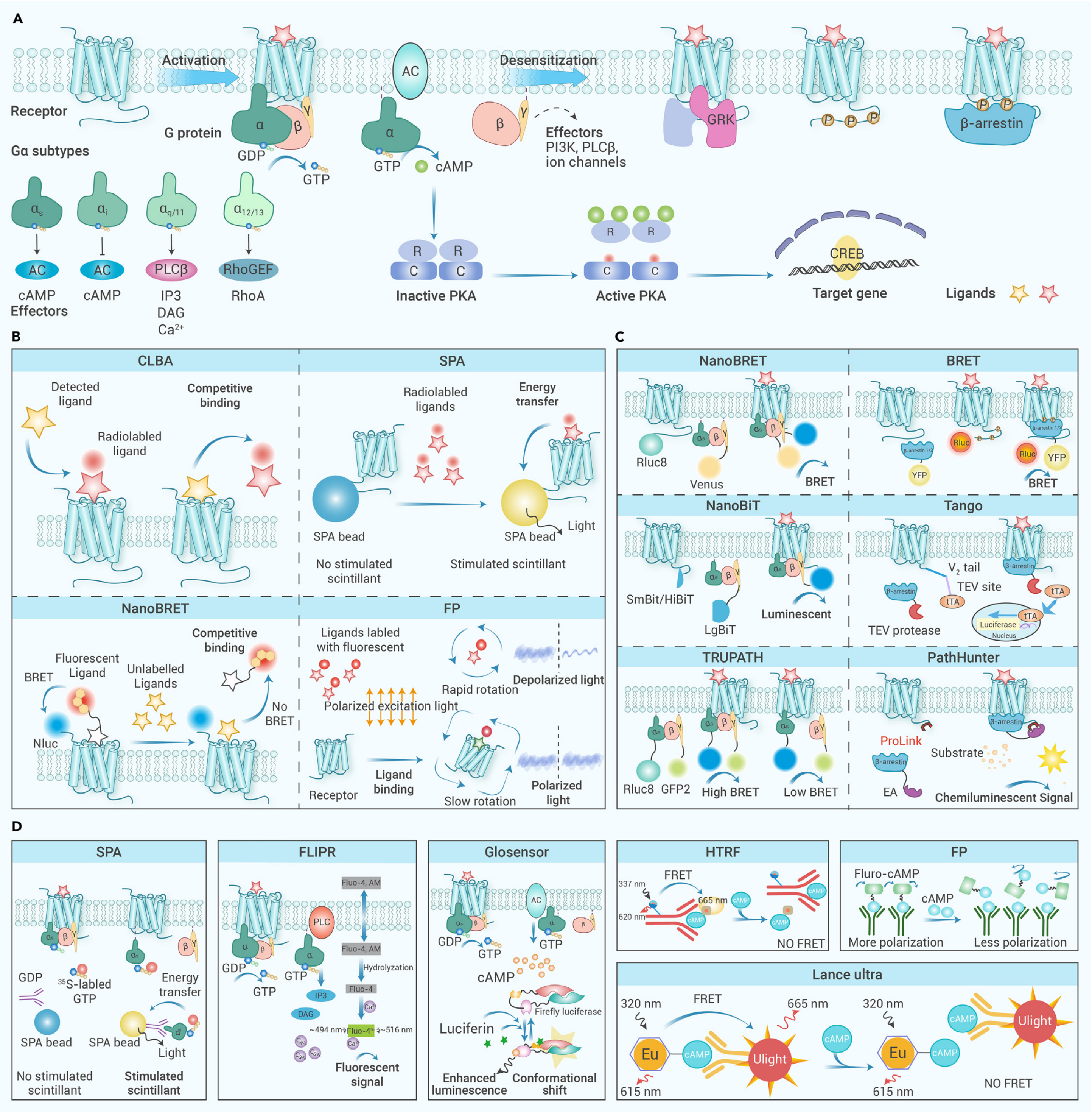

Figure 2. HTS methods based on GPCR pharmacology profiling (A) GPCR activation is characterized by a series of sequential events, encompassing ligand binding, G protein coupling, G protein activation, receptor desensitization, and β-arrestin recruitment. (B) Techniques for detecting ligand binding. (C) Techniques for detecting G protein coupling and β-arrestin recruitment. (D) Techniques for detecting G protein activation and related downstream signaling. FLIPR, fluorescent imaging plate reader; FP, fluorescence polarization; HTRF, homogeneous time-resolved fluorescence; SPA, scintillation proximity assay.

GPR55 by curcumin led to its coupling with $G_{12/13}$ and GLP-1 secretion, suggesting its regulatory role in glucose homeostasis through GPR55.[64] The discovery that curcumin activated GPR97 implies its potential involvement in inflammatory regulation, given the known effects on inflammation and the predominant expression of GPR97 in leukocytes.[65] Furthermore, there were suggestions that GPR40 is another potential target for curcumin, since GLP-1 secretion induced by curcumin was significantly reduced after the administration of a GPR40 antagonist to rats.[66] Given that curcumin acts as a potent regulator across various diseases, including neurological disorders, inflammatory diseases, diabetes, and cancer,[67] further research is anticipated to elucidate the precise role of these potential targets.

Osthole, derived from the fruits of *Cnidium monnieri* and widely used in traditional medicines, has been demonstrated to reduce the expression levels of the Mas-related G-protein coupled receptor member X2 (MRGPRX2) in mast cells.[68] Osthole is known for its diverse pharmacological activities, including anti-inflammatory,[69] antidiabetic,[70] antiasthmic,[71] and antitumor properties.[72] Since MRGPRX2 plays a pivotal role in mediating pseudo-allergic reactions and chronic inflammation associated with asthma, urticaria, and rosacea, this

study provides a strong rationale for investigating osthole as a novel treatment option for pseudo-allergic conditions.[73] Molecular docking analyses suggested that osthole could not compete with MRGPRX2 agonists, but rather regulated MRGPRX2 activation through allosteric modification, mainly by attenuating Ca^{2+} mobilization *in vitro* and inhibiting inflammation in mouse models of pseudo-allergy.[73] While molecular docking approaches provide advanced structure-activity relationship analyses, more accurate structural information of ligand-receptor interactions is expected to be provided by use of cryo-electron microscopy (cryo-EM) or X-ray techniques.

Gintonin, a glycolipoprotein derived from *Panax ginseng* that is widely used as a tonic in traditional medicines for centuries, acts as an exogenous ligand for lysophosphatidic acid (LPA) receptors.[74] In addition, gintonin could also regulate insulin secretion and cell migration by activating GPR40 and GPR55. Gintonin enhanced insulin secretion from INS-1 cells in a dose-dependent manner, which was partially inhibited by a GPR40 antagonist, but not by antagonists of the LPA1/3 receptor. Furthermore, gintonin could induce Ca^{2+} signaling-dependent cell migration in PC-3 cells in a dose-dependent manner, which could be attenuated by pretreatment with antagonists of both GPR55 and LPA1/3 receptors or through down-regulation of GPR55 using small interfering RNA.[75] The binding pattern of gintonin to these receptors and the potential coordination between them necessitate further investigation. Neferine, a small molecule extracted from green embryos, has been highlighted as a promising orexin receptor antagonist, holding promise as a lead compound in the development of new therapeutics for insomnia and orexin-induced disorders.[76] ISP-1 is a metabolite of the fungus *Isaria sinclairii* that is widely used in TCM; it possesses a sphingosine-like structure.[77] It was ultimately modified to FTY720 and a series of FTY720 derivatives which bind to the sphingosine 1-phosphate receptor, thereby exerting immunosuppressive effects.[78]

Traditional medicines have also been observed to regulate β-arrestin signaling pathways. Desensitization and internalization are critical feedback mechanisms that safeguard cells against excessive stimulation.[79,80] Certain ingredients derived from traditional medicines may directly engage with pre-activated β-arrestins or facilitate receptor phosphorylation through kinase interactions, thus directing β-arrestins to be involved in alternative signaling pathways. For instance, *Dendrobium officinale*, known for its medicinal and edible values, produces polysaccharides possibly blocks β-arrestin 1-related signaling pathways.[81] The administration of the *D. officinale* polysaccharides (DOPS) significantly ameliorated clinical symptoms of ulcerative colitis and reduced mortality in a mouse model, indicating its potential therapeutic role in treating ulcerative colitis and other inflammatory diseases. These effects were attributed to the inhibition of NLRP3 inflammasome activation and β-arrestin 1 signaling pathway both *in vitro* and *in vivo*; however, the direct interaction between DOPS and a certain GPCR has not been demonstrated.[82] The therapeutic value of *Typha angustifolia*, a Chinese herb, has been acknowledged for millennia and demonstrated to possess potential to mitigate insulin resistance. The derivatives of total flavone extracts from *T. angustifolia* have been shown to enhance insulin-stimulated glucose uptake in C2C12 myotubes by preventing palmitate-induced insulin resistance in dose- and time-dependent manners, increasing the expression of β-arrestin 2, and promoting phosphorylation of key proteins, which indicates a pharmacological effect via β-arrestin 2-mediated signaling pathways.[83] These findings provide insights into the mechanisms by which traditional medicines may exert pharmacological properties, thereby offering new perspectives on the therapeutic potential of traditional medicines containing similar molecules.

The G protein and β-arrestin pathways are distinct both temporally and spatially and may mediate specific physiological or pathophysiological outcomes.[84] For instance, the angiotensin II type 1 receptor facilitates intense vasoconstriction and increases blood pressure by activating G protein pathways while inducing potential benefits, such as stimulating contractility and exerting cytoprotection effects through β-arrestin pathways.[85] Thus, the biased ligands selectively activating either G protein or β-arrestin signaling could facilitate favorable outcomes while inhibiting detrimental or undesired effects. The discovery of GPCR ligands in traditional medicines has the potential to accelerate the development of drugs with biased agonistic properties. A recent study identified a natural alkaloid, columbamine, that can alleviate intestinal inflammation and promote efferocytosis by directly binding to the formyl peptide receptor 2 (FPR2) and selectively activating the FPR2-mediated cAMP signaling pathway.[86]

Indirect modulation of GPCR signaling by chemical components of traditional medicines

Traditional medicines have complex components that not only act on GPCR directly, but may exert therapeutic effects by regulating their endogenous ligands, pertinent enzymes, and second messengers. Table S2 provides a non-exhaustive selection of representative articles reporting *in vitro* or *in vivo* evidence of indirect modulation of GPCR signaling by traditional medicines. Traditional medicines have been found to exert therapeutic effects on GPCR by affecting activity of endogenous ligand, particularly that of peptide hormones and proteins. For instance, *Gentiana scabra* (GS) root extract has gained attention in traditional Korean medicine because of its potential therapeutic effects for diabetes. Pharmacological studies have suggested that GS may lower blood glucose levels by promoting the secretion of GLP-1, which subsequently enhances insulin secretion.[87] Ethanol extracts from fenugreek seeds have been found to reduce glucose and glycated hemoglobin levels by enhancing GLP-1 activity. Unlike classic allosteric modulators that bind to the transmembrane domain of GLP-1R, the isolated active compound N55 directly binds to the endogenous peptide and facilitates GLP-1-mediated signaling.[88] Natural products may also ameliorate diseases by influencing the secretion of second messengers or related enzymes, particularly by kinases and phosphatases. A peptide from the venom of the Brazilian pit viper (*Bothrops jararaca*) is a snake venom toxin that functions as an angiotensin-converting enzyme (ACE) inhibitor. This peptide specifically targets ACE, thereby inhibiting the production of angiotensin II, a potent vasoconstrictor that regulates blood pressure and fluid balance by acting on angiotensin receptors.[89]

BINDING POSES OF HERB-DERIVED COMPOUNDS AT GPCRS

To the best of our knowledge, the regulation of GPCR activity by several herb-derived compounds has been well demonstrated using computational docking, cryo-EM, and crystallography-confirmed binding poses. Precise identification of the binding sites of these molecules is crucial for structure-activity relationship (SAR) analysis. With the structural data available for numerous such molecules, comparison of their binding sites with those of native ligands is now feasible. Some of the molecules summarized in Table S1 were analyzed to provide further insights into their binding modes (Figure 3). It is worth noting that GPCRs with a higher number of herb-derived ligands, such as cannabinoid, opioid, adenosine, muscarinic, and adrenergic receptors, have undergone extensive structural and functional scrutiny.

Cannabinoid receptors

Cannabis sativa was documented a millennium ago in the renowned TCM book 'Shennongbencaojing' as possessing therapeutic potential.[90] Phytocannabinoids, compounds extracted from *Cannabis*, have garnered significant attention due to extensive research on Δ9-tetrahydrocannabinol (THC) and cannabidiol.[91,92] In the 1980s, two GPCRs, namely CB1 and CB2, were discovered and demonstrated to specifically respond to cannabinoids.[93,94] Several CB1 structures bound to analogs of cannabinoids have been elucidated in fully or partially active states, including receptors bound to the endocannabinoid anandamide analog AMG315, and the THC-like cannabinoids AM841, AM12033, and AM11542.[95,96] Overlaying these structures reveals that the agonists all adopt a similar L-shaped conformation, defining the orthosteric binding pocket (OBP). In particular, the acyl chain of AMG315 penetrates deeply into the OBP, whereas the polar head group residues extend into a positive cavity formed by the TM1–TM7 interface and interact with the extracellular loop 2 (ECL2) (Figure 3A). AM841 and AM11542 exhibit a similar conformation to the acyl chain of AMG315 within the core of the pocket, but occupy only a portion of its alkyl chain on the extracellular side, thus reducing contact with the extracellular ends of TM1 and TM7 (Figure 3A).

Activation of CB1 triggers a diverse array of signaling cascades by coupling to G proteins and β-arrestins. Each of these signaling pathways elicits distinct pharmacological responses, offering potential avenues for the development of novel therapeutics tailored to specific diseases.[97] The recent studies successfully resolved the structure of both FUB and AMG841-bound CB1, respectively, in complex with β-arrestin 1 (ArrB1), shedding light on the key structural determinants underlying CB1-mediated selective signal transduction.[98,99] Comparison with the structures of AM841-bound CB1-G_i complexes reveals nearly identical binding poses of the alkyl chain of AM841. However, the tricyclic ring adopts

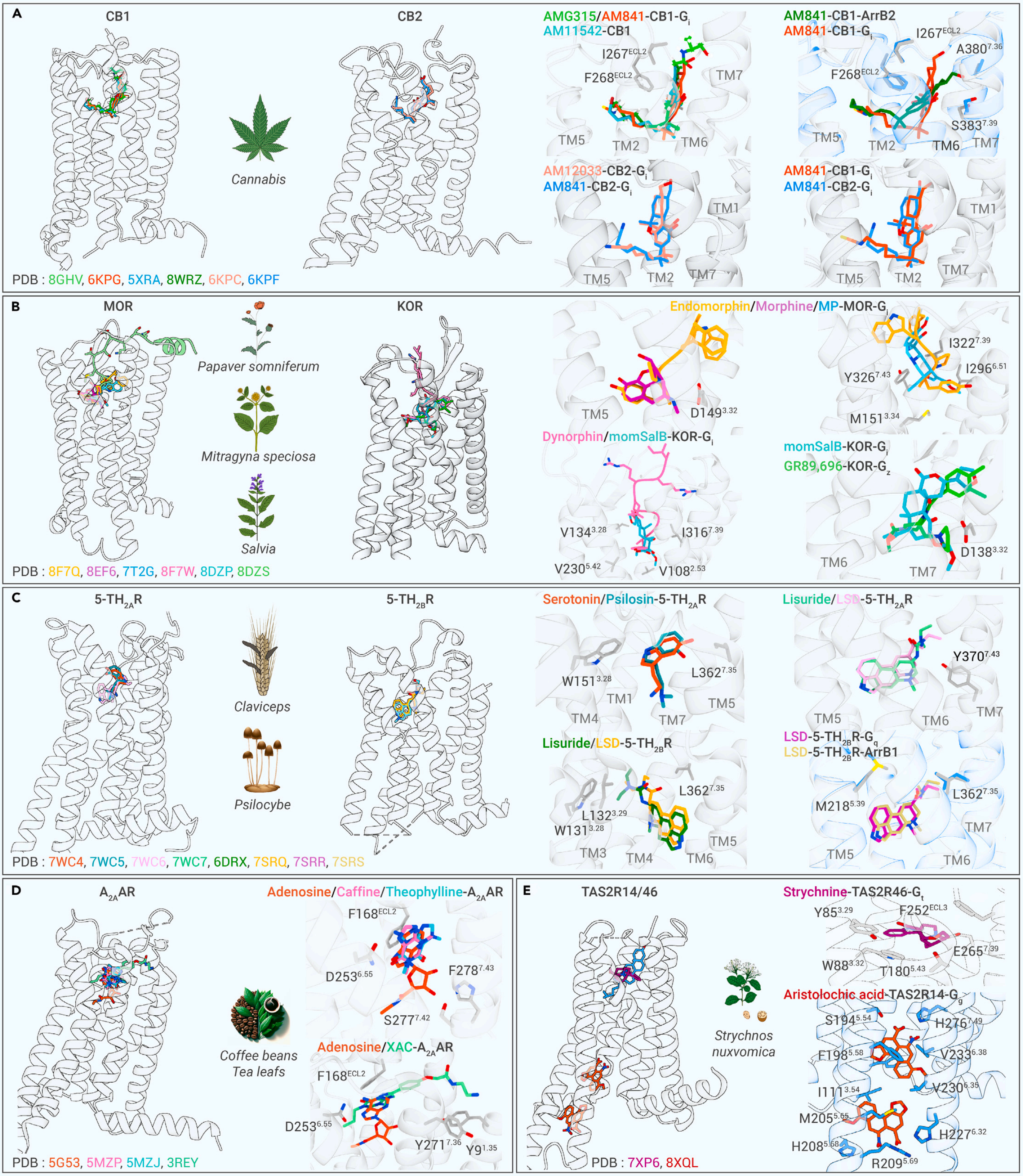

Figure 3. Binding poses of herb-derived compounds and their interactions with GPCRs (Left) Side views of receptors bound to endogenous and herb-derived molecules or their derivatives. (Right) Comparison of binding poses of endogenous ligands and herb-derived molecules or their derivatives. (A) The binding poses of anandamide analog (AMG351) and THC-like cannabinoids (AM841, AM11542, and AM12033) at CB1 and CB2. (B) The binding poses of endogenous peptides (endomorphin and dynophin) and herb-derived small molecules (morphine, mitragynine pseudoindoxyl (MP), and momSalB) at opioid receptors. (C) The binding poses of endogenous serotonin and herb-derived small molecules (psilosin, lisuride, and LSD) at 5-THRs. (D) Comparison of binding poses between adenosine and caffeine or theophylline at $A_{2A}AR$. (E) The binding modes of strychnine at TAS2R46, as well as cholesterol and aristolochic acid, at TAS2R14. The PDB numbers of the structures are shown in the bottom of each panel, with the number's color matching that of the molecule.

different positions within the core of the binding pocket (Figure 3A). In the AM841-bound CB1-ArrB1 structure, the tricyclic ring interacts with $F268^{ECL2}$ and $I267^{ECL2}$, whereas the phenolic hydroxyl group bends toward TM7, interacting with residues A380 and S383, potentially triggering the recruitment of β-arrestin (Figure 3A).

Similar to that of CB1, the N-terminus of CB2 also forms a short helix over the OBP. Although AM841 and AM12033 occupy similar positions within the pocket in CB2, the acyl moiety of AM841 is oriented toward ECL2 rather than TM4, as observed in CB1 (Figure 3A). Functional studies suggest that CB2-selective agonists may provide better therapeutic properties without inducing psychotropic side effects.[100] To overcome the side effects of *Cannabis* while harnessing its therapeutic potential, understanding the modulation and signaling mechanisms of cannabinoid receptors provided by these structures is essential. These results provide a robust foundation for the development of synthetic cannabinoids capable of optimizing their receptor activity.

Opioid receptors

Natural opioids, also known as opiates, derived from *P. somniferum*, possess a rich history as medicinal agents and are widely recognized as highly efficacious analgesics.[101] Their activity is mediated by human ORs, namely mu (μ), kappa (κ), and delta (δ) ORs (MOR, KOR, and DOR, respectively). Morphine, the representative opioid alkaloid, has long been used in medical practice for pain management, primarily acts on MOR.[102] Another analgesic alkaloid, mitragynine, derived from *Mitragyna speciosa*, is gaining attention for its purportedly reduced risk of addiction compared with morphine.[103,104] The 7-hydroxymitragynine is an oxidative metabolite that is 22 times more potent than mitragynine, and can be further converted to mitragynine pseudoindoxyl (MP) that exhibits greater activity as an MOR agonist.[105] The structures of the morphine or MP-bound MOR-G_i complexes observed using cryo-EM show that the binding poses of the two agonists are significantly different from the endogenous peptides. Morphine adopts an elliptically shaped binding pose, whereas MP is inserted deeply into the OBP with a similar binding pose as the tetrapeptide endomorphin (YPWF) and the N-terminus (YGGF) of β-endorphin (Figure 3B). The morphinan group is located at the bottom of the OBP and overlaps with the two N-terminal residues of endomorphin; the hydroxyl of the phenol moiety point toward TM5, forming salt bridges with the carboxylate group of $D149^{3.32}$ (Figure 3B). The superimposed structures demonstrate that MP aligns with the N-terminus of the endomorphin and penetrates the central pocket, forming more extensive contacts with the receptor core, involving residues $M151^{3.34}$, $I296^{6.51}$, $I322^{7.39}$, and $Y326^{7.43}$, which may ensure better activity and selectivity for MOR.

Salvinorin A is a terpenoid extracted from *Salvia divinorum* and identified as a group of natural hallucinogens by activating KOR.[106] To better understand how these molecules bind to KOR, the researchers synthesized a series of salvinorin A derivatives, including methoxymethyl-salvinorin B (momSalB), which have certain hallucinogenic effects.[107] Structural comparison indicates that momSalB occupied a similar bottom pocket as the N-terminal motif (YGGF) of dynorphin, and the residue $V108^{2.53}$ has been identified as a key factor in determining ligand specificity, as MOR or DOR uses alanine at the corresponding position.[108] The molecular mechanism of the differential binding and activation of KOR by alkaloid and terpenoid agonists was also revealed by structural studies. Both momSalB and GR89,696 exhibit high selectivity and potency at KOR. Although these two agonists share a common binding pocket within the OBP, their core rings occupy distinct perpendicular positions, resulting in different interactions with residues in the corresponding subpockets (Figure 3B). As the terpenoid lacks a basic nitrogen atom, the salvinorin ligand momSalB could not form electrostatic interactions with $D138^{3.32}$, indicating a different activation mechanism compared with alkaloid agonists.

Serotonin receptors

The synthetic derivative of ergoline alkaloids from ergot fungi, known as lysergic acid diethylamide (LSD), demonstrates high affinities toward almost all 14 serotonin receptors.[109,110] The binding mode of lisuride closely resembles that of LSD; however, it lacks psychedelic effects, primarily due to its partial and biased agonism of the $5\text{-}HT_{2A}R$. Additionally, as an antagonist of $5\text{-}HT_{2B}R$, lisuride does not pose a risk of cardiac valvulopathy, unlike ergolines.[111] Psilocybin is a naturally occurring psychedelic produced by the *Psilocybe* genus, which has been deliberately exploited by humans for thousands of years. Psilocybin itself is biologically inactive but is converted to psilocin *in vivo*, which has mind-altering effects similar to those of LSD.[112] Comparison of the ligand-bound $5\text{-}HT_{2A}R$ structures reveals that LSD and lisuride's ergoline moieties occupy the bottom of the OBP, while serotonin or psilocin's indole cores are located higher and closer to the extracellular space where it is occupied by the diethyl moiety of ergoline alkaloids (Figure 3C). The $L362^{7.35}F$ mutation had no effect on the extent of the G_q activation, but it prevented β-arrestin recruitment induced by psilocin and lisuride,[113] indicating that ligand bias is influenced by recognition in the extended binding pocket (EBP). Alignment of LSD- and lisuride-bound $5\text{-}HT_{2A}R$ exhibits a similar binding mode, yet distinct conformations near the extracellular region. Specifically, two ethyl groups of LSD interacted with residue $Y370^{7.43}$, whereas only one ethyl group of lisuride interacts with this residue (Figure 3C). Due to the closer interactions, the $Y370^{7.43}W$ mutation reduced both of the LSD-mediated G_q activation and β-arrestin 2 recruitment signaling.[113]

The comparison of LSD- or lisuride-bound $5\text{-}HT_{2A}R$ with $5\text{-}HT_{2B}R$ reveals a subtle distinction in the overall receptor conformation and LSD-binding pose (Figure 3C). Similar to that observed at $5\text{-}HT_{2A}R$, LSD binds to the typical OBP at $5\text{-}HT_{2B}R$ in both of the fully and intermediately active state,[109,114] with ECL2 forming a lid over the OBP, occluding LSD, which may prolong its residence time (Figure 3C). However, the binding mode of lisuride at $5\text{-}HT_{2B}R$ exhibits a subtly different positioning of the (S)-diethylurea motif, which conformationally mimics the diethylamide of LSD at $5\text{-}HT_{2A}R$ rather than pointing in the opposite direction at $5\text{-}HT_{2B}R$, making minimal contact with TM7 (Figure 3C). This likely explains why lisuride acts as an antagonist of $5\text{-}HT_{2B}R$, but as an agonist of $5\text{-}HT_{2A}R$.[111,115] When bound to $5\text{-}TH_{2B}R$, the (S)-diethylurea moiety of lisuride was uniquely wedged between residues in TM3 by hydrophobic stacking, whereas LSD is in contact with residue $L362^{7.35}$ in TM7, shaping a more contracted EBP (Figure 3C). These different binding poses likely explain why lisuride acts as an antagonist and LSD as an agonist of $5\text{-}HT_{2B}R$. Comparison of G_q- and ArrB1-coupled $5\text{-}HT_{2B}R$ shows that most residues in the binding pocket exhibit nearly identical side-chain conformations upon LSD binding.[109] Subtle conformational changes are only observed in residues $M218^{5.39}$ and $L362^{7.35}$ (Figure 3C), highlighting the essential role of interactions between TM5 and TM7, respectively, and LSD in facilitating β-arrestin recruitment.

Adenosine receptors

The three major methylxanthines are caffeine, theophylline, and theobromine, all of which are mainly found in coffee beans (Rubiaceae) and tea leaves (*Camellia sinensis*). Adenosine receptors are known for their antagonists, which produce the stimulating and energizing effects of coffee, tea, and chocolate.[116] The psychostimulants caffeine and theophylline not only act on $A_{2A}R$, but also have a weak affinity for the other three receptor subtypes; thus, they can be considered pan-antagonists of the human adenosine receptors. The overlayed structures within the OBP demonstrate a nearly exact superimposition of the core of theophylline and caffeine with the adenine moiety of adenosine, whereas xanthines amine congener (XAC) exhibits a more extensive binding pocket (Figure 3D). All antagonists form hydrogen bonds with $N253^{6.55}$, but show no interactions with $S277^{7.42}$ or $H278^{7.43}$, which is characteristic of binding sites of agonist, and block conformational changes observed in agonist-bound structures. The polar tail of XAC resides within a groove formed between $Y9^{1.35}$ and $Y271^{7.36}$ in the top of TM1 and 7, while caffeine binds to a position close to the xanthine portion of XAC and the triazolotriazine core of adenosine (Figure 3D). $A_{2A}R$ shows significant contraction of its pocket upon agonist binding,[117] suggesting that the expanded binding pocket observed with antagonists may be attributed to the maintenance of its inactive conformation. The variation in binding modes and the diversity of antagonist scaffolds illustrate the complexity of the molecular mechanisms of these herb-derived compounds.

Bitter receptors

In humans, bitter taste is mediated by 25 GPCRs that belong to the TAS2R subfamily.[118] Bitter compounds derived from herbs encompass a variety of chemical classes, such as phenolics and secoiridoids,[119] picrotoxinin,[120,121] andrographolide, and amarogentin.[122] The poisonous alkaloid strychnine that was found in the seeds of the *Strychnos nux-vomica* tree and used as traditional medicines in China and South Asia, was demonstrated to activate three bitter receptors including TAS2R7, TAS2R10, and TAS2R46.[123,124] Strychnine is also used as a pesticide because of its neurotoxicity mediated by high-affinity binding to the

glycine receptor.[125] Currently, particular attention is given to its SAR at TAS2R46 due to recently published high-resolution cryo-EM structures.[126] TAS2R46 exhibits responsiveness to a wide range of bitter substances, with strychnine being the most potent agonist identified thus far.[123,127] Before agonist recognition, the OBP resembles a wide-open funnel and occupied by ECL2, which results in the pre-activation of the receptor. Upon binding, the strychnine is shaped in a baseball cap binding pose within the OBP (Figure 3E). G protein precoupling has been speculated to facilitate a rapid response by TAS2R46 to protect organisms against intoxication.[126]

Recently, three studies have reported the structures of TAS2R14 in complex with G proteins, revealing an OBP occupied by cholesterol and intracellular allosteric sites for various bitter tastants.[128–130] TAS2R14 is unique among bitter taste receptors mainly because of its ability to recognize diverse bitter molecules and its extra-oral expression through effects on tissues of the respiratory, cardiovascular, and digestive systems, highlighting its potential therapeutic applications beyond taste perception.[131] The findings suggested that TAS2R14 may function as a sensor of metabolites of bile acids, which are known for extreme bitter taste and used in TCM for reducing fever, anti-inflammation, aiding digestion, and relieving respiratory issues.[132] Computational and biochemical studies confirm that several bile acids bind as orthosteric agonists to TAS2R14, indicating that medicinal bile acids may pharmacologically target bitter receptors.[129,133] Noteworthily, the structure of aristolochic acid (AA) bound-TAS2R14 reveals two intracellular pockets,[130] one formed by TMs 3 and 5–7 and the Gα5 helix's cytoplasmic end, and another shaped by TMs 5–6's cytoplasmic end and the Gα5 helix (Figure 3E). The multiple binding poses of AA on TAS2R14 may be associated with the rapid expression of toxicity, given that AA is a toxic carcinogen present in many traditional medicines, such as *Aristolochia manshuriensis*, *Aristolochia fangchi*, *Aristolochia debilis*, and *Aristolochia cucurbitifolia*.[134,135]

The aforementioned information suggests that there are a limited number of receptors that have been structurally decrypted in the presence of herb-derived molecules, and almost all binding sites were observed in the orthosteric pockets. The precise binding mode of numerous traditional medicine-derived molecules remains unclear, with possibilities the discovery of binding to entirely allosteric sites. The binding modes of these molecules are expected to be determined definitively through structural studies.

GPCRome-BASED HTS SHEDS LIGHT ON THOROUGH INVESTIGATION OF TRADITIONAL MEDICINES

The scarcity of the identification of potential GPCR ligands derived from traditional medicines is not surprising, given the limited availability of resources and screening tools. We believe that there are still undiscovered associations between traditional medicines and GPCRs and a comprehensive approach would unveil novel binding sites. However, conventional technological limitations constrain the current exploration of their interactions with GPCRs. For example, the usefulness of ligand binding-based screening methods is limited by the large number of GPCRs that require radiolabeled ligands with specific activity and high affinity for the given targets, which are often unavailable.[39]

HTS based on ligand-binding assays

Certain emerging detection techniques, such as SPR, cell membrane chromatography (CMC), and affinity mass spectrometry (A-MS), enable efficient binding-based screening due to their many-to-one throughput (many ligands to one target) (Figure 4A). The SPR assay is an optical technique used for the real-time measurement of receptor-ligand interactions, as the SPR signal can be detected when plane-polarized light hits a metal film.[136] Additionally, advancements in the expression and purification of recombinant GPCRs have facilitated the determination of interaction properties of several herb-derived molecules using affinity chromatography technique, which relies on the maintenance of the complete biological structure of a membrane receptor.[137] The CMC system, equipped with biorecognition and boundary electrical sensing functions, exhibits the capability to selectively detect components from complex systems.[138] Using this technique, researchers have identified five potential active compounds from extracts of the TCM *Schisandra chinensis* that acts on the transmembrane protein differentiation 20.[139] Recently, A-MS, another affinity analysis technique, has also been developed for the detection of ligand binding, which ensures the structural integrity of both the receptor and ligand. As a non-destructive and label-free technique, A-MS is particularly valuable when dealing with structurally fragile or complex molecules.[140] In A-MS assays, native GPCRs are typically incubated with a mixture of compounds, each possessing unique molar masses. Due to the high sensitivity, A-MS can detect and quantify even weak interactions between GPCRs and potential ligands, which eliminates the need for the separation of natural monomers during the screening stage, and is therefore particularly suitable for the identification of active components of herbal extracts. For instance, Zhang et al.[141] presented an A-MS assay and successfully identified active ligands of 5-HT_{2C}R by screening several herbal extracts. These features render CMC and A-MS versatile systems, well suited for the direct identification of ligand-receptor interactions from crude extracts of traditional medicines.

HTS based on downstream signaling assays

Usually, simultaneously meeting the physical, informatics, and infrastructure requirements of the routine screening of multiple GPCRs exceed the financial capabilities of most academic or industrial laboratories. Highly multiplexed bioactivity-screening technologies are necessary to overcome the limitations of conventional HTS techniques for screening of bioactive components from complex systems. The current trend in biomedical research involves the integration of multi-omics approaches, encompassing genomics, transcriptomics, and proteomics data layers, thereby optimizing the processes involved in drug discovery.[142–144] A primary objective of the GPCRome study (GPCRomics) is to identify and characterize the endogenous GPCRs that are involved in both physiological and pathological conditions.[145] To the best of our knowledge, only four open-source biosensor screening platforms are currently available for interrogating the entire druggable GPCRome, including the Dcy-FIR, ONE-GO biosensor, PRESTO-Tango, and PRESTO-Salsa (Figures 4B–4E).

The PRESTO-Tango assay was developed to facilitate the interrogation of the druggable human GPCRome through β-arrestin recruitment, making it an ideal one-to-many functional screening tool.[56] Upon ligand binding, the GPCR-TEV-tTA is activated and recruits the stably transfected β-arrestin-TEV protease. This process enables the tTA transcription factor to enter the nucleus and initiate the expression of the luciferase reporter gene (Figure 4B). As early as 2015, this assay was expanded to encompass more than 300 GPCRs by adding the C-terminus of the vasopressin receptor for efficient β-arrestin recruitment.[56] Lately, PRESTO-Tango technology was upgraded to PRESTO-Salsa by combining barcoding technology with gene sequencing, which enables simultaneous assessment of almost all conventional GPCRs (Figure 4C). PRESTO-Salsa has been successfully used to investigate the diverse landscape of interactions between the human microbiota metabolome and GPCRome,[146] suggesting the feasibility and potential of these platforms for drug discovery from traditional medicines.

The ONE-GO biosensor serves as a scalable platform for measuring G_α-GTP across different G protein types, allowing accurate measurement of G protein activation by any GPCR (Figure 4D).[147] The universal applicability of ONE-GO biosensor is underscored by its effectiveness across a diverse array of G proteins and receptors and its successful implementation in various cell types, including primary cell lines. This versatility makes it a powerful tool in GPCR research and drug discovery. The DCy-FIR platform, using a yeast strain library with 300 engineered potential GPCR-Gα coupling combinations (Figure 4E), has successfully replicated known GPCR agonism with a remarkable accuracy rate of 100%. Moreover, it revealed several unforeseen interactions, showcasing its exceptional capability to identify GPCR ligands.[148] Given the potential multi-targeting nature of ligands derived from traditional medicines, the DCy-FIR platform holds immense promise for simultaneously profiling numerous GPCRs against individual components. While these platforms were originally designed for detailed pharmacological purposes, this review showcases their potential for high-throughput investigations of GPCRs and traditional medicines.

STRATEGIES FOR THE DEVELOPMENT OF TRADITIONAL MEDICINE BASED ON THE PAN-GPCR DRUG DISCOVERY PLATFORM

The conventional workflow for the development of traditional medicine begins with the extraction and isolation of compounds. Obtaining active components from crude extracts involves iteratively enriching the biologically active molecule through fractionation, guided by *in vitro* cellular assays until the targeted purity and biological activity are achieved (Figure 5A). This procedure often entails not only high costs but also substantial time investment. Current GPCR drug

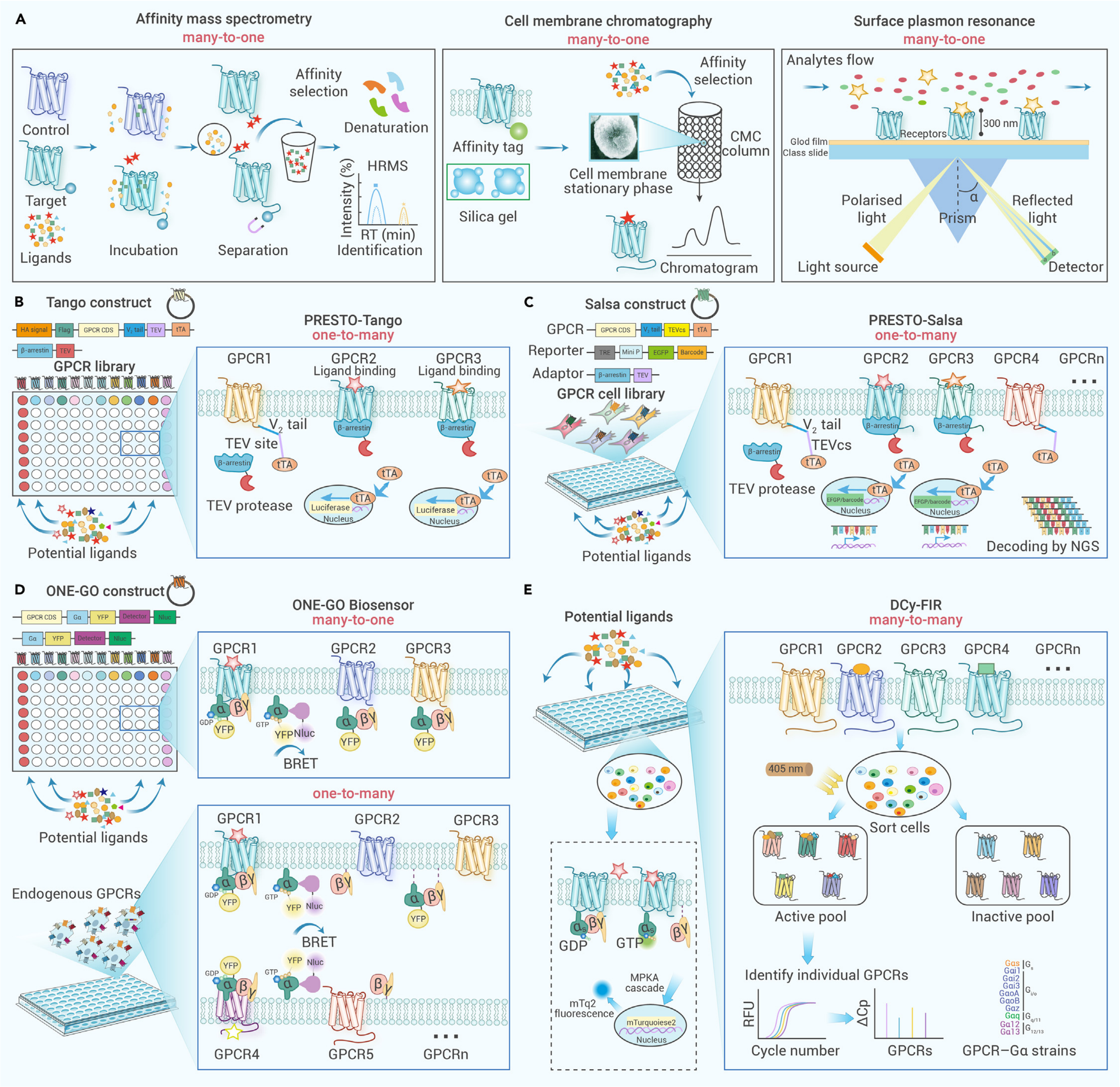

Figure 4. Cell-based ligand screening approaches against GPCRome (A) HTS through ligand binding assays, including SPR, CMC, and A-MS. In the many-to-one screening assays, the receptor is incubated with several ligands simultaneously. Ligand-receptor interactions are identified via chromatography or mass spectrometry. (B and C) One-to-many GPCR ligand screening through PRESTO-Tango (B) and PRESTO-Salsa (C) assays. Each receptor is linked to a unique DNA barcode, and the distinct transcription levels of the corresponding barcode can be measured by RNA sequencing to reflect GPCR activation. (D) Many-to-one or one-to-many GPCR ligand screening based on ONE-GO biosensor. The cell lines expressing different receptors are segregated into separate wells, allowing for independent assessment of their ligand-binding abilities. (E) Many-to-many ligand screening using DCy-FIR assay. The different receptors are directly integrated into the yeast genome using CRISPR technology, enabling the decoding of ligand specificity by examining a mixture of GPCR barcode yeast strains.

discovery often involves HTS only for specific targets, whereas traditional medicines are believed to act simultaneously on multiple targets. Moreover, novel biased, and allosteric GPCR ligands require alternative screening methods with greater sensitivity and throughput. In this context, we proposed a genome-wide pan-GPCR drug discovery platform that comprises three key parts: GPCRome resources supporting HTS *in vitro* and *in silico*, HTS systems from many-to-many to one-to-one assays, and pharmacological assessments from genotype to phenotype (Figure 5B). This platform was designed for GPCR ligand screening of traditional medicines against multiple targets simultaneously, and thus holds promise for elucidating their clinical roles, harnessing their synergistic effects, and developing new drugs from them. The following sections elaborate on these concepts.

Establishment of the GPCRome cell library supporting pan-GPCR HTS

The measurement of GPCR responses induced by traditional medicines may pose challenges, primarily because of three variables: the presence of diverse components acting as ligands, the involvement of multiple synergistic receptors, and the promiscuous activation of distinct signaling pathways. However, these

www.the-innovation.org

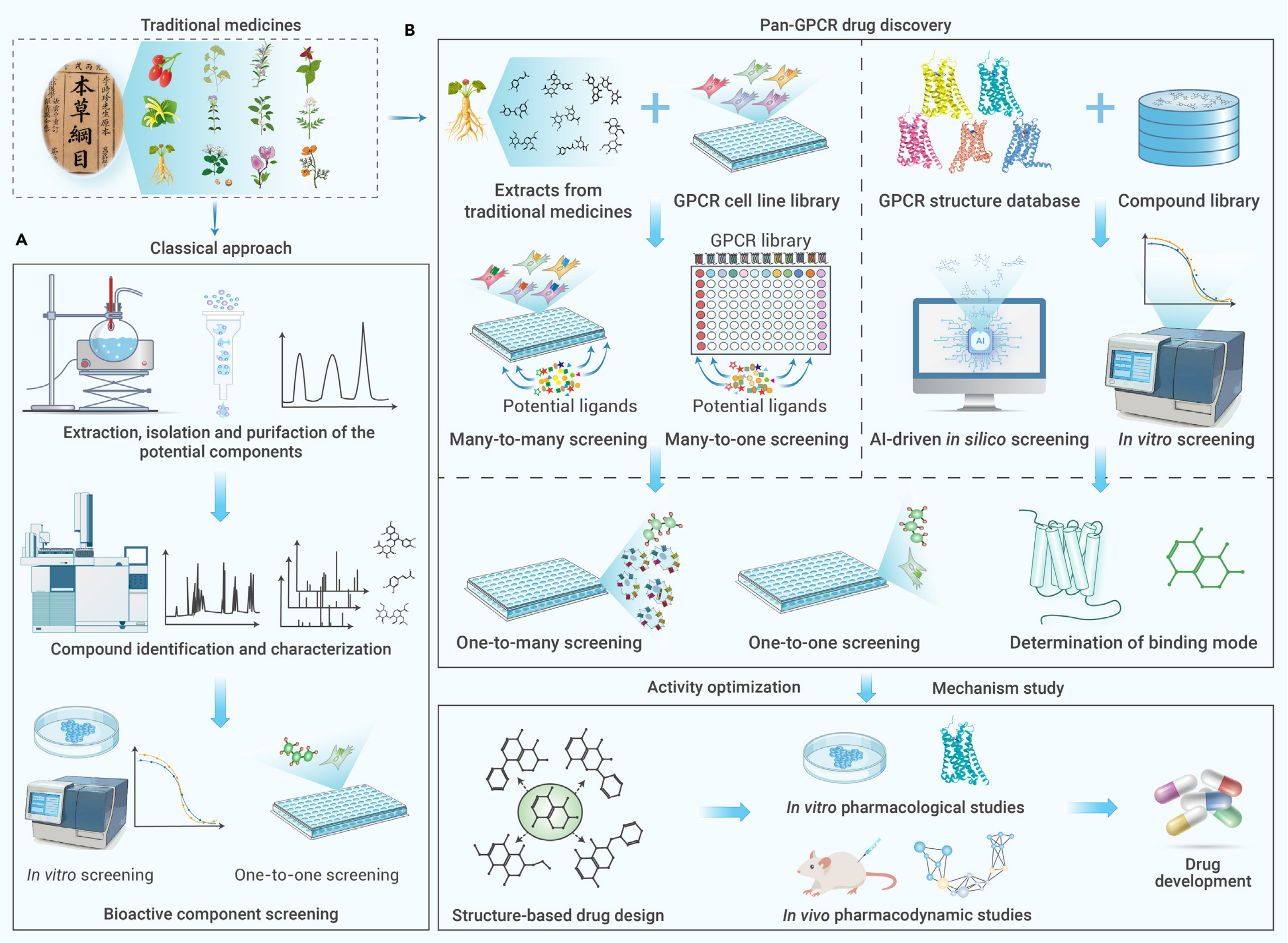

Figure 5. Strategies for traditional medicine development based on the pan-GPCR drug discovery platform (A) Conventional approach of GPCR ligand discovery from traditional medicines involves the following steps: (1) extraction, isolation, and purification of compounds derived from traditional medicines; (2) identification and characterization of the compounds by determining their structures; (3) evaluation and preliminary screening through *in vitro* biological assays, typically using a one-to-one screening strategy exemplified by techniques such as FRET, BRET, NanoBiT, and FLIPR. (B) Pan-GPCR drug discovery is mainly carried out in two ways. (1) Initially, the ligand screening can be conducted from many-to-many (DCy-FIR), many-to-one (PRESTO-Tango, ONE-GO biosensor, CMC, A-MS), one-to-many (ONE-GO biosensor), or one-to-one (FRET, BRET, NanoBiT, FLIPR, etc.) screening, followed by construction of a GPCRome cell line library. (2) AI-driven *in silico* screening can be performed using the GPCRome structure database and virtual natural compound library. The potential ligands are further validated by *in vitro* one-to-one or many-to-one screening and binding mode determination; the potential compounds are modified according to binding modes and optimized to improve pharmacological activity.

factors were not systematically determined based on a unified readout. Although conventional functional assays that measure indicators like second messenger signaling, β-arrestin recruitment, and G protein coupling are highly valuable, they often demand significant resources and can be prohibitively expensive due to the large quantities of transfected cells and ligands required. The pan-GPCR HTS platform aims to overcome these limitations by simultaneously involving multiple targets. Although a wide range of GPCR assays is available, we emphasize three specific assays targeting the GPCRome: ONE-GO biosensor, DCy-FIR, and PRESTO-Salsa. Combining these screening approaches with other conventional methods has the potential to advance GPCR drug discovery, particularly in the field of traditional medicine development.

We have recently established a genome-wide cell library to screen nearly all druggable GPCRs in a simultaneous and parallel manner.[149] The GPCRome cell lines are engineered to overexpress a specific GPCR or a set of GPCRs of interest, which offers a consistent and reproducible system for HTS assays. These cell lines are amenable to automation and can be cultured in 96-well, 384-well, or even 1,536-well plates, allowing the simultaneous screening of millions of compounds or extracts from traditional medicines. Since all cell lines express the receptor at similar levels, the assays are more uniform across different wells and plates, enhancing the reliability of the screening process. Stable cell lines also make the detection of potential ligand-receptor interactions in the crude extracts of traditional medicines via CMC and A-MS readily available. By providing an efficient and scalable screening approach that targets a wide range of druggable GPCRs, the use of GPCRome cell lines in HTS could accelerate the early stages of drug development.

Establishment of the GPCRome structure database supporting *in silico* screening

Recent progress in protein engineering and cryo-EM has significantly expanded the repository of GPCR structures available in the Protein DataBank (PDB, https://www.rcsb.org). Until August 2023, according to the GPCR database (http://gpcrdb.org), there are, in total, 1,160 reported structures for 187 unique receptors in various conformations. In addition, the rapidly advancing artificial intelligence (AI) technology presents unparalleled opportunities for data-driven experimental design and drug discovery.[150] The *in silico* approach, known as computational docking, leverages chemical libraries based on natural products, such as the Natural Products Atlas, Dictionary of Natural Products, TCM Database, and ChEMBL. This enables an exponential expansion in the diversity of ligand screening, from thousands to hundreds of millions of GPCR interactions.

In recent years, significant advancements in protein structure prediction have been driven primarily by machine learning (ML)-based modeling. AlphaFold

stands out as a pioneering tool and is capable of predicting the structure of hundreds of millions of proteins. The latest version, AlphaFold3, has the potential to address the complex challenges associated with GPCR structure prediction, such as enhanced accuracy in complex structures, improved prediction of dynamic states, and better prediction of post-translational modifications.[151] The applications of ML span various fields, including pharmaceutical development, bioactivity prediction, novel molecule generation, and the analysis of biological images. Advancements in any of these areas will significantly propel our understanding of GPCR biology and present a transformative potential in structural research, unquestionably underscoring the promise of AI-driven drug discovery.[152] All GPCR structures in various conformational states can be obtained from available databases (eg, PDB) or can be predicted by AlphaFold3, thus enabling the establishment of a genome-wide structure database for the GPCRome.[153] Given the target uncertainty of most traditional medicines, an initially comprehensive and indiscriminate screening is necessary to identify their interactions with specific GPCRs, which can only be achieved using the GPCRome structure database.

Discovery of GPCR ligands from traditional medicines

Instead of exclusively profiling single targeted affinities, the pan-GPCR platform strives to identify multiple targeted hits based on various readouts. Once the active chemical compounds have been identified, GPCR profiling of the potential components expands beyond target-based hit identification to simultaneously support target validation. The ligand-receptor interaction will differentiate lead molecules by SAR studies and further filter them by *in vitro* or *in vivo* efficacy studies. The advent of libraries of drug-like compounds from traditional medicines has significantly broadened the possibility of HTS, enhancing both the diversity and quality of chemotypes. By combining the DCy-FIR or PRESTO-Salsa platforms with other GPCR screening approaches, traditional medicine components can be rapidly explored through the identification of receptor-ligand interactions. The binding ability of candidate components can be validated using A-MS, CMC, or CLBA. Subsequently, hit compounds can undergo further validation of their pharmacological properties, including analysis of binding affinity and functional responses mediated by GPCRs.

Understanding the structural differences between distinct ligand subtypes and the characteristics of the ligand-binding sites will enable the design of molecules with greater specificity and fewer side effects. Recent advancements in cryo-EM have enabled the discovery of novel allosteric binding sites that were previously challenging to define.[154] Determining the structures of GPCRs bound to traditional medicine-derived molecules is likely to provide conclusive evidence of their binding modes, thus providing theoretical guidance for further drug development. Molecular insights into how ligands interact with crucial residues and stabilize specific receptor conformations will facilitate the design of drugs with functional selectivity. Furthermore, the chemical structures of natural ligands exhibit remarkable diversity, suggesting that nature provides structures that may not be easily attainable through drug discovery efforts in the laboratory. Examples include caffeine and theophylline binding to $A_{2A}R$ and morphine binding to MOR.[30,116] It is hoped that traditional medicines will continue to offer valuable insights into the action of GPCRs and potential therapeutic options provided thereby.

Mechanism studies of phenotypic expression of genotype

In the context of GPCR research, the path from genotype to phenotype encompasses a vast array of molecular interactions, signaling mechanisms, and biological outcomes. The culmination of GPCR signaling pathways results in discernible physiological responses, encompassing alterations in metabolism, cellular proliferation, immune reactions, neural transmission, and other related phenomena. These responses constitute a phenotypic manifestation of the original genetic information that encodes GPCRs. Based on established *in vivo* and *in vitro* models of diseases, the effects of the screened compounds derived from traditional medicines will be evaluated at the cellular and individual levels using various pharmacodynamic indicators.

Genetic variations in GPCRs can affect drug responses. GPCRome cell lines can be designed to express GPCRs with naturally occurring mutations, thereby enabling studies on the influence of genetic variations on drug efficacy and safety. In addition, some GPCRs do not function in isolation and often form homodimers, heterodimers, or larger complexes with other receptors or accessory proteins. By using GPCRome cell lines co-expressing different receptor combinations, researchers are able to explore how GPCR interactions influence signaling pathways and phenotypic outcomes. Combined multiomics analysis is a crucial step in the subsequent analysis of the pharmacodynamic effects and targets of candidate compounds. For example, transcriptomics contributes to the differential analysis of gene expression profiles between pre- and post-drug treatment conditions, proteomics enhances the comprehension of expression patterns and post-translational modifications after drug administration, and metabolomics can effectively elucidate the effects of drugs on metabolic pathways. These approaches contribute to the verification of whether a candidate compound acts through the expected GPCR, while also potentially revealing unknown indirect targets and pathways. The preceding steps aimed to elucidate the pharmacodynamic material basis and targets of traditional medicines, thereby facilitating the exploration of the mechanisms underlying their clinical efficacy.

POTENTIAL APPLICATION OF THE PAN-GPCR DRUG DISCOVERY PLATFORM

The GPCRome resources would offer a promising GPCR-centric trajectory for unveiling novel molecular skeletons from traditional medicines, identifying ligands with diverse binding sites or functional selectivity, facilitating in-depth functional studies, reinforcing the efficacy of pharmaceutical development, and unraveling the therapeutic mechanisms of traditional medicines. Compared with traditional pharmacodynamic studies, ligand screening based on genome-wide GPCR cell libraries is more cost effective and time saving.

Discovery of novel molecular skeletons from traditional medicines

Traditional medicine offers unique therapeutic benefits that cannot be replicated by modern medical treatments. Identifying the active ingredients in traditional medicines can substantially decrease costs and accelerate the timeline of drug development, as the safety and efficacy of traditional medicines have already been verified through their long-standing use. Although the advent of AI-driven structure prediction and drug design signifies a new era, experiment-based molecular discovery has traditionally played a crucial role in the initial stages of drug development and remains the sole approach for uncovering novel molecules from natural products. The genome-wide pan-GPCR cell library facilitates the exploration of interactions between the GPCRome and molecules from traditional medicines and the identification of potential GPCR ligands as agonists, antagonists, or allosteric modulators. Promising molecules would undergo further optimization to enhance their potency, specificity, and pharmacokinetic properties.

Discovery of GPCR ligands with diverse binding sites and functional profiles

Historically, GPCR drug discovery has mainly focused on classical orthosteric modulation through agonist, antagonist, or inverse agonist. However, with an improved understanding of GPCR physiology, new categories of ligands with advantages over conventional orthosteric ligands are emerging. These include allosteric modulators that regulate the activity of orthosteric ligands, biased ligands that selectively activate specific signaling pathways, and dualsteric/bitopic ligands that interact with both orthosteric and allosteric sites. Nonetheless, existing GPCR screening methodologies pose substantial challenges in assessing these novel ligand types, particularly in the context of traditional medicine. To our knowledge, among all the herb-derived GPCR ligands, only AA, which was identified as a toxic ingredient, has been structurally demonstrated as an allosteric modulator (Figure 3E). Thus, relying solely on a single readout in screening may result in overlooking potential therapeutic agents that exhibit biased signaling or allosteric binding properties. GPCRome cell lines can be used to integrate several pharmacological studies, such as signal profiling and omics analyses, thereby incorporating desirable properties at an early stage of hit identification.

Facilitating in-depth functional study of druggable GPCRs

Despite the widespread recognition of more than 300 human GPCRs as drug targets, a significant proportion of them remain poorly understood. GPCRome analyses have uncovered elevated mRNA levels in certain disease contexts, highlighting new GPCRs as potential therapeutic targets.[145] Integrating omics data with functional validation studies could pave the way for discovering

disease-specific GPCR targets. Engineered to replicate disease-specific profiles, GPCRome cell lines can serve as models for investigating the pathophysiology of specific receptors, thereby validating the therapeutic efficacy of drug molecules. Moreover, the exploration of exogenous ligands derived from traditional medicines that may act on orphan GPCRs has the potential to reveal innovative therapeutic targets.

Reinforcing the efficacy of drug development

Previous analyses have identified a noticeable trend of increased failure rates in the later stages of GPCR drug development, primarily attributed to the adverse drug reactions observed during clinical phases 2 and 3.[155] For example, Lotiglipron, known chemically as PF-07081532, represents a significant breakthrough in the field of GLP-1R agonists, particularly for its efficacy in managing type 2 diabetes; however, phase 2 clinical trials revealed instances of elevated liver enzymes, raising concerns about potential liver toxicity, which led to the discontinuation of the drug development.[156] Given the long history of use in humans, ligands from traditional medicines may provide reassurance for favorable safety and activity profiles.

The current era has witnessed a rapid expansion in GPCR research and drug discovery, primarily propelled by the structural and biochemical technologies discussed herein. Molecular insights into how ligands interact with crucial residues will enable the design of more effective subtype-selective or signaling-biased modulators. Investigating the structures of GPCR in complex with traditional medicine-derived molecules that are suspected to engage with allosteric binding sites will likely yield definitive insights into their special therapeutic mechanisms. Although the genome-wide pan-GPCR drug discovery platform holds promising application prospects in screening GPCR ligands from traditional medicines, it also faces several challenges, such as the absence of well-organized compound libraries, limited understanding of the physiological functions of many GPCRs (especially orphans and olfactory receptors), and a low abundance of active components in traditional medicines, thereby complicating selectivity and specificity determination.

CONCLUSION

Traditional medicines present unique advantages in the realm of drug development due to their extensive clinical use and a wide array of biological activities.[157,158] Screening of active ingredients within traditional medicines has the potential of significantly lowering costs and shortening timelines compared with the *de novo* discovery of drug molecules, since their safety and efficacy have already been verified to some extent through their long-standing use. HTS is important for discovering new chemical leads; however, off-the-shelf techniques have a limited capacity to thoroughly investigate the GPCRome, leaving a significant portion of the GPCR landscape unexplored. In addition, the intricate composition and multifaceted pharmacodynamics of traditional medicines, characterized by multi-target and multi-pathway effects, pose challenges for the development of new drugs.

This review sheds light on the existing functional and structural analyses of therapeutic agents derived from traditional medicines. The genome-wide pan-GPCR drug discovery platform offers several different research perspectives. This strategy can not only discover the targets and mechanisms of traditional medicines' known efficacy but also allows the exploration of their potential indications and adverse effects. From the perspective of disease targets, this strategy could identify potentially druggable GPCRs and would provide guidance for the development of new drugs. In conclusion, the pan-GPCR drug discovery platform represents a promising strategy that merges traditional medicinal systems with contemporary drug discovery approaches and has the potential to unveil novel therapeutic agents with superior efficacy and safety profiles.

REFERENCES

1. Ngo, L.T., Okogun, J.I. and Folk, W.R. (2013). 21st Century natural product research and drug development and traditional medicines. *Nat. Prod. Rep.* **30**:584–592.
2. Call for consultation: Draft Traditional Medicine Strategy 2025–2034. https://www.who.int/news-room/articles-detail/call-for-consultation–draft-traditional-medicine-strategy-2025-2034.
3. Entzeroth, M., Flotow, H. and Condron, P. (2009). Overview of High-Throughput Screening. *CP Pharmacology* **9**:9.4.4.1–9.4.27.
4. Mayr, L.M. and Bojanic, D. (2009). Novel trends in high-throughput screening. *Curr. Opin. Pharmacol.* **9**:580–588.
5. Yasi, E.A., Kruyer, N.S. and Peralta-Yahya, P. (2020). Advances in G protein-coupled receptor high-throughput screening. *Curr. Opin. Biotechnol.* **64**:210–217.
6. Chen, W., Song, C., Leng, L. et al. (2024). The Application of Artificial Intelligence Accelerates G Protein-Coupled Receptor Ligand Discovery. *Engineering* **32**:18–28.
7. Chan, H.C.S., Li, Y., Dahoun, T. et al. (2019). New Binding Sites, New Opportunities for GPCR Drug Discovery. *Trends Biochem. Sci.* **44**:312–330.
8. Hilger, D., Masureel, M. and Kobilka, B.K. (2018). Structure and dynamics of GPCR signaling complexes. *Nat. Struct. Mol. Biol.* **25**:4–12.
9. Leysen, H., Walter, D., Christiaenssen, B. et al. (2021). GPCRs Are Optimal Regulators of Complex Biological Systems and Orchestrate the Interface between Health and Disease. *Int. J. Mol. Sci.* **22**:13387.
10. Riemekasten, G. (2019). Loss of balance in normal GPCR-mediated cell trafficking. *Front. Biosci.* **24**:18–34.
11. Watkins, L.R. and Orlandi, C. (2020). Orphan G Protein Coupled Receptors in Affective Disorders. *Genes* **11**:694.
12. Rothman, R.B., Vu, N., Partilla, J.S. et al. (2003). In Vitro Characterization of Ephedrine-Related Stereoisomers at Biogenic Amine Transporters and the Receptorome Reveals Selective Actions as Norepinephrine Transporter Substrates. *J. Pharmacol. Exp. Therapeut.* **307**:138–145.
13. Dong, L., Luo, Y., Cheng, B. et al. (2013). Bioactivity-integrated ultra-performance liquid chromatography/quadrupole time-of-flight mass spectrometry for the identification of nuclear factor-κB inhibitors and β_2 adrenergic receptor agonists in Chinese medicinal preparation Chuanbeipipa dropping pills. *Biomed. Chromatogr.* **27**:960–967.
14. Zhu, Y., Wu, L., Zhao, Y. et al. (2022). Discovery of oridonin as a novel agonist for BRS-3. *Phytomedicine* **100**:154085.
15. Pirintsos, S., Panagiotopoulos, A., Bariotakis, M. et al. (2022). From Traditional Ethnopharmacology to Modern Natural Drug Discovery: A Methodology Discussion and Specific Examples. *Molecules* **27**:4060.
16. Labani, N., Gbahou, F., Lian, S. et al. (2024). 2023 Julius Axelrod Symposium: Plant-derived molecules acting on GPCRs. *Mol. Pharmacol.* **105**:328–347.
17. Qiao, L., Huang, W., Zhang, X. et al. (2021). Evaluation of the immunomodulatory effects of anti-COVID-19 TCM formulae by multiple virus-related pathways. *Signal Transduct. Targeted Ther.* **6**:50.
18. Wong, T.-S., Li, G., Li, S. et al. (2023). G protein-coupled receptors in neurodegenerative diseases and psychiatric disorders. *Signal Transduct. Targeted Ther.* **8**:177.
19. Wang, B., Wang, M., Ao, D. et al. (2021). An Insight into GPCR and G-Proteins as Cancer Drivers. *Cells* **10**:3288.
20. Riddy, D.M., Delerive, P., Summers, R.J. et al. (2018). G Protein–Coupled Receptors Targeting Insulin Resistance, Obesity, and Type 2 Diabetes Mellitus. *Pharmacol. Rev.* **70**:39–67.
21. Zhang, T., An, W., You, S. et al. (2024). G protein-coupled receptors and traditional Chinese medicine: new thinks for the development of traditional Chinese medicine. *Chin. Med.* **19**:92.
22. White, N.J. (1996). The Treatment of Malaria. *N. Engl. J. Med.* **335**:800–806.
23. Dou, J., Ilina, P., Cruz, C.D. et al. (2023). Willow Bark-Derived Material with Antibacterial and Antibiofilm Properties for Potential Wound Dressing Applications. *J. Agric. Food Chem.* **71**:16554–16567.
24. Mackowiak, P.A. (2000). Brief History of Antipyretic Therapy. *Clin. Infect. Dis.* **31**:S154–S156.
25. Kumar, S., Singh, B. and Singh, R. (2022). Catharanthus roseus (L.) G. Don: A review of its ethnobotany, phytochemistry, ethnopharmacology and toxicities. *J. Ethnopharmacol.* **284**:114647.
26. Lu, M., Zhang, L., Pan, J. et al. (2023). Advances in the study of the vascular protective effects and molecular mechanisms of hawthorn (*Crataegus anamesa* Sarg.) extracts in cardiovascular diseases. *Food Funct.* **14**:5870–5890.
27. Li, W., Wu, Z., Xia, Y. et al. (2022). Antiviral and Antioxidant Components from the Fruits of *Illicium verum* Hook.f. (Chinese Star Anise). *J. Agric. Food Chem.* **70**:3697–3707.
28. Newman, D.J. and Cragg, G.M. (2020). Natural Products as Sources of New Drugs over the Nearly Four Decades from 01/1981 to 09/2019. *J. Nat. Prod.* **83**:770–803. DOI:https://doi.org/10.1021/acs.jnatprod.9b0128.
29. Muratspahić, E., Freissmuth, M. and Gruber, C.W. (2019). Nature-Derived Peptides: A Growing Niche for GPCR Ligand Discovery. *Trends Pharmacol. Sci.* **40**:309–326.
30. Zhuang, Y., Wang, Y., He, B. et al. (2022). Molecular recognition of morphine and fentanyl by the human μ-opioid receptor. *Cell* **185**:4361–4375.e19.
31. Lyu, S., Zhang, X., Tu, Z. et al. (2022). GPR108 is required for gambogic acid inhibiting NF-κB signaling in cancer. *Pharmacol. Res.* **182**:106279.
32. Jiang, X., Chen, S., Zhang, Q. et al. (2020). Celastrol is a novel selective agonist of cannabinoid receptor 2 with anti-inflammatory and anti-fibrotic activity in a mouse model of systemic sclerosis. *Phytomedicine* **67**:153160.
33. Koehbach, J., O'Brien, M., Muttenthaler, M. et al. (2013). Oxytocic plant cyclotides as templates for peptide G protein-coupled receptor ligand design. *Proc. Natl. Acad. Sci. USA* **110**:21183–21188.
34. Loor, F., Tiberghien, F., Wenandy, T. et al. (2002). Cyclosporins: Structure–Activity Relationships for the Inhibition of the Human *FPR1* Formylpeptide Receptor. *Journal of Medicinal Chemistry* **45**:4613–4628.
35. Nielsen, L.L., Young, A.A. and Parkes, D.G. (2004). Pharmacology of exenatide (synthetic exendin-4): a potential therapeutic for improved glycemic control of type 2 diabetes. *Regul. Pept.* **117**:77–88.

36. Yanguas-Casás, N., Barreda-Manso, M.A., Nieto-Sampedro, M. et al. (2017). TUDCA: An Agonist of the Bile Acid Receptor GPBAR1/TGR5 With Anti-Inflammatory Effects in Microglial Cells. *Journal Cellular Physiology* **232**:2231–2245.
37. Huang, F., Mariani, N., Pariante, C.M. et al. (2023). From dried bear bile to molecular investigation of differential effects of bile acids in ex vivo and in vitro models of myocardial dysfunction: Relevance for neuroinflammation. *Brain Behav. Immun. Health* **32**:100674.
38. Guo, S., Zhao, T., Yun, Y. et al. (2022). Recent progress in assays for GPCR drug discovery. *Am. J. Physiol. Cell Physiol.* **323**:C583–C594.
39. Flanagan, C.A. (2016). Chapter 10 - GPCR-radioligand binding assays. In Methods in Cell Biology, A.K. Shukla, ed. (Academic Press), pp. 191–215.
40. Bouma, J., Broekhuis, J.D., van der Horst, C. et al. (2023). Dual allosteric and orthosteric pharmacology of synthetic analog cannabidiol-dimethylheptyl, but not cannabidiol, on the cannabinoid CB2 receptor. *Biochem. Pharmacol.* **218**:115924.
41. DeLapp, N.W. (2004). The antibody-capture [^{35}S]GTPγS scintillation proximity assay: a powerful emerging technique for analysis of GPCR pharmacology. *Trends Pharmacol. Sci.* **25**:400–401.
42. Stoddart, L.A., White, C.W., Nguyen, K. et al. (2016). Fluorescence- and bioluminescence-based approaches to study GPCR ligand binding. *Br. J. Pharmacol.* **173**:3028–3037.
43. Lee, P.H. (2009). Label-free optical biosensor: A tool for G protein-coupled receptors pharmacology profiling and inverse agonists identification. *J. Recept. Signal Transduct. Res.* **29**:146–153.
44. Nonobe, Y., Yokoyama, T., Kamikubo, Y. et al. (2016). Application of surface plasmon resonance imaging to monitoring G protein-coupled receptor signaling and its modulation in a heterologous expression system. *BMC Biotechnol.* **16**:36.
45. Algar, W.R., Hildebrandt, N., Vogel, S.S. et al. (2019). FRET as a biomolecular research tool — understanding its potential while avoiding pitfalls. *Nat. Methods* **16**:815–829.
46. Pfleger, K.D.G., Seeber, R.M. and Eidne, K.A. (2006). Bioluminescence resonance energy transfer (BRET) for the real-time detection of protein-protein interactions. *Nat. Protoc.* **1**:337–345.
47. Dixon, A.S., Schwinn, M.K., Hall, M.P. et al. (2016). NanoLuc Complementation Reporter Optimized for Accurate Measurement of Protein Interactions in Cells. *ACS Chem. Biol.* **11**:400–408.
48. Jiang, H., Galtes, D., Wang, J. et al. (2022). G protein-coupled receptor signaling: transducers and effectors. *Am. J. Physiol. Cell Physiol.* **323**:C731–C748.
49. Olsen, R.H.J., DiBerto, J.F., English, J.G. et al. (2020). TRUPATH, an open-source biosensor platform for interrogating the GPCR transducerome. *Nat. Chem. Biol.* **16**:841–849.
50. Nishi, A., Yamaguchi, T., Sekiguchi, K. et al. (2012). Geissoschizine methyl ether, an alkaloid in Uncaria hook, is a potent serotonin1A receptor agonist and candidate for amelioration of aggressiveness and sociality by yokukansan. *Neuroscience* **207**:124–136.
51. Rajagopal, S. and Shenoy, S.K. (2018). GPCR desensitization: Acute and prolonged phases. *Cell. Signal.* **41**:9–16.
52. Kim, K. and Chung, K.Y. (2020). Many faces of the GPCR-arrestin interaction. *Arch Pharm. Res. (Seoul)* **43**:890–899.
53. Gurevich, V.V. and Gurevich, E.V. (2020). Biased GPCR signaling: Possible mechanisms and inherent limitations. *Pharmacol. Ther.* **211**:107540.
54. Barnea, G., Strapps, W., Herrada, G. et al. (2008). The genetic design of signaling cascades to record receptor activation. *Proc. Natl. Acad. Sci. USA* **105**:64–69.
55. Kopra, K., Kainulainen, M., Mikkonen, P. et al. (2013). Multiparametric Homogeneous Method for Identification of Ligand Binding to G Protein-Coupled Receptors: Receptor–Ligand Binding and β-Arrestin Assay. *Anal. Chem.* **85**:2276–2281.
56. Kroeze, W.K., Sassano, M.F., Huang, X.-P. et al. (2015). PRESTO-Tango as an open-source resource for interrogation of the druggable human GPCRome. *Nat. Struct. Mol. Biol.* **22**:362–369.
57. Li, H., Wang, F., Zhou, Z. et al. (2022). Atractylon, a novel dopamine 2 receptor agonist, ameliorates Parkinsonian like motor dysfunctions in MPTP-induced mice. *Neurotoxicology* **89**:121–126.
58. Masuho, I., Kise, R., Gainza, P. et al. (2023). Rules and mechanisms governing G protein coupling selectivity of GPCRs. *Cell Rep.* **42**:113173.
59. Seong, S.H., Kim, S.H., Ryu, J.H. et al. (2023). Effects of Icariin and Its Metabolites on GPCR Regulation and MK-801-Induced Schizophrenia-Like Behaviors in Mice. *Molecules* **28**:7300.
60. Cheng, Z., Garvin, D., Paguio, A. et al. (2010). Luciferase Reporter Assay System for Deciphering GPCR Pathways. *Curr. Chem. Genom.* **4**:84–91.
61. Rukoyatkina, N., Shpakova, V., Bogoutdinova, A. et al. (2022). Curcumin by activation of adenosine A2A receptor stimulates protein kinase a and potentiates inhibitory effect of cangrelor on platelets. *Biochem. Biophys. Res. Commun.* **586**:20–26.
62. Hamilton, L.J., Walker, M., Pattabiraman, M. et al. (2021). Novel curcumin analog (cis-trans curcumin) as ligand to adenosine receptors A_{2A} and A_{2B}: potential for therapeutics. *Pharmacol. Res.* **165**:105410.
63. Jia, T., Rao, J., Zou, L. et al. (2017). Nanoparticle-Encapsulated Curcumin Inhibits Diabetic Neuropathic Pain Involving the P2Y12 Receptor in the Dorsal Root Ganglia. *Front. Neurosci.* **11**:755.
64. Harada, N., Okuyama, M., Teraoka, Y. et al. (2022). Identification of G protein-coupled receptor 55 (GPR55) as a target of curcumin. *NPJ Sci. Food* **6**:4.
65. Harada, N., Arahori, Y., Okuyama, M. et al. (2022). Curcumin activates G protein-coupled receptor 97 (GPR97) in a manner different from glucocorticoid. *Biochem. Biophys. Res. Commun.* **595**:41–46.
66. Kato, M., Nishikawa, S., Ikehata, A. et al. (2017). Curcumin improves glucose tolerance via stimulation of glucagon-like peptide-1 secretion. *Mol. Nutr. Food Res.* **61**:1600471.
67. Urošević, M., Nikolić, L., Gajić, I. et al. (2022). Curcumin: Biological Activities and Modern Pharmaceutical Forms. *Antibiotics* **11**:135.
68. Callahan, B.N., Kammala, A.K., Syed, M. et al. (2020). Osthole, a Natural Plant Derivative Inhibits MRGPRX2 Induced Mast Cell Responses. *Front. Immunol.* **11**:703.
69. Chern, C.-M., Zhou, H., Wang, Y.-H. et al. (2020). Osthole ameliorates cartilage degradation by downregulation of NF-κB and HIF-2α pathways in an osteoarthritis murine model. *Eur. J. Pharmacol.* **867**:172799.
70. Liang, H.-J., Suk, F.-M., Wang, C.-K. et al. (2009). Osthole, a potential antidiabetic agent, alleviates hyperglycemia in db/db mice. *Chem. Biol. Interact.* **181**:309–315.
71. Wang, J., Fu, Y., Wei, Z. et al. (2017). Anti-asthmatic activity of osthole in an ovalbumin-induced asthma murine model. *Respir. Physiol. Neurobiol.* **239**:64–69.
72. Shokoohinia, Y., Jafari, F., Mohammadi, Z. et al. (2018). Potential Anticancer Properties of Osthol: A Comprehensive Mechanistic Review. *Nutrients* **10**:36.
73. Ogasawara, H. and Noguchi, M. (2021). Therapeutic Potential of MRGPRX2 Inhibitors on Mast Cells. *Cells* **10**:2906.
74. Hwang, S.H., Shin, T.-J., Choi, S.-H. et al. (2012). Gintonin, Newly Identified Compounds from Ginseng, Is Novel Lysophosphatidic Acids-Protein Complexes and Activates G Protein-Coupled Lysophosphatidic Acid Receptors with High Affinity. *Mol. Cell.* **33**:151–162.
75. Cho, Y.-J., Choi, S.-H., Lee, R. et al. (2020). Ginseng Gintonin Contains Ligands for GPR40 and GPR55. *Molecules* **25**:1102.
76. He, J., Fang, J., Wang, Y. et al. (2023). Discovery of Small-Molecule Antagonists of Orexin 1/2 Receptors from Traditional Chinese Medicinal Plants with a Hypnotic Effect. *Pharmaceuticals* **16**:542.
77. Fujita, T., Inoue, K., Yamamoto, S. et al. (1994). Fungal metabolites. Part 11. A potent immunosuppressive activity found in Isaria sinclairii metabolite. *J. Antibiot.* **47**:208–215.
78. Chiba, K. (2005). FTY720, a new class of immunomodulator, inhibits lymphocyte egress from secondary lymphoid tissues and thymus by agonistic activity at sphingosine 1-phosphate receptors. *Pharmacol. Ther.* **108**:308–319.
79. Koch, T. and Höllt, V. (2008). Role of receptor internalization in opioid tolerance and dependence. *Pharmacol. Ther.* **117**:199–206.
80. Cahill, T.J., Thomsen, A.R.B., Tarrasch, J.T. et al. (2017). Distinct conformations of GPCR–β-arrestin complexes mediate desensitization, signaling, and endocytosis. *Proc. Natl. Acad. Sci. USA* **114**:2562–2567.
81. Teixeira da Silva, J.A. and Ng, T.B. (2017). The medicinal and pharmaceutical importance of Dendrobium species. *Appl. Microbiol. Biotechnol.* **101**:2227–2239.
82. Liang, J., Chen, S., Chen, J. et al. (2018). Therapeutic roles of polysaccharides from Dendrobium Officinaleon colitis and its underlying mechanisms. *Carbohydr. Polym.* **185**:159–168.
83. Feng, X.-T., Wang, T.-Z., Chen, Y.I. et al. (2012). Pollen Typhae total flavone improves insulin-induced glucose uptake through the β-arrestin-2-mediated signaling in C2C12 myotubes. *Int. J. Mol. Med.* **30**:914–922.
84. Lefkowitz, R.J. (2013). A Brief History of G-Protein Coupled Receptors (Nobel Lecture). *Angew. Chem., Int. Ed. Engl.* **52**:6366–6378.
85. Ikeda, Y., Kumagai, H., Motozawa, Y. et al. (2015). Biased Agonism of the Angiotensin II Type I Receptor. *Int. Heart J.* **56**:485–488.
86. Wu, M.Y., Ge, Y.J., Wang, E.J. et al. (2023). Enhancement of efferocytosis through biased FPR_2 signaling attenuates intestinal inflammation. *EMBO Mol. Med.* **15**:e17815.
87. Suh, H.-W., Lee, K.-B., Kim, K.-S. et al. (2015). A bitter herbal medicine *Gentiana scabra* root extract stimulates glucagon-like peptide-1 secretion and regulates blood glucose in *db/db* mouse. *J. Ethnopharmacol.* **172**:219–226.
88. King, K., Lin, N.-P., Cheng, Y.-H. et al. (2015). Isolation of Positive Modulator of Glucagon-like Peptide-1 Signaling from *Trigonella foenum-graecum* (Fenugreek) Seed. *J. Biol. Chem.* **290**:26235–26248.
89. Sturrock, E.D., Lubbe, L., Cozier, G.E. et al. (2019). Structural basis for the C-domain-selective angiotensin-converting enzyme inhibition by bradykinin-potentiating peptide b (BPPb). *Biochem. J.* **476**:1553–1570.
90. Lu, D. and Potter, D.E. (2017). Chapter 58 - Cannabinoids and the Cannabinoid Receptors: An Overview. In Handbook of Cannabis and Related Pathologies, V.R. Preedy, ed. (Academic Press), pp. 553–563.
91. Pisanti, S., Malfitano, A.M., Ciaglia, E. et al. (2017). Cannabidiol: State of the art and new challenges for therapeutic applications. *Pharmacol. Ther.* **175**:133–150.
92. Hanuš, L.O., Meyer, S.M., Muñoz, E. et al. (2016). Phytocannabinoids: a unified critical inventory. *Nat. Prod. Rep.* **33**:1357–1392.
93. Di Marzo, V., Melck, D., Bisogno, T. et al. (1998). Endocannabinoids: endogenous cannabinoid receptor ligands with neuromodulatory action. *Trends Neurosci.* **21**:521–528.
94. Howlett, A.C. and Abood, M.E. (2017). CB1 and CB2 Receptor Pharmacology. In Advances in Pharmacology, D. Kendall and S.P.H. Alexander, eds. (Academic Press), pp. 169–206.
95. Krishna Kumar, K., Robertson, M.J., Thadhani, E. et al. (2023). Structural basis for activation of CB1 by an endocannabinoid analog. *Nat. Commun.* **14**:2672.
96. Hua, T., Vemuri, K., Nikas, S.P. et al. (2017). Crystal structures of agonist-bound human cannabinoid receptor CB1. *Nature* **547**:468–471.
97. Leo, L.M. and Abood, M.E. (2021). CB1 Cannabinoid Receptor Signaling and Biased Signaling. *Molecules* **26**:5413.
98. Liao, Y.-Y., Zhang, H., Shen, Q. et al. (2023). Snapshot of the cannabinoid receptor 1-arrestin complex unravels the biased signaling mechanism. *Cell* **186**:5784–5797.e17.
99. Wang, Y., Wu, L., Wang, T. et al. (2024). Cryo-EM structure of cannabinoid receptor CB1-β-arrestin complex. *Protein Cell* **15**:230–234.
100. Hua, T., Li, X., Wu, L. et al. (2020). Activation and Signaling Mechanism Revealed by Cannabinoid Receptor-G_i Complex Structures. *Cell* **180**:655–665.e18.

www.the-innovation.org

101. Vadhel, A., Bashir, S., Mir, A.H. et al. (2023). Opium alkaloids, biosynthesis, pharmacology and association with cancer occurrence. *Open Biol.* **13**:220355.
102. Hill, R. and Canals, M. (2022). Experimental considerations for the assessment of in vivo and in vitro opioid pharmacology. *Pharmacol. Ther.* **230**:107961.
103. Begum, T., Arzmi, M.H., Khatib, A. et al. (2024). A review on *Mitragyna speciosa* (Rubiaceae) as a prominent medicinal plant based on ethnobotany, phytochemistry and pharmacological activities. *Nat. Prod. Res.* **0**:1–17.
104. Váradi, A., Marrone, G.F., Palmer, T.C. et al. (2016). Mitragynine/Corynantheidine Pseudoindoxyls As Opioid Analgesics with Mu Agonism and Delta Antagonism, Which Do Not Recruit β-Arrestin-2. *J. Med. Chem.* **59**:8381–8397.
105. Maxwell, E.A., King, T.I., Kamble, S.H. et al. (2021). Oral Pharmacokinetics in Beagle Dogs of the Mitragynine Metabolite, 7-Hydroxymitragynine. *Eur. J. Drug Metab. Pharmacokinet.* **46**:459–463.
106. Roth, B.L., Baner, K., Westkaemper, R. et al. (2002). Salvinorin A: A potent naturally occurring nonnitrogenous κ opioid selective agonist. *Proc. Natl. Acad. Sci. USA* **99**:11934–11939.
107. Munro, T.A., Ho, D.M. and Cohen, B.M. (2012). Salvinorin B methoxymethyl ether. *Acta Crystallogr.* **68**:o3225–o3226.
108. Han, J., Zhang, J., Nazarova, A.L. et al. (2023). Ligand and G-protein selectivity in the κ-opioid receptor. *Nature* **617**:417–425.
109. Cao, C., Barros-Álvarez, X., Zhang, S. et al. (2022). Signaling snapshots of a serotonin receptor activated by the prototypical psychedelic LSD. *Neuron* **110**:3154–3167.e3157.
110. McClure-Begley, T.D. and Roth, B.L. (2022). The promises and perils of psychedelic pharmacology for psychiatry. *Nat. Rev. Drug Discov.* **21**:463–473.
111. Hofmann, C., Penner, U., Dorow, R. et al. (2006). Lisuride, a Dopamine Receptor Agonist With 5-HT_{2B} Receptor Antagonist Properties. *Clin. Neuropharmacol.* **29**:80–86.
112. Ling, S., Ceban, F., Lui, L.M.W. et al. (2022). Molecular Mechanisms of Psilocybin and Implications for the Treatment of Depression. *CNS Drugs* **36**:17–30.
113. Cao, D., Yu, J., Wang, H. et al. (2022). Structure-based discovery of nonhallucinogenic psychedelic analogs. *Science* **375**:403–411.
114. Wacker, D., Wang, S., McCorvy, J.D. et al. (2017). Crystal Structure of an LSD-Bound Human Serotonin Receptor. *Cell* **168**:377–389.e12.
115. Egan, C.T., Herrick-Davis, K., Miller, K. et al. (1998). Agonist activity of LSD and lisuride at cloned $5HT_{2A}$ and $5HT_{2C}$ receptors. *Psychopharmacology* **136**:409–414.
116. Biaggioni, I., Paul, S., Puckett, A. et al. (1991). Caffeine and theophylline as adenosine receptor antagonists in humans. *J. Pharmacol. Exp. Therapeut.* **258**:588–593.
117. Lebon, G., Warne, T., Edwards, P.C. et al. (2011). Agonist-bound adenosine A_{2A} receptor structures reveal common features of GPCR activation. *Nature* **474**:521–525.
118. Matsunami, H., Montmayeur, J.-P. and Buck, L.B. (2000). A family of candidate taste receptors in human and mouse. *Nature* **404**:601–604.
119. Cui, M., Chen, B., Xu, K. et al. (2021). Activation of specific bitter taste receptors by olive oil phenolics and secoiridoids. *Sci. Rep.* **11**:22340.
120. Ng, C.C., Duke, R.K., Hinton, T. et al. (2017). Effects of bilobalide, ginkgolide B and picrotoxinin on $GABA_A$ receptor modulation by structurally diverse positive modulators. *Eur. J. Pharmacol.* **806**:83–90.
121. Behrens, M., Brockhoff, A., Kuhn, C. et al. (2004). The human taste receptor hTAS2R14 responds to a variety of different bitter compounds. *Biochem. Biophys. Res. Commun.* **319**:479–485.
122. Behrens, M., Brockhoff, A., Batram, C. et al. (2009). The Human Bitter Taste Receptor hTAS2R50 Is Activated by the Two Natural Bitter Terpenoids Andrographolide and Amarogentin. *J. Agric. Food Chem.* **57**:9860–9866.
123. Brockhoff, A., Behrens, M., Massarotti, A. et al. (2007). Broad Tuning of the Human Bitter Taste Receptor hTAS2R46 to Various Sesquiterpene Lactones, Clerodane and Labdane Diterpenoids, Strychnine, and Denatonium. *J. Agric. Food Chem.* **55**:6236–6243.
124. Meyerhof, W., Batram, C., Kuhn, C. et al. (2010). The Molecular Receptive Ranges of Human TAS2R Bitter Taste Receptors. *Chem. Senses* **35**:157–170.
125. Du, J., Lü, W., Wu, S. et al. (2015). Glycine receptor mechanism elucidated by electron cryo-microscopy. *Nature* **526**:224–229.
126. Xu, W., Wu, L., Liu, S. et al. (2022). Structural basis for strychnine activation of human bitter taste receptor TAS2R46. *Science* **377**:1298–1304.
127. Brockhoff, A., Behrens, M., Niv, M.Y. et al. (2010). Structural requirements of bitter taste receptor activation. *Proc. Natl. Acad. Sci. USA* **107**:11110–11115.
128. Hu, X., Ao, W., Gao, M. et al. (2024). Bitter taste TAS2R14 activation by intracellular tastants and cholesterol. *Nature* **631**:459–466.
129. Kim, Y., Gumpper, R.H., Liu, Y. et al. (2024). Bitter taste receptor activation by cholesterol and an intracellular tastant. *Nature* **628**:664–671.
130. Tao, L., Wang, D., Yuan, Q. et al. (2024). Bitter taste receptor TAS2R14 activation and G protein assembly by an intracellular agonist. *Cell Res.* **34**:735–738.
131. Di Pizio, A., Waterloo, L.A.W., Brox, R. et al. (2020). Rational design of agonists for bitter taste receptor TAS2R14: from modeling to bench and back. *Cell. Mol. Life Sci.* **77**:531–542.
132. Wang, D.Q.H. (2014). Therapeutic uses of animal biles in traditional Chinese medicine: An ethnopharmacological, biophysical chemical and medicinal review. *WJG* **20**:9952–9975.
133. Ziegler, F., Steuer, A., Di Pizio, A. et al. (2023). Physiological activation of human and mouse bitter taste receptors by bile acids. *Commun. Biol.* **6**:612.
134. Das, S., Thakur, S., Korenjak, M. et al. (2022). Aristolochic acid-associated cancers: a public health risk in need of global action. *Nat. Rev. Cancer* **22**:576–591.
135. IARC Working Group on the Evaluation of Carcinogenic Risks to Humans (2002). Some traditional herbal medicines, some mycotoxins, naphthalene and styrene. *IARC Monogr. Eval. Carcinog. Risks Hum.* **82**:1–556.
136. Olaru, A., Bala, C., Jaffrezic-Renault, N. et al. (2015). Surface Plasmon Resonance (SPR) Biosensors in Pharmaceutical Analysis. *Crit. Rev. Anal. Chem.* **45**:97–105.
137. Locatelli-Hoops, S., Yeliseev, A.A., Gawrisch, K. et al. (2013). Surface plasmon resonance applied to G protein-coupled receptors. *Biomed. Spectrosc. Imag.* **2**:155–181.
138. Ma, W., Wang, C., Liu, R. et al. (2021). Advances in cell membrane chromatography. *J. Chromatogr. A* **1639**:461916.
139. Ding, Y., Guo, N., Jiang, Y. et al. (2024). Establishment of cluster of differentiation 20 immobilized cell membrane chromatography for the screening of active antitumor components in traditional Chinese medicine. *J. Chromatogr. A* **1721**:464845.
140. Prudent, R., Lemoine, H., Walsh, J. et al. (2023). Affinity selection mass spectrometry speeding drug discovery. *Drug Discov. Today* **28**:103760.
141. Zhang, B., Zhao, S., Yang, D. et al. (2020). A Novel G Protein-Biased and Subtype-Selective Agonist for a G Protein-Coupled Receptor Discovered from Screening Herbal Extracts. *ACS Cent. Sci.* **6**:213–225.
142. Yang, S., Qian, L., Li, Z. et al. (2023). Integrated Multi-Omics Landscape of Liver Metastases. *Gastroenterology* **164**:407–423.e17.
143. Kreitmaier, P., Katsoula, G. and Zeggini, E. (2023). Insights from multi-omics integration in complex disease primary tissues. *Trends Genet.* **39**:46–58.
144. Tao, Y., Xing, S., Zuo, S. et al. (2023). Cell-free multi-omics analysis reveals potential biomarkers in gastrointestinal cancer patients' blood. *Cell Rep. Med.* **4**:101281.
145. Insel, P.A., Sriram, K., Gorr, M.W. et al. (2019). GPCRomics: An Approach to Discover GPCR Drug Targets. *Trends Pharmacol. Sci.* **40**:378–387.
146. Chen, H., Rosen, C.E., González-Hernández, J.A. et al. (2023). Highly multiplexed bioactivity screening reveals human and microbiota metabolome-GPCRome interactions. *Cell* **186**:3095–3110.e3019.
147. Raskovalov, A., Kim, D. and Cherezov, V. (2024). ONE-GO: Direct detection of context-dependent GPCR activity. *Cell Res.* **34**:543–544.
148. Kapolka, N.J., Taghon, G.J., Rowe, J.B. et al. (2020). DCyFIR: a high-throughput CRISPR platform for multiplexed G protein-coupled receptor profiling and ligand discovery. *Proc. Natl. Acad. Sci. USA* **117**:13117–13126.
149. Yang, H., Wang, Y., Liu, W. et al. (2024). Genome-wide pan-GPCR cell libraries accelerate drug discovery. *Acta Pharm. Sin. B* **14**:4296–4311.
150. Xu, Y., Liu, X., Cao, X. et al. (2021). Artificial intelligence: A powerful paradigm for scientific research. *Innovation* **2**:100179.
151. Abramson, J., Adler, J., Dunger, J. et al. (2024). Accurate structure prediction of biomolecular interactions with AlphaFold 3. *Nature* **630**:493–500.
152. Chen, W., Liu, X., Zhang, S. et al. (2023). Artificial intelligence for drug discovery: Resources, methods, and applications. *Mol. Ther. Nucleic Acids* **31**:691–702.
153. Shi, Y. (2024). Drug development in the AI era: AlphaFold 3 is coming! *Innovation* **5**:100685.
154. Zhang, L., Mobbs, J.I., May, L.T. et al. (2023). The impact of cryo-EM on determining allosteric modulator-bound structures of G protein-coupled receptors. *Curr. Opin. Struct. Biol.* **79**:102560.
155. Hauser, A.S., Attwood, M.M., Rask-Andersen, M. et al. (2017). Trends in GPCR drug discovery: new agents, targets and indications. *Nat. Rev. Drug Discov.* **16**:829–842.
156. Buckeridge, C., Tsamandouras, N., Carvajal-Gonzalez, S. et al. (2024). Once-daily oral small-molecule glucagon-like peptide-1 receptor agonist lotiglipron (PF-07081532) for type 2 diabetes and obesity: Two randomized, placebo-controlled, multiple-ascending-dose Phase 1 studies. *Diabetes Obes. Metabol.* **26**:3155–3166.
157. Atanasov, A.G., Waltenberger, B., Pferschy-Wenzig, E.-M. et al. (2015). Discovery and resupply of pharmacologically active plant-derived natural products: A review. *Biotechnol. Adv.* **33**:1582–1614.
158. Chen, S., Li, Z., Zhang, S. et al. (2022). Emerging biotechnology applications in natural product and synthetic pharmaceutical analyses. *Acta Pharm. Sin. B* **12**:4075–4097.

ACKNOWLEDGMENTS

This work was funded by introducing the talented person scientific research starts funds subsidization project of Chengdu University of Traditional Chinese Medicine (030040043, 030040017). The funders had no role in study design, data collection and analysis, decision to publish, or preparation of the manuscript.

AUTHOR CONTRIBUTIONS

Z.B., H.L., Y.L., D. S., S.L., and Z.C. drafted the manuscript. Z.B., H.L., Y.L., and Z.C. proofread the structures, figures, and tables. S.C., W.C., L.L., C.S., and S.Z. provided supervision and revised the manuscript. Z.C. and S.C. approved the final version of the manuscript. All authors contributed to and approved the manuscript.

DECLARATION OF INTERESTS

The authors declare no competing interests.

SUPPLEMENTAL INFORMATION

It can be found online at https://doi.org/10.1016/j.xinn.2024.100774.

The Innovation, Volume *6*

Supplemental Information

Emerging paradigms for target discovery of traditional medicines: A genome-wide pan-GPCR perspective

Zenghao Bi, Huan Li, Yuting Liang, Dan Sun, Songxin Liu, Wei Chen, Liang Leng, Chi Song, Sanyin Zhang, Zhaotong Cong, and Shilin Chen

Emerging Paradigms for Target Discovery of Traditional Medicines: A Genome-Wide Pan-GPCR Perspective

Zenghao Bi,[1,2,3,6] Huan Li,[2,3,4,6] Yuting Liang,[1,2,3,6] Dan Sun,[2,3] Songxin Liu,[2,3,5] Wei Chen,[2,3] Liang Leng,[2,3] Chi Song,[2,3] Sanyin Zhang,[2,3] Zhaotong Cong,[2,3*] Shilin Chen,[2,3]

[1]School of Pharmacy, Chengdu University of Traditional Chinese Medicine, Chengdu 611137, China

[2]Institute of Herbgenomics, Chengdu University of Traditional Chinese Medicine, Chengdu 611137, China

[3]Innovative Institute of Chinese Medicine and Pharmacy, Chengdu University of Traditional Chinese Medicine, Chengdu 611137, China

[4]College of Pharmacy, Nanjing University of Chinese Medicine, Nanjing 210023, China

[5]School of Pharmacy, Shanghai University of Traditional Chinese Medicine, Shanghai 201203, China

[6]These authors contributed equally

*Correspondence: congzt@cdutcm.edu.cn (Z.C.)

Supplemental Information

Table S1. Direct effects of traditional medicines on the GPCR function reported over the last two decades

Targets	Ligands	Natural origin	Signaling pathway	Detection methods	Type of the ligand	Pharmacological efficacy	References
**5-HT$_{1A}$R**	N-methyllaurotetanine	*Eschscholzia californica*	n.d.	CLBA	Agonist	EC_{50} = 155 nM,<br>K_i = 85 nM	1
**5-HT$_{1A}$R**	Geissoschizine methyl ether	*Uncaria hook*	n.d.	CLBA,<br>GTPγS binding assay,<br>*in vivo* activity assays	Agonist	IC_{50} = 0.904 µM (CLBA),<br>K_i = 0.517 µM (CLBA)	2
**5-HT$_{2A}$R**	Psilocybin (Converted to psilocin *in vivo*)	*Psilocybe* genus	n.d.	Positron emission tomography scan	Agonist	EC_{50} = 1.95 µg/L	3,4
**5-HT$_{2C}$R**	(*R*)-asimilobine	*Stephania tetrandra*	G_q,<br>β-arrestin	CLBA,<br>A-MS,<br>Ca^{2+} mobilization,<br>β-arrestin recruitment,<br>molecular docking	Agonist	EC_{50} = 308 nM (Ca^{2+}),<br>*In vivo*: 30 mg/kg	5
**5-HT$_{5A}$R**	Valerenic acid	*Valeriana officinalis*	n.d.	CLBA,<br>GTP shift assay	Partial agonist	K_i = 10.7 µM (CLBA)	6
**A$_1$R**	Isovaltrate	*Valeriana officinalis*	G_i	CLBA,<br>GTPγS binding assay	Inverse agonist	K_i = 2 µM (CLBA),<br>EC_{50} = 4.6 µM (GTPγS)	7
**A$_1$R**	Olivil derivative	*Valeriana officinalis*	G_i	CLBA,<br>GTPγS binding assay,<br>cAMP accumulation	Partial agonist	K_i = 5.28 µM (CLBA)<br>EC_{50} = 3.98 µM (GTPγS),<br>IC_{50} = 15 µM (cAMP)	8

**$A_{2A}R$**	Curcumin	*Curcuma longa*	G_s	Western blot, platelet aggregation	Agonist	Single conc. (5, 10, 50 μM) induced vasodilator-stimulated phosphoprotein phosphorylation	9
**APLNR**	(-)-Epicatechin	*Camellia sinensis*	β-arrestin	β-arrestin recruitment, AKT phosphorylation, molecular docking	Biased agonist	$K_d = 1.755 \times 10^{-12}$ M (β-arrestin)	10,11
**BRS3**	Oridonin	*Isodon rubescens*	G_q	IP1 accumulation, Ca^{2+} mobilization, β-arrestin recruitment, DMR, molecular docking, *in vivo* activity assays	Agonist	EC_{50} = 0.2 μM (Ca^{2+}), EC_{50} = 0.7 μM (IP1), EC_{50} = 1.4 μM (DMR)	12
**CB1**	Genistein	*Genista tinctoria*, *Sophora davidii*	G_i	CLBA, molecular docking, GTP turnover assay, *in vivo* activity assays	Antagonist	IC_{50} = 375 nM (CLBA)	13-15
**CB1**	α-humulene (1), geraniol (2), linalool (3), β-pinene (4)	*Cannabis sativa*	G_i	CLBA, ERK phosphorylation, cAMP accumulation, β-arrestin recruitment, *in vivo* activity assays	Agonist	IC_{50} = 44.2 μM (CLBA, 2)	16
**CB1, CB2**	4-O-methylhonokiol (1), magnolol (2), honokiol (3), tetrahydromagnolol (4)	*Magnolia grandiflora*	n.d.	CLBA, molecular docking	Agonist	CB1: K_i = 3.85 μM (1), 17.82 μM (2), 14.55 μM (3), 19.08 μM (4), CB2: K_i = 0.29 μM (1), 1.40 μM (2), 1.94 μM (3), 0.99 μM (4)	17
**CB2**	Celastrol	*Tripterygium wilfordii*	G_i, β-arrestin	SLCA, β-arrestin recruitment, Ca^{2+} mobilization, cAMP accumulation, ERK phosphorylation, molecular docking	Agonist	EC_{50} = 1.77 μM (SLCA), EC_{50} = 1.81 μM (Ca^{2+}), EC_{50} = 0.99 μM (cAMP)	18,19

**CCK_2R**	Caripe 11 (GVIPCGESCVFIPCISTVIGCSCKKKVCYRN)	*Carapichea ipecacuanha*	G_q	IP1 accumulation	Partial agonist	$EC_{50} = 8.5\ \mu M$	20
**CRF_1R**	Caripe 8 (GVIPCGESCVFIPCITAAIGCSCKKKVCYRN)	*Carapichea ipecacuanha*	G_s	cAMP accumulation	Antagonist	Single conc. (260 nM) reduced CRF potency by ~4.5-fold	21
**CXCR4**	Senkyunolide I	*Ligusticum chuanxiong*	n.d.	SPR, molecular docking	Antagonist	$K_d = 2.94\ \mu M$	22
**D2R**	Wilfortrine	*Tripterygium wilfordii*	G_i, β-arrestin	PiggyBac-TANGO, cAMP response	Agonist	$EC_{50} = 153.5$ nM (cAMP)	23
**D2R**	Atractylon	*Atractylodes macrocephala*	G_i, β-arrestin	PiggyBac-TANGO, cAMP response, *in vivo* activity assays	Agonist	$IC_{50} = 2.5\ \mu M$ (cAMP)	24
**D3R**	Icariside II	*Epimedium koreanum*	$G_{i/o}$	CLBA, cAMP accumulation, Ca^{2+} mobilization, molecular docking, *in vivo* activity assays	Agonist	$EC_{50} = 13.29\ \mu M$ (cAMP)	25
**M_2R**	Icaritin	*Epimedium koreanum*	$G_{i/o}$	CLBA, cAMP accumulation, Ca^{2+} mobilization, molecular docking, *in vivo* activity assays	Antagonist	Single conc. (100 μM) inhibited acetylcholine potency by 54.55%	25
**KOR**	O-methylcoclaurine (1), N-methylcoclaurine (2), coclaurine (3)	*Nelumbo nucifera*	n.d.	CLBA, *in vivo* activity assays	Agonist	$K_i = 3.5\ \mu M$ (1), 0.9 μM (2), 2.2 μM (3), 19.08 μM (4)	26

**DOR, MOR**	Neferine	*Nelumbo nucifera*	n.d.	CLBA, GTPγS binding assay	Agonist	DOR: K_i = 0.7 μM, EC_{50} = 7.9 μM, MOR: K_i = 1.8 μM, EC_{50} > 21 μM	26
**5-HT$_{1D}$R, 5-HT$_{2C}$R**	Hypericin (1), amentoflavone (2)	*Hypericum perforatum*	n.d.	CLBA	Antagonist	5-HT$_{1D}$R: IC_{50} = 4.094 μM (2), 5-HT$_{2C}$R: IC_{50} = 2.555 μM (2)	27
**D1R, D3R, D4R**	Hyperforin (1), amentoflavone (2), hypericin (3), pseudohypericin (4), quercetin (5)	*Hypericum perforatum*	n.d.	CLBA	Antagonist	D1R: IC_{50} = 0.59 μM (1), D3R: IC_{50} = 1.24 μM (2), 0.034 μM (3), 0.62 μM (4) D4R: IC_{50} = 1.47 μM (3), 1.27 μM (4), 7.84 μM (5)	27
**M5R**	Rutin	*Hypericum perforatum*	n.d.	CLBA	Antagonist	IC_{50} = 0.035 μM	27
**CRF1R**	Hypericin	*Hypericum perforatum*	n.d.	CLBA, GTPγS binding assay	Antagonist	IC_{50} = 300 nM	28
**DOR, KOR, MOR**	Hypericin (1), hyperforin (2)	*Hypericum perforatum*	n.d.	CLBA	Antagonist	DOR: IC_{50} = 4 μM (1), 0.5 μM (2), KOR: IC_{50} = 3 μM (1), 1 μM (2), MOR: IC_{50} = 1 μM (1), 0.4 μM (2)	28
**DOR, KOR, MOR, NOR**	Seven natural compounds	*Ganoderma cochlear*, *Carthamus tinctorius*	n.d.	DMR	Antagonist	IC_{50} = 10 - 300 μM	29

**FFAR1 (GPR40)**	Cyanidin-3-O-glucoside (1), delphinidin-3-O-glucoside (2)	*Zea mays*	$G_{q/11}$	IP1 accumulation, insulin secretion	Agonist	EC_{50} = 249 μM (1), 169.7 μM (2)	30
**FFAR1 (GPR40)**	Berberine	*Rhizoma Coptidis*	$G_{q/11}$	Ca^{2+} mobilization *in vivo* activity assays	Agonist	EC_{50} = 0.76 μM (Ca^{2+})	31
**FFAR1 (GPR40), GPR55**	Gintonin (glycolipoprotein complex)	*Panax ginseng*	$G_{q/11}$	Insulin secretion, Ca^{2+} mobilization	Agonist	Dose- and time-dependent stimulation of insulin secretion and Ca^{2+} transients	32
**FFAR1 (GPR40)**	Aloe emodin	*Rheum palmatum*	G_q	Molecular docking, *in vivo* activity assays	Antagonist	Single conc. (10, 20, 40 mg/kg) *in vivo* activity assays	33
**FFAR4 (GPR120)**	α-linolenic acid	*Perilla frutesccns*	G_q	Ca^{2+} imaging, ERK phosphorylation	Agonist	EC_{50} ≈ 0.6 μM (Ca^{2+})	34,35
**FPR2**	Columbamine	*Coptis chinensis*, *Jateorhiza palmata*	G_i, β-arrestin	cAMP response, Ca^{2+} mobilization, β-arrestin recruitment, molecular docking, *in vivo* activity assays	Agonist	EC_{50} ≈ 30 μM (cAMP)	36
**GPBAR1 (TGR5)**	Oleanolic acid	*Olea europaea*	n.d.	Luciferase reporter assay, *in vivo* activity assays	Agonist	EC_{50} = 1.42 μM	37
**GPBAR1 (TGR5)**	Ursolic acid	*Olea europaea*	n.d.	cAMP accumulation, GLP-1 secretion, *in vivo* activity assays	Agonist	EC_{50} = 4.18 μM	38,39
**GPBAR1 (TGR5)**	(+)-vitisinol E (1), paeoninol (2)	*Paeonia lactiflora*	MEK/ERK, IP3, Ca^{2+}/Ca MKII	cAMP accumulation, GLP-1 secretion, molecular docking	Agonists	Single conc. (50, 100 μM) increased cAMP contents with stimulative ratios more than 40%	40

**GPBAR1 (TGR5)**	Tauroursodeoxych-olic acid	Fel Ursi	n.d.	cAMP response, *in vivo* activity assays	Agonist	Single conc. (200 µM) increased cAMP production in time-dependent manner	41,42
**GPBAR1 (TGR5)**	Compound K (metabolite of ginsenosides)	*Panax ginseng*	G_s	Ca^{2+} mobilization, cAMP accumulation, GLP-1 secretion	Agonist	EC_{50} = 244 µM (Ca^{2+}) EC_{50} = 10.6 µM (cAMP)	43
**GPBAR1 (TGR5)**	Aqueous ethanolic extract	*Spergularia marina*	G_s	Ca^{2+} mobilization, cAMP accumulation, GLP-1 secretion	Agonist	EC_{50} = 63.4 µg/mL (cAMP)	44
**GPR30**	Genistein	*Genista tinctoria*, *Sophora subprostrala*	G_s	cAMP accumulation, *in vivo* activity assays	Agonist	Single conc. (2.5, 5 µM) stimulated cAMP production	45
**GPR55**	Curcumin	*Curcuma longa*	$G_{12/13}$	Luciferase reporter assays, Ca^{2+} mobilization, molecular docking	Agonist	Single conc. (10 µM) increased Ca^{2+} levels in a GPR55-dependent manner	46
**GPR97**	Curcumin	*Curcuma longa*	$G_{i/o}$	Luciferase reporter assays	Agonist	Single conc. (10, 20 µM) induced SRF-RE-mediated transcription in a GPR97-dependent manner	47
**GPR108**	Gambogic acid	*Garcinia hanburyi*	n.d.	Luciferase reporter assay, *in vivo* activity assays	Antagonist	n.d.	48,49
**GPRC6A**	Gallic acid (1), epigallocatechin 3-gallate (2)	*Camellia sinensis*	n.d.	ERK phosphorylation, molecular docking	Agonist (1), Antagonist (2)	Single conc. (50, 100 µM) inhibited or stimulated GPRC6A-mediated ERK phosphorylation	50

**KOR**	Salvinorin A	*Salvia divinorum*	$G_{i/o}$	CLBA, GTP turnover, cAMP inhibition	Agonist	K_i = 16 nM (CLBA), EC_{50} = 1 nM (cAMP), EC_{50} = 235 nM (GTPase)	51
**KOR**	Helianorphins-19	*Helianthus annuus*	G_i, β-arrestin	CLBA, cAMP inhibition, β-arrestin recruitment, *in vivo* activity assays	Agonist	K_i = 21 nM (CLBA), EC_{50} = 45 nM (cAMP) EC_{50} = 1.4 μM (β-arrestin)	52
**LPAR**	Gintonin (glycolipoprotein complex)	*Panax ginseng*	$G_{i/o}$, $G_{12/13}$, $G_{q/11}$	Ca^{2+} mobilization, ERK phosphorylation, cAMP response	Agonist	Binding affinity in order of LPA2 > LPA5 > LPA1 > LPA3 > LPA4 receptors	53
**M3R**	Hyoscyamine (1), scopolamine (2)	*Daturae flos*	n.d.	Affinity chromatography, HPLC-MS/MS	n.d.	n.d.	54
**mGluR5**	Monellin	*Dioscoreophy-llum cumminsii*	$G_{q/11}$	CLBA, Ca^{2+} mobilization, IP1 accumulation	Agonist	EC_{50} = 10 μM (Ca^{2+})	55
**MOR**	7-hydroxymitragynine (converted to mitragynine pseudoindoxyl *in vivo*)	*Mitragyna speciosa*	n.d.	CLBA， *in vivo* activity assays	Agonist	K_i = 77.9 nM (CLBA)	56
**MOR**	Ignavine	*Aconiti*	G_i	CLBA， cAMP inhibition, molecular docking, *in vivo* activity assays	Allosteric modulator	IC_{50} = 2 μM (CLBA), Single conc. (1 μM) induced 3.8-fold decrease of EC50 in response to DAMGO	57
**MRGPRX2**	Genistein	*Genista tinctoria*, *Sophora subprostrala*	β-arrestin	Ca^{2+} mobilization, PRESTO-Tango, molecular docking, *in vivo* activity assays	Antagonist	IC_{50} = 3.83 × 10^{-5} M (PRESTO-Tango), IC_{50} = 30 μM (Ca^{2+})	45,58

**MRGPRX2**	Osthole	*Cnidium monnieri*	n.d.	Ca^{2+} mobilization, molecular docking	Antagonist	Inhibited agonist-induced Ca^{2+} mobilization and degranulation in a dose-dependent fashion	59
**MT_1**	Oxyprenylated ferulic acid derivatives, umbelliferone derivatives	*Cassia cinnamon*	n.d.	CLBA	Agonist	K_i = 59.5 µM (4'-Gernayloxyferulic acid), 785.8 nM (Boropinal), 5.2 µM (7-iso-pentenyloxycoumarin), 1.9 µM (Auraotene), 3 nM (Umbellinprenin)	60
**MT_2**	Gastropolybenzyles	*Gastrodia elata*	n.d.	Ca^{2+} mobilization	Agonist	EC_{50} = 76 µM (Ca^{2+})	61
**MT_1, MT_2**	Polybenzyls	*Gastrodia elata*	n.d.	Ca^{2+} mobilization, molecular docking	Agonist	MT_1: EC_{50} = 237 µM, MT_2: EC_{50} = 244 µM	62
**MT_1, MT_2**	Catechin (1), epicatechin (2)	*Uncaria rhynchophylla*	n.d.	Ca^{2+} mobilization, *in vivo* activity assays	Agonist	MT_1: EC_{50} = 26 µM (1), 156 µM (2), MT_2: EC_{50} = 47 µM (1), 209 µM (2)	63
**NTSR**	Cyclopsychotride A	*Psychotria longipes*	n.d.	CBLA, Ca^{2+} mobilization	Agonist	IC_{50} = 3 µM (CBLA)	64
**OX1R, OX2R**	Neferine	*Plumula nelumbinis*	n.d.	SPR, molecular docking	Antagonist	OX1R: K_d = 2 nM (SPR), OX2R: K_d = 10 nM (SPR)	65

**TAS2R1, TAS2R8, TAS2R14**	Ligstroside aglycon (1), oleuropein aglycon (2)	*Olea europaea*	G_{gust}	Ca^{2+} mobilization	Agonist	TAS2R1: EC_{50} = 168.8 µM (1), 141.6 µM (2); TAS2R8: EC_{50} = 57.3 µM (1), 57.3 µM (2); TAS2R14: EC_{50} = 108.3 µM (1)	66,67
**TAS2R10, TAS2R14, TAS2R49**	Bergapten (1), xanthotoxin (2), isopimpinellin (3), skimmianine (4)	*Ruta graveolens*	n.d.	Ca^{2+} imaging	Agonist	TAS2R10: EC_{50} = 2.8 µM (1), 20.6 µM (2), 12 µM (3); TAS2R14: EC_{50} = 10.8 µM (2), 11.1 µM (3), 15.8 µM (4); TAS2R49: EC_{50} = 4 µM (4)	68
**TAS2R10**	Kudinoside A	*Ilex kudingcha*	G_{gust}	Ca^{2+} mobilization	Agonist	Single conc. (30 µM) induced Ca^{2+} release	69
**TAS2R14**	Picrotoxinin	*Ginkgo biloba*	G_{gust}	Ca^{2+} mobilization	Agonist	EC_{50} = 18 µM	70,71
**TAS2R14**	Apigenin (1), chrysin (2)	*Bee propolis* (1), *Scutellaria baicalensis* (2)	G_{gust}	Ca^{2+} mobilization	Agonist	Single conc. (30 µM (1), 120 µM (2)) induced Ca^{2+} release	72
**TAS2R14**	Aristolochic acid	*Aristolochia debilis*	G_i, G_{gust}	Ca^{2+} mobilization, G protein activation, Cryo-EM structure	Agonist	EC_{50} = 10.4 µM (Ca^{2+})	73
**TAS2R38**	Berberine	*Rhizoma coptidis*	n.d.	GLP-1 secretion assay, Ca^{2+} imaging	Agonist	Single conc. (100 µM) caused a rapid increase in Ca^{2+} concentration	74,75

**TAS2R46**	Quinine	*Cinchona calisaya*	G_{gust}	Ca^{2+} imaging	Agonist	EC_{50} = 10 μM	76,77
**TAS2R46**	Strychnine	*Strychnos nu-xvomica*	G_{gust}	Ca^{2+} mobilization, Cryo-EM structure	Agonist	EC_{50} = 0.43 μM	78,79
**TAS2R50**	Andrographolide (1), amarogentin (2)	*Andrographis paniculata*, *Gentiana scabra*	G_{gust}	Ca^{2+} mobilization	Agonist	EC_{50} = 22.9 μM (1), >300 μM (2)	80,81
**V_{1A}R, OTR**	Kalata B7	*Oldenlandia affinis*	$G_{q/11}$	CLBA, IP1 accumulation	Agonist	OTR: K_i = 50 μM, EC_{50} = 12 μM, V_{1A}R: K_i = 12 μM, EC_{50} = 4 μM	82
**α_{1A}-AR, α_{1B}-AR, α_{1D}-AR**	ρ-TIA	*Conus tulipa*	n.d.	CLBA	Antagonist	α_{1A}-AR: IC_{50} = 18 nM, α_{1B}-AR: IC_{50} = 2 nM, α_{1D}-AR: IC_{50} = 25 nM	83
**α_{2C}-AR**	Yohimbine	*Pausinystalia yohimbe*	n.d.	Molecular docking	Antagonist	pK_i = 9.01 μM	84
**β_1AR**	Pulegone	*Ziziphora clinopodioides*	n.d.	A-MS, molecular docking	Antagonist	n.d.	85
**β_2AR**	Ephedrine	*Pinellia ternate*, Qingfei Xiaoyan Wan	n.d.	UPLC/Q-TOF-MS, luciferase reporter assays	Agonist	Single conc. (100 μM) activated the receptor	86,87
**β_2AR**	Ephedrine	Chuanbeipipa dropping pills	n.d.	UPLC/Q-TOF-MS, luciferase reporter assay	Agonist	Single conc. (10 μM) activated the receptor	88

Note: The abbreviation of the targets refers to the GPCR database (https://gpcrdb.org/drugs/drugbrowser); **CLBA**, competitive ligand-binding assay;

SLCA, split luciferase complementation assay; **SPR**, surface plasmon resonance; **A-MS**, affinity mass spectrometry; **DMR**, Dynamic mass redistribution; **UPLC/Q-TOF-MS**, ultra-high performance liquid chromatography with quadrupole time-of-flight mass spectrometry; **HPLC-MS/MS**, liquid chromatography (HPLC)–mass spectrometry (MS); **IP1,** inositol monophosphate; **n.d.**, not detected; **Single conc.**, single concentration; **GTP**, guanosine triphosphate; **GLP-1**, glucagon-like peptide-1; **SRF-RE**, serum response factor-response element.

Table S2. Indirect effects of traditional medicines on the GPCR function over the last two decades

Effects	Molecules	Natural origin	Biological models	Detection methods	Potential pharmacological activity	References
Promoting GLP-1 secretion	*Gentiana scabra* root extract	*Gentiana scabra*	NCI-H716 cell, Lepr $^{-/-}$ (db/db) mice	GLP-1 releasing, Ca^{2+} imaging	Lowering blood glucose level	89
Enhancing the efficacy of melatonin	Ex18	*Pistacia vera*	HEK293 cell	CLBA, ERK activation, β-arrestin recruitment	Melatonin-potentiating activity	90
Activating Ca^{2+}/MAPK signaling pathway	α-Hederin	*Akebia trifoliata*	CRC cell, Nude male mice	Ca^{2+}mobilization, molecular docking, RT-qPCR	Inducing non-apoptotic cell death and paraptosis	91
Enhancing GLP-1 potency	N55	*Trigonella foenumgraecum*	U2OS, RINm5F, H460 cell	CLBA, cAMP accumulation, receptor endocytosis	Reducing glucose and glycated hemoglobin levels	92
Suppressing the activation of β-arrestin 1	Polysaccharides	*Dendrobium Officinaleon*	NCM460 cell, BalB/c mice	β-arrestin 1 expression, anti-inflammatory activity	Potential therapeutic effect on ulcerative colitis	93
Mediating β-arrestin 2 signaling	Total flavone	*Pollen Typhae*	C2C12 myoblast	Glucose uptake, β-arrestin 2 expression	Enhancing insulin-stimulated glucose uptake	94

Promoting GLP-1 secretion	Curcumin	*Curcuma longa*	GLUTag cell	Glucose tolerance, GLP-1 secretion	Improving glucose tolerance	95
Activate the cAMP pathway by binding to GNAS	Schizandrin A, formononetin, schisantherin A, notoginsenoside Ft1, gallic acid, ginsenoside F2, catalpol	Shenqi Jiangtang Granules	Glomerular mesangial cells	Bio-layer interferometry, UHPLC-Q/TOF-MS/MS, cAMP assay, Ca^{2+}-Mg^{2+}-ATPase level assay	Nephroprotective effect	96

Note: The abbreviation of the receptors refers to the GPCR database (https://gpcrdb.org/drugs/drugbrowser). **CLBA**, competitive ligand-binding assay; **GLP-1**, glucagon-like peptide-1; **MAPK,** Mitogen-activated protein kinase; **RT-qPCR**, quantitative real-time PCR; **UHPLC-Q/TOF-MS/MS**, ultra-high performance liquid chromatography with quadrupole time-of-flight mass spectrometry.

Table S3. FDA-approved nature-derived drugs targeting GPCRs

Drug	Target	Action	Natural origin	FDA approval year
**Cannabidiol**	Cannabinoid receptor 1	Antagonism	*Cannabis sativa*	2018
**Vorapaxar**	Protease-activated receptor 1	Antagonism	*Galbulimima baccata* (Derivative of himbacine)	2014
**Codeine**	Opioid receptors	Agonism	*Papaver somniferum*	2009
**Exenatide**	Glucagon-like peptide-1 receptor	Agonism	*Heloderma suspectum*	2005
**Caffeine**	Adenosine receptors	Antagonism	*Coffea arabica*	1999
**Dronabinol**	Cannabinoid receptors	Agonism	*Cannabis sativa*	1985
**Scopolamine**	Muscarinic acetylcholine receptors	Antagonism	*Datura stramonium*	1979
**Theophylline**	Adenosine receptors	Antagonism	*Camellia sinensis*	1976
**Pseudoephedrine**	Adrenergic receptors	Agonism	*Ephedra sinica*	1975
**Pilocarpine**	Muscarinic acetylcholine receptors	Agonism	*Pilocarpus microphyllus*	1974
**Atropine**	Muscarinic acetylcholine receptors	Antagonism	*Atropa belladonna*	1973
**Ephedrine**	Adrenergic receptors	Agonism	*Ephedra sinica*	1963
**Ergotamine**	Serotonin receptors; Alpha-1 adrenergic receptors	Agonism	*Claviceps purpurea*	1960

**Ergometrine**	Alpha-1A adrenergic receptor; Dopamine receptors; Serotonin receptors	Agonism	*Claviceps purpurea*	1946
**Hydrocodone**	Mu and delta opioid receptors	Agonism	*Papaver somniferum* (Derivative of Codeine)	1943
**Morphine**	Mu, delta and kappa opioid receptors	Agonism	*Papaver somniferum*	1941

Note: The listed drugs are from the Drugs@FDA database. (https://www.accessdata.fda.gov/scripts/cder/daf/index.cfm), and cross-referenced in the Drugbank (https://go.drugbank.com/), pharmacodia (https://www.pharmacodia.com/homeH5.html), and pharnexcloud database (https://www.pharnexcloud.com/). The abbreviation of the targets refers to the GPCR database (https://gpcrdb.org/drugs/drugbrowser).

References

1. Gafner S., Dietz B. M., McPhail K. L., et al. (2006). Alkaloids from *Eschscholzia californica* and Their Capacity to Inhibit Binding of [^{3}H]8-Hydroxy-2-(di-*N*-propylamino)tetralin to 5-HT_{1A} Receptors in Vitro. *J. Nat. Prod.* **69**:432-435. DOI:10.1021/np058114h
2. Nishi A., Yamaguchi T., Sekiguchi K., et al. (2012). Geissoschizine methyl ether, an alkaloid in Uncaria hook, is a potent serotonin1A receptor agonist and candidate for amelioration of aggressiveness and sociality by yokukansan. *Neuroscience* **207**:124-136. DOI:10.1016/j.neuroscience.2012.01.037
3. Madsen M. K., Fisher P. M., Burmester D., et al. (2019). Psychedelic effects of psilocybin correlate with serotonin 2A receptor occupancy and plasma psilocin levels. *Neuropsychopharmacol.* **44**:1328-1334. DOI:10.1038/s41386-019-0324-9
4. Dodd S., Norman T. R., Eyre H. A., et al. (2023). Psilocybin in neuropsychiatry: a review of its pharmacology, safety, and efficacy. *CNS Spectr.* **28**:416-426. DOI:10.1017/S1092852922000888
5. Zhang B., Zhao S., Yang D., et al. (2020). A Novel G Protein-Biased and Subtype-Selective Agonist for a G Protein-Coupled Receptor Discovered from Screening Herbal Extracts. *ACS Cent. Sci.* **6**:213-225. DOI:10.1021/acscentsci.9b01125
6. Dietz B. M., Mahady G. B., Pauli G. F., et al. (2005). Valerian extract and valerenic acid are partial agonists of the 5-HT_{5a} receptor in vitro. *Molecular Brain Research* **138**:191-197. DOI:10.1016/j.molbrainres.2005.04.009
7. Lacher S. K., Mayer R., Sichardt K., et al. (2007). Interaction of valerian extracts of different polarity with adenosine receptors: Identification of isovaltrate as an inverse agonist at A1 receptors. *Biochemical Pharmacology* **73**:248-258. DOI:10.1016/j.bcp.2006.09.029
8. Schumacher B., Scholle S., Hölzl J., et al. (2002). Lignans Isolated from Valerian: Identification and Characterization of a New Olivil Derivative with Partial Agonistic Activity at A1 Adenosine Receptors. *J. Nat. Prod.* **65**:1479-1485. DOI:10.1021/np010464q
9. Rukoyatkina N., Shpakova V., Bogoutdinova A., et al. (2022). Curcumin by activation of adenosine A_{2A} receptor stimulates protein kinase a and potentiates inhibitory effect of cangrelor on platelets. *Biochemical and Biophysical Research Communications* **586**:20-26. DOI:10.1016/j.bbrc.2021.11.006
10. Portilla-Martínez A., Ortiz-Flores M. Á., Meaney E., et al. (2022). (-)-Epicatechin Is a Biased Ligand of Apelin Receptor. *International Journal of Molecular Sciences* **23**:8962. DOI:10.3390/ijms23168962
11. Punyasiri P. A. N., Abeysinghe I. S. B., Kumar V., et al. (2004). Flavonoid biosynthesis in the tea plant *Camellia sinensis*: properties of enzymes of the prominent epicatechin and catechin pathways. *Archives of Biochemistry and Biophysics* **431**:22-30. DOI:10.1016/j.abb.2004.08.003
12. Zhu Y., Wu L., Zhao Y., et al. (2022). Discovery of oridonin as a novel agonist for BRS-3. *Phytomedicine* **100**:154085.

DOI:10.1016/j.phymed.2022.154085

13. Wei T.-T., Chandy M., Nishiga M., et al. (2022). Cannabinoid receptor 1 antagonist genistein attenuates marijuana-induced vascular inflammation. *Cell* **185**:1676-1693.e1623. DOI:10.1016/j.cell.2022.04.005
14. Skalicky M., Kubes J., Hejnak V., et al. (2018). Isoflavones Production and Possible Mechanism of Their Exudation in *Genista tinctoria* L. Suspension Culture after Treatment with Vanadium Compounds. *Molecules* **23**:1619. DOI:10.3390/molecules23071619
15. Ye B., Chen P., Lin C., et al. (2023). Study on the material basis and action mechanisms of *sophora davidii* (Franch.) skeels flower extract in the treatment of non-small cell lung cancer. *Journal of Ethnopharmacology* **317**:116815. DOI:10.1016/j.jep.2023.116815
16. LaVigne J. E., Hecksel R., Keresztes A., et al. (2021). Cannabis sativa terpenes are cannabimimetic and selectively enhance cannabinoid activity. *Scientific Reports* **11**:8232. DOI:10.1038/s41598-021-87740-8
17. Pandey P., Kumarihamy M., Chaturvedi K., et al. (2023). In Vitro and In Silico Studies of Neolignans from *Magnolia grandiflora* L. Seeds against Human Cannabinoids and Opioid Receptors. *Molecules* **28**:1253. DOI:10.3390/molecules28031253
18. Jiang X., Chen S., Zhang Q., et al. (2020). Celastrol is a novel selective agonist of cannabinoid receptor 2 with anti-inflammatory and anti-fibrotic activity in a mouse model of systemic sclerosis. *Phytomedicine* **67**:153160. DOI:10.1016/j.phymed.2019.153160
19. Chen S.-R., Dai Y., Zhao J., et al. (2018). A Mechanistic Overview of Triptolide and Celastrol, Natural Products from Tripterygium wilfordii Hook F. *Frontiers in Pharmacology* **9**. DOI:10.3389/fphar.2018.00104
20. Taghizadeh M. S., Retzl B., Muratspahić E., et al. (2022). Discovery of the cyclotide caripe 11 as a ligand of the cholecystokinin-2 receptor. *Scientific Reports* **12**:9215. DOI:10.1038/s41598-022-13142-z
21. Fahradpour M., Keov P., Tognola C., et al. (2017). Cyclotides Isolated from an Ipecac Root Extract Antagonize the Corticotropin Releasing Factor Type 1 Receptor. *Frontiers in Pharmacology* **8**:616. DOI:10.3389/fphar.2017.00616
22. Chen L., Lv D., Wang S., et al. (2020). Surface Plasmon Resonance-Based Membrane Protein-Targeted Active Ingredients Recognition Strategy: Construction and Implementation in Ligand Screening from Herbal Medicines. *Anal. Chem.* **92**:3972-3980. DOI:10.1021/acs.analchem.9b05479
23. Li F., Jiang X., Luo L.-L., et al. (2019). A piggyBac-based TANGO GFP assay for high throughput screening of GPCR ligands in live cells. *Cell Commun Signal* **17**:49. DOI:10.1186/s12964-019-0359-x
24. Li H., Wang F., Zhou Z., et al. (2022). Atractylon, a novel dopamine 2 receptor agonist, ameliorates Parkinsonian like motor dysfunctions in MPTP-induced mice. *NeuroToxicology* **89**:121-126. DOI:10.1016/j.neuro.2022.01.010

25. Seong S. H., Kim S. H., Ryu J. H., et al. (2023). Effects of Icariin and Its Metabolites on GPCR Regulation and MK-801-Induced Schizophrenia-Like Behaviors in Mice. *Molecules* **28**:7300. DOI:10.3390/molecules28217300

26. Kumarihamy M., León F., Pettaway S., et al. (2015). In vitro opioid receptor affinity and in vivo behavioral studies of *Nelumbo nucifera* flower. *Journal of Ethnopharmacology* **174**:57-65. DOI:10.1016/j.jep.2015.08.006

27. Butterweck V., Nahrstedt A., Evans J., et al. (2002). In vitro receptor screening of pure constituents of St. John's wort reveals novel interactions with a number of GPCRs. *Psychopharmacology* **162**:193-202. DOI:10.1007/s00213-002-1073-7

28. Simmen U., Higelin J., Berger-Büter K., et al. (2001). Neurochemical Studies with St. John's WortInVitro. *Pharmacopsychiatry* **34**:137-142. DOI:10.1055/s-2001-15475

29. Hou T., Xu F., Peng X., et al. (2021). Label-free cell phenotypic study of opioid receptors and discovery of novel mu opioid ligands from natural products. *Journal of Ethnopharmacology* **270**:113872. DOI:10.1016/j.jep.2021.113872

30. Luna-Vital D. A. and Gonzalez de Mejia E. (2018). Anthocyanins from purple corn activate free fatty acid-receptor 1 and glucokinase enhancing in vitro insulin secretion and hepatic glucose uptake. *PLOS One* **13**:e0200449. DOI:10.1371/journal.pone.0200449

31. Rayasam G. V., Tulasi V. K., Sundaram S., et al. (2010). Identification of berberine as a novel agonist of fatty acid receptor GPR40. *Phytotherapy Research* **24**:1260-1263. DOI:10.1002/ptr.3165

32. Cho Y.-J., Choi S.-H., Lee R., et al. (2020). Ginseng Gintonin Contains Ligands for GPR40 and GPR55. *Molecules* **25**:1102. DOI:10.3390/molecules25051102

33. Bao W., Lyu J., Feng G., et al. (2024). Aloe emodin promotes mucosal healing by modifying the differentiation fate of enteroendocrine cells via regulating cellular free fatty acid sensitivity. *Acta Pharmaceutica Sinica B*. DOI:10.1016/j.apsb.2024.05.027

34. Hirasawa A., Tsumaya K., Awaji T., et al. (2005). Free fatty acids regulate gut incretin glucagon-like peptide-1 secretion through GPR120. *Nat Med* **11**:90-94. DOI:10.1038/nm1168

35. Kim J. T., Lee S. B., Son M. J., et al. (2023). Perilla oil and α-linolenic acid ameliorated thrombosis in rats induced by collagen and epinephrine. *Food Sci Biotechnol* **32**:997-1003. DOI:10.1007/s10068-022-01241-6

36. Wu M. Y., Ge Y. J., Wang E. J., et al. (2023). Enhancement of efferocytosis through biased *FPR2* signaling attenuates intestinal inflammation. *EMBO Mol Med* **15**:e17815. DOI:10.15252/emmm.202317815

37. Sato H., Genet C., Strehle A., et al. (2007). Anti-hyperglycemic activity of a TGR5 agonist isolated from Olea europaea. *Biochemical and Biophysical*

Research Communications **362**:793-798. DOI:10.1016/j.bbrc.2007.06.130

38. Lo S.-H., Li Y., Cheng K. C., et al. (2017). Ursolic acid activates the TGR5 receptor to enhance GLP-1 secretion in type 1-like diabetic rats. *Naunyn-Schmiedeberg's Arch Pharmacol* **390**:1097-1104. DOI:10.1007/s00210-017-1409-9
39. Somova L. I., Shode F. O. and Mipando M. (2004). Cardiotonic and antidysrhythmic effects of oleanolic and ursolic acids, methyl maslinate and uvaol. *Phytomedicine* **11**:121-129. DOI:10.1078/0944-7113-00329
40. Wu S.-L., Zhang C.-C., Chen J.-J., et al. (2022). Oligostilbenes from the seeds of Paeonia lactiflora as potent GLP-1 secretagogues targeting TGR5 receptor. *Fitoterapia* **163**:105336. DOI:10.1016/j.fitote.2022.105336
41. Yanguas-Casás N., Barreda-Manso M. A., Nieto-Sampedro M., et al. (2017). TUDCA: An Agonist of the Bile Acid Receptor GPBAR1/TGR5 With Anti-Inflammatory Effects in Microglial Cells. *Journal Cellular Physiology* **232**:2231-2245. DOI:10.1002/jcp.25742
42. Wang X., Wu H., Li M., et al. (2024). A Comprehensive Analysis of Fel Ursi and Its Common Adulterants Based on UHPLC-QTOF-MSE and Chemometrics. *Molecules* **29**:3144. DOI:10.3390/molecules29133144
43. Kim K., Park M., Lee Y. M., et al. (2014). Ginsenoside metabolite compound K stimulates glucagon-like peptide-1 secretion in NCI-H716 cells via bile acid receptor activation. *Arch. Pharm. Res.* **37**:1193-1200. DOI:10.1007/s12272-014-0362-0
44. Kim K., Lee Y. M., Rhyu M.-R., et al. (2014). *Spergularia marina* Induces Glucagon-Like Peptide-1 Secretion in NCI-H716 Cells Through Bile Acid Receptor Activation. *Journal of Medicinal Food* **17**:1197-1203. DOI:10.1089/jmf.2013.3091
45. Luo J., Wang A., Zhen W., et al. (2018). Phytonutrient genistein is a survival factor for pancreatic β-cells via GPR30-mediated mechanism. *The Journal of Nutritional Biochemistry* **58**:59-70. DOI:10.1016/j.jnutbio.2018.04.018
46. Harada N., Okuyama M., Teraoka Y., et al. (2022). Identification of G protein-coupled receptor 55 (GPR55) as a target of curcumin. *npj Sci Food* **6**:4. DOI:10.1038/s41538-021-00119-x
47. Harada N., Arahori Y., Okuyama M., et al. (2022). Curcumin activates G protein-coupled receptor 97 (GPR97) in a manner different from glucocorticoid. *Biochemical and Biophysical Research Communications* **595**:41-46. DOI:10.1016/j.bbrc.2022.01.075
48. Lyu S., Zhang X., Tu Z., et al. (2022). GPR108 is required for gambogic acid inhibiting NF-κB signaling in cancer. *Pharmacological Research* **182**:106279. DOI:10.1016/j.phrs.2022.106279
49. Liu Y., Chen Y., Lin L., et al. (2020). Gambogic Acid as a Candidate for Cancer Therapy: A Review. *IJN* **Volume 15**:10385-10399. DOI:10.2147/IJN.S277645

50. Pi M., Kapoor K., Ye R., et al. (2018). GPCR6A Is a Molecular Target for the Natural Products Gallate and EGCG in Green Tea. *Molecular Nutrition Food Res* **62**:1700770. DOI:10.1002/mnfr.201700770

51. Roth B. L., Baner K., Westkaemper R., et al. (2002). Salvinorin A: A potent naturally occurring nonnitrogenous κ opioid selective agonist. *Proceedings of the National Academy of Sciences* **99**:11934-11939. DOI:10.1073/pnas.182234399

52. Muratspahić E., Tomašević N., Koehbach J., et al. (2021). Design of a Stable Cyclic Peptide Analgesic Derived from Sunflower Seeds that Targets the κ-Opioid Receptor for the Treatment of Chronic Abdominal Pain. *Journal of Medicinal Chemistry* **64**:9042-9055. DOI:10.1021/acs.jmedchem.1c00158

53. Hwang S. H., Shin T.-J., Choi S.-H., et al. (2012). Gintonin, Newly Identified Compounds from Ginseng, Is Novel Lysophosphatidic Acids-Protein Complexes and Activates G Protein-Coupled Lysophosphatidic Acid Receptors with High Affinity. *Molecules and Cells* **33**:151-162. DOI:10.1007/S10059-012-2216-z

54. Fan H., Huang X., Zhang Z., et al. (2023). Immobilization of M3 Muscarinic Receptor to Rapidly Analyze Drug—Protein Interactions and Bioactive Components in a Natural Plant. *International Journal of Molecular Sciences* **24**:7171. DOI:10.3390/ijms24087171

55. Chen A. N. Y., Hellyer S. D., Trinh P. N. H., et al. (2020). Identification of monellin as the first naturally derived proteinaceous allosteric agonist of metabotropic glutamate receptor 5. *Basic Clin Pharma Tox* **126**:104-115. DOI:10.1111/bcpt.13239

56. Obeng S., Leon F., Patel A., et al. (2022). Interactive Effects of μ-Opioid and Adrenergic-α_2 Receptor Agonists in Rats: Pharmacological Investigation of the Primary Kratom Alkaloid Mitragynine and Its Metabolite 7-Hydroxymitragynine. *J Pharmacol Exp Ther* **383**:182-198. DOI:10.1124/jpet.122.001192

57. Ohbuchi K., Miyagi C., Suzuki Y., et al. (2016). Ignavine: a novel allosteric modulator of the μ opioid receptor. *Scientific Reports* **6**:31748. DOI:10.1038/srep31748

58. Kumar M., Singh K., Duraisamy K., et al. (2020). Protective Effect of Genistein against Compound 48/80 Induced Anaphylactoid Shock via Inhibiting MAS Related G Protein-Coupled Receptor X2 (MRGPRX2). *Molecules* **25**:1028. DOI:10.3390/molecules25051028

59. Callahan B. N., Kammala A. K., Syed M., et al. (2020). Osthole, a Natural Plant Derivative Inhibits MRGPRX2 Induced Mast Cell Responses. *Frontiers in Immunology* **11**:703. DOI:10.3389/fimmu.2020.00703

60. Hasan M., Genovese S., Fiorito S., et al. (2017). Oxyprenylated Phenylpropanoids Bind to MT1 Melatonin Receptors and Inhibit Breast Cancer Cell Proliferation and Migration. *J. Nat. Prod.* **80**:3324-3329. DOI:10.1021/acs.jnatprod.7b00853

61. Chen S.-Y., Geng C.-A., Ma Y.-B., et al. (2019). Melatonin Receptors Agonistic Activities of Phenols from Gastrodia elata. *Nat. Prod. Bioprospect.* **9**:297-302. DOI:10.1007/s13659-019-0213-2

62. Chen S.-Y., Geng C.-A., Ma Y.-B., et al. (2019). Polybenzyls from Gastrodia elata, their agonistic effects on melatonin receptors and structure-activity relationships. *Bioorganic & Medicinal Chemistry* **27**:3299-3306. DOI:10.1016/j.bmc.2019.06.008

63. Geng C.-A., Yang T.-H., Huang X.-Y., et al. (2019). Antidepressant potential of Uncaria rhynchophylla and its active flavanol, catechin, targeting melatonin receptors. *Journal of Ethnopharmacology* **232**:39-46. DOI:10.1016/j.jep.2018.12.013

64. Witherup K. M., Bogusky M. J., Anderson P. S., et al. (1994). Cyclopsychotride A, a Biologically Active, 31-Residue Cyclic Peptide Isolated from Psychotria longipes. *J. Nat. Prod.* **57**:1619-1625. DOI:10.1021/np50114a002

65. He J., Fang J., Wang Y., et al. (2023). Discovery of Small-Molecule Antagonists of Orexin 1/2 Receptors from Traditional Chinese Medicinal Plants with a Hypnotic Effect. *Pharmaceuticals* **16**:542. DOI:10.3390/ph16040542

66. Cui M., Chen B., Xu K., et al. (2021). Activation of specific bitter taste receptors by olive oil phenolics and secoiridoids. *Scientific Reports* **11**:22340. DOI:10.1038/s41598-021-01752-y

67. Baccouri B., Rajhi I., Theresa S., et al. (2022). The potential of wild olive leaves (*Olea europaea* L. subsp. oleaster) addition as a functional additive in olive oil production: the effects on bioactive and nutraceutical compounds using LC–ESI–QTOF/MS. *Eur Food Res Technol* **248**:2809-2823. DOI:10.1007/s00217-022-04091-y

68. Mancuso G., Borgonovo G., Scaglioni L., et al. (2015). Phytochemicals from Ruta graveolens Activate TAS2R Bitter Taste Receptors and TRP Channels Involved in Gustation and Nociception. *Molecules* **20**:18907-18922. DOI:10.3390/molecules201018907

69. Zhou Y.-W., Sun J., Wang Y., et al. (2022). Tas2R activation relaxes airway smooth muscle by release of Gα_t targeting on AChR signaling. *Proceedings of the National Academy of Sciences* **119**:e2121513119. DOI:10.1073/pnas.2121513119

70. Behrens M., Brockhoff A., Kuhn C., et al. (2004). The human taste receptor hTAS2R14 responds to a variety of different bitter compounds. *Biochemical and Biophysical Research Communications* **319**:479-485. DOI:10.1016/j.bbrc.2004.05.019

71. Ng C. C., Duke R. K., Hinton T., et al. (2017). Effects of bilobalide, ginkgolide B and picrotoxinin on $GABA_A$ receptor modulation by structurally diverse positive modulators. *European Journal of Pharmacology* **806**:83-90. DOI:10.1016/j.ejphar.2017.04.019

72. Hariri B. M., McMahon D. B., Chen B., et al. (2017). Flavones modulate respiratory epithelial innate immunity: Anti-inflammatory effects and activation of the T2R14 receptor. *Journal of Biological Chemistry* **292**:8484-8497. DOI:10.1074/jbc.M116.771949

73. Hu X., Ao W., Gao M., et al. (2024). Bitter taste TAS2R14 activation by intracellular tastants and cholesterol. *Nature* **631**:459-466. DOI:10.1038/s41586-024-07569-9

74. Yu Y., Hao G., Zhang Q., et al. (2015). Berberine induces GLP-1 secretion through activation of bitter taste receptor pathways. *Biochemical Pharmacology* **97**:173-177. DOI:10.1016/j.bcp.2015.07.012

75. Yang W.-L., Zhang C.-Y., Ji W.-Y., et al. (2024). Berberine Metabolites Stimulate GLP-1 Secretion by Alleviating Oxidative Stress and Mitochondrial Dysfunction. *Am. J. Chin. Med.* **52**:253-274. DOI:10.1142/S0192415X24500113

76. Grassin-Delyle S., Abrial C., Fayad-Kobeissi S., et al. (2013). The expression and relaxant effect of bitter taste receptors in human bronchi. *Respiratory Research* **14**:134. DOI:10.1186/1465-9921-14-134

77. do Amaral V. L. L., Frajbat M., Petreanu M., et al. (2014). Reproductive toxicology and clastogenic evaluation in mice of a phytotherapeutic formulation obtained from *Cinchona calisaya* Weddel (Rubiaceae) used in Brazilian folk medicine as female fertility stimulant. *Journal of Ethnopharmacology* **155**:1508-1512. DOI:10.1016/j.jep.2014.07.038

78. Xu W., Wu L., Liu S., et al. (2022). Structural basis for strychnine activation of human bitter taste receptor TAS2R46. *Science* **377**:1298-1304. DOI:10.1126/science.abo1633

79. Brockhoff A., Behrens M., Massarotti A., et al. (2007). Broad Tuning of the Human Bitter Taste Receptor hTAS2R46 to Various Sesquiterpene Lactones, Clerodane and Labdane Diterpenoids, Strychnine, and Denatonium. *J. Agric. Food Chem.* **55**:6236-6243. DOI:10.1021/jf070503p

80. Behrens M., Brockhoff A., Batram C., et al. (2009). The Human Bitter Taste Receptor hTAS2R50 Is Activated by the Two Natural Bitter Terpenoids Andrographolide and Amarogentin. *J. Agric. Food Chem.* **57**:9860-9866. DOI:10.1021/jf9014334

81. Sheeja K., Guruvayoorappan C. and Kuttan G. (2007). Antiangiogenic activity of Andrographis paniculata extract and andrographolide. *International Immunopharmacology* **7**:211-221. DOI:10.1016/j.intimp.2006.10.002

82. Koehbach J., O'Brien M., Muttenthaler M., et al. (2013). Oxytocic plant cyclotides as templates for peptide G protein-coupled receptor ligand design. *Proceedings of the National Academy of Sciences* **110**:21183-21188. DOI:10.1073/pnas.1311183110

83. Chen Z., Rogge G., Hague C., et al. (2004). Subtype-selective Noncompetitive or Competitive Inhibition of Human α1-Adrenergic Receptors by ρ-TIA. *Journal of Biological Chemistry* **279**:35326-35333. DOI:10.1074/jbc.M403703200

84. Hai-Bo L., Yong P., Lu-qi H., et al. (2013). Mechanism of Selective Inhibition of Yohimbine and Its Derivatives in Adrenoceptor *α*2 Subtypes. *Journal of Chemistry* **2013**:1-9. DOI:10.1155/2013/783058

85. Shayiranbieke A., Liang Q., Wang T., et al. (2022). Development of immobilized beta1-adrenoceptor chromatography for rapid discovery of ligands specifically binding to the receptor from herbal extract. *Journal of Chromatography A* **1677**:463298. DOI:10.1016/j.chroma.2022.463298

86. Dong L., Cheng B., Luo Y., et al. (2014). Identification of Nuclear Factor-κB Inhibitors and β_2 Adrenergic Receptor Agonists in Chinese Medicinal Preparation Fuzilizhong Pills Using UPLC with Quadrupole Time-of-flight MS. *Phytochemical Analysis* **25**:113-121. DOI:10.1002/pca.2474

87. Cheng B., Hou Y., Wang L., et al. (2012). Dual-bioactivity-based liquid chromatography-coupled quadrupole time-of-flight mass spectrometry for NF-κB inhibitors and β_2AR agonists identification in Chinese Medicinal Preparation Qingfei Xiaoyan Wan. *Analytical and Bioanalytical Chemistry* **404**:2445-2452. DOI:10.1007/s00216-012-6332-9

88. Dong L., Luo Y., Cheng B., et al. (2013). Bioactivity-integrated ultra-performance liquid chromatography/quadrupole time-of-flight mass spectrometry for the identification of nuclear factor-*κ*B inhibitors and β_2 adrenergic receptor agonists in Chinese medicinal preparation Chuanbeipipa dropping pills. *Biomedical Chromatography* **27**:960-967. DOI:10.1002/bmc.2886

89. Suh H.-W., Lee K.-B., Kim K.-S., et al. (2015). A bitter herbal medicine Gentiana scabra root extract stimulates glucagon-like peptide-1 secretion and regulates blood glucose in db/db mouse. *Journal of Ethnopharmacology* **172**:219-226. DOI:10.1016/j.jep.2015.06.042

90. Labani N., Gbahou F., Noblet M., et al. (2023). Pistacia vera Extract Potentiates the Effect of Melatonin on Human Melatonin MT1 and MT2 Receptors with Functional Selectivity. *Pharmaceutics* **15**:1845. DOI:10.3390/pharmaceutics15071845

91. Rao X., Li Z., Zhang Q., et al. (2024). α-Hederin induces paraptosis by targeting GPCRs to activate Ca^{2+}/MAPK signaling pathway in colorectal cancer. *Cancer Medicine* **13**:e7202. DOI:10.1002/cam4.7202

92. King K., Lin N.-P., Cheng Y.-H., et al. (2015). Isolation of Positive Modulator of Glucagon-like Peptide-1 Signaling from *Trigonella foenum-graecum* (Fenugreek) Seed. *Journal of Biological Chemistry* **290**:26235-26248. DOI:10.1074/jbc.M115.672097

93. Liang J., Chen S., Chen J., et al. (2018). Therapeutic roles of polysaccharides from *Dendrobium Officinaleon* colitis and its underlying mechanisms. *Carbohydrate Polymers* **185**:159-168. DOI:10.1016/j.carbpol.2018.01.013

94. Feng X.-T., Wang T.-Z., Chen Y. I., et al. (2012). Pollen Typhae total flavone improves insulin-induced glucose uptake through the β-arrestin-2-mediated signaling in C2C12 myotubes. *International Journal of Molecular Medicine* **30**:914-922. DOI:10.3892/ijmm.2012.1061

95. Kato M., Nishikawa S., Ikehata A., et al. (2017). Curcumin improves glucose tolerance via stimulation of glucagon-like peptide-1 secretion. *Molecular Nutrition Food Res* **61**:1600471. DOI:10.1002/mnfr.201600471

96. Zhang H., Yao J., Xiao G., et al. (2024). Discovery of drug targets based on traditional Chinese medicine microspheres (TCM-MPs) fishing strategy combined with bio-layer interferometry (BLI) technology. *Analytica Chimica Acta* **1305**:342542. DOI:10.1016/j.aca.2024.342542
